# Normothermic perfusion of human livers for profiling lentiviral vector pharmacokinetics and transduction

**DOI:** 10.1016/j.omta.2025.201660

**Published:** 2025-12-26

**Authors:** Brannon R.M. Nicholls, David Johnson, Anurag Kulkarni, Rui André Saraiva Raposo, Kyriacos A. Mitrophanous, Constantin C. Coussios, Robert C. Carlisle

**Affiliations:** 1 Institute of Biomedical Engineering, Department of Engineering Science, University of Oxford, Botnar 3 Research Building, Windmill Road, Headington, Oxford OX3 7LD, UK; 2 Oxford Biomedica (UK) Ltd, Windrush Court, Transport Way, Oxford OX4 6LT, UK

**Keywords:** lentiviral vector, gene therapy, liver-directed gene therapy, normothermic machine perfusion, *ex vivo*, pharmacokinetics, pharmacodynamics, liver, pre-clinical model

## Abstract

Lentiviral vectors (LVs) hold significant potential for gene therapy (GT) due to their ability to integrate into non-dividing cells, potentially offering lifelong cures from a single dose. The liver is an attractive GT target due to its role in inherited disorders and as a sink for intravenously delivered therapeutics. However, clinical translation of LV therapy remains challenging due to the poor predictive value of animal models. Normothermic machine perfusion (NMP) maintains human organs under physiological conditions *ex vivo*, creating an opportunity to reduce reliance on animal studies and de-risk human clinical trials. We used NMP to assess pharmacokinetics and transduction in four human livers dosed with an LV encoding green fluorescent protein (GFP). Perfusion was maintained for up to 74 h, achieving physiological viability and function. Rapid LV clearance was observed, with less than 1% remaining in perfusate after 10 min. Integrated viral copy number per cell reached 0.07–0.13, with detectable GFP expression. Transcriptomic analysis revealed dynamic changes in metabolic and inflammatory pathways correlating with liver function and transduction outcomes. This study demonstrates that NMP provides a useful model to assess LV delivery and transduction, supporting its potential as a platform to enhance translation into human clinical applications.

## Graphical abstract

**Figure:**
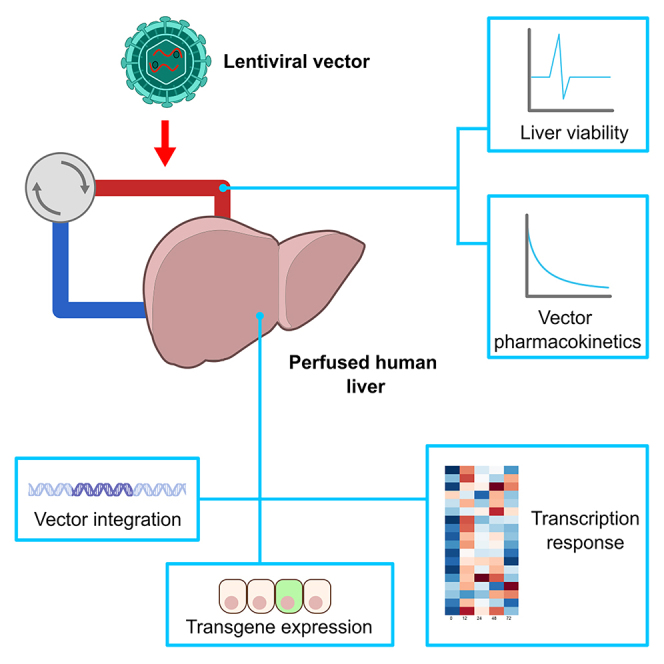

## Teaser

Human livers were maintained by normothermic machine perfusion and dosed with a lentiviral gene therapy vector. Vector clearance, integration, and transgene expression were profiled, along with inflammatory and transcriptomic responses. The work demonstrated the utility of perfused organs as a preclinical model for lentiviral gene therapies.

## Introduction

Lentiviral vectors (LVs) are of particular interest for use in gene therapies (GTs) due to their ability to transduce quiescent cells and integrate transgenes into the target cell’s genome, promoting long-term therapeutic expression and potentially offering a lifelong cure for genetic disorders.^1^ LVs also possess sufficient capacity for multiple genes to be delivered, and a low rate of patient preexposure reduces the likelihood of pre-existing antibody-based immunity to LVs,^2^ an issue with many adeno-associated virus (AAV) vectors.^3^^,^^4^^,^^5^ However, neutralization in human serum still occurs through complement responses to common LV pseudotypes and allogeneic responses to producer cell membrane proteins such as major histocompatibility complex.^6^^,^^7^^,^^8^ Such responses have contributed to a lack of development of LV GT vectors for *in vivo* use following intravenous (IV) delivery.

Although LVs are used extensively for *ex vivo* GT, the clinical landscape *in vivo* remains poor: while there are nine *ex vivo* LV GTs approved by the US Food and Drug Administration (FDA) or European Medicines Agency (EMA), there are none approved for IV delivery.^9^^,^^10^ There are signs of improvement: three phase I clinical trials using IV LV delivery for *in vivo* generation of CAR-T cells are underway,^11^ but the scientific and clinical progress made with *ex vivo* delivery has yet to be matched.

The liver is a popular target for *in vivo* GT as it is the site of many genetic disorders such as hemophilia and alpha-1 antitrypsin deficiency.^12^ Addressing such diseases with LV is dependent on limited neutralization in blood, evasion of liver resident macrophages, Kupffer cells (KCs), and efficient transduction and integration into the genome of hepatocytes. Notably, the liver is the major clearance site for bloodborne particles and can remove and inactivate >90% of circulating virus following IV delivery.^13^ Understanding LV delivery and capture in human livers is therefore a prerequisite for developing GTs with both hepatic and extra-hepatic targets. Test models of appropriate scale and anatomy for this are lacking, despite studies in large animal models including pigs and non-human primates (NHPs).^14^^,^^15^^,^^16^ A review by Baruteau et al. highlighted a number of examples where animal studies of AAV liver-directed GTs failed to predict outcomes in human patients,^17^ and such findings are likely to also be observed for LV. Without accurate recapitulation of human anatomy and physiology there is a risk that safety and efficacy evidence gathered in mice and NHP models will be as misleading for LV as it is for other classes of drug: just 10% of new pharmaceuticals that successfully navigate preclinical studies ultimately gain approval for human use.^18^ Notably, 50% of this unsustainable attrition rate is due to a lack of efficacy, highlighting an industry-wide need for alternative preclinical models.^19^

One potential alternative to animal models is the use of normothermic machine perfusion (NMP) to maintain human organs under physiological conditions *ex vivo*. Commercially available NMP devices have been approved for clinical use and demonstrated to increase preservation time, reduce graft injury, and improve patient outcomes for higher-risk livers when compared with static cold storage (SCS).^20^ Recent studies have used NMP to study the delivery and liver cell distribution of AAVs and identify serotypes with preferential infection of hepatocytes.^21^^,^^22^ To date, no studies have used the approach to profile LV pharmacokinetics (PK) and pharmacodynamics (PD).

In this study, we administered a reporter-encoding LV to human donor livers maintained by NMP and profiled clearance from circulation, viral entry and reverse transcription, integration, and transgene expression. Furthermore, we obtained temporally discrete data from tissue biopsies throughout the perfusion following a sampling regimen, which would not be possible or permissible in animal models or patients. In this manner, we provide insights into the effective delivery of LV to human organs while adding to the growing body of evidence supporting use of NMP organs as the most relevant and effective preclinical models.

## Results

### Liver function maintained for up to 70 h following LV administration

**Figure 1:**
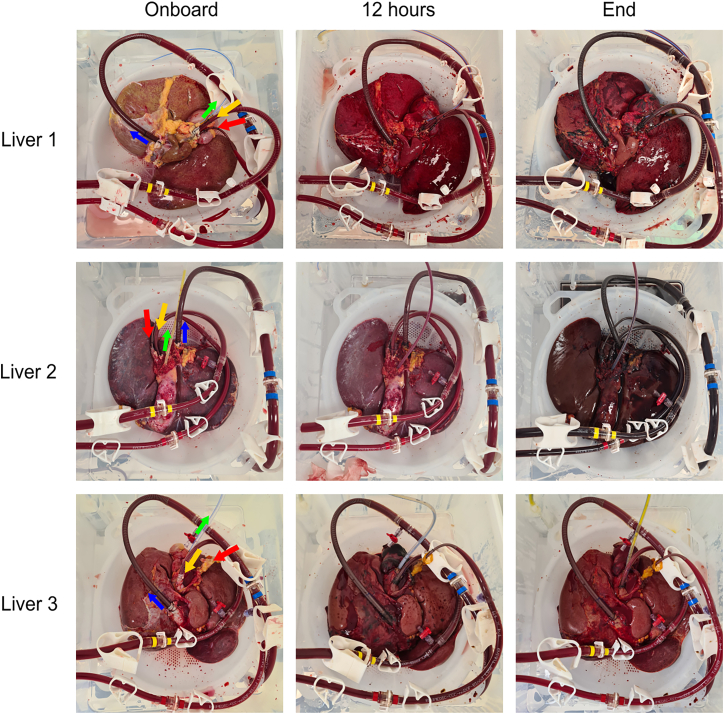
Normothermic machine perfusion of whole human livers Four whole human livers were dosed with LV and maintained by NMP for up to 74 h. Top-down view of livers connected to the NMP device throughout perfusion. Arrows indicate vessel tubing and flow direction: red, hepatic artery; yellow, hepatic portal vein; blue, inferior vena cava; green, bile duct. Following perfusion livers change from pale/blue to a deep red signifying the successful re-establishment of flow and health.

Four whole human livers classified as suitable for donation at point of resection (L1, L2, L3, and L4) but subsequently deemed unsuitable for transplant (Table 1) were obtained from National Health Service Blood and Transplant (NHSBT) and connected to the NMP device as described in the methods (Figure 1).

**Table 1:** Description of perfused human livers

Liver	Patient death	Reason declined for transplant	Condition notes	Weight (kg)	Patient age (years)	Patient sex (M/F)	Total ischemia time (HH:MM)	Total perfusion duration (HH:MM)	Perfusion duration after dose (HH:MM)	Reason for perfusion end
L1	DBD	Fatty	Round edges, mildly steatotic	2.2	74	M	13:14	70:56	68:52	Excessive bleeding post biopsy
L2	DCD	Function on NRP	AST increased during normothermic regional perfusion	Not recorded	58	M	12:22	74:03	71:13	Increasing lactate
L3	DBD	Anatomy	Longitudinal hematoma, arterial thrombosis, mildly fibrotic	1.614	78	F	13:09	55:22	51:22	Increasing lactate
L4	DCD	Untransplantable	Calcification of hepatic artery, low-moderately fatty, underperfused segment VII	2.18	66	M	13:44	66:40	64:34	Increasing lactate

Four whole human livers were maintained by normothermic machine perfusion following rejection for transplant. DBD: donation after brainstem death, the patient has been declared dead based on neurological criteria but circulation continues. DCD: donation after circulatory death, the patient has been declared dead after cessation of circulatory and respiratory function. DCD organs may be exposed to longer warm ischemia times, increasing the risk of organ damage. NRP: normothermic regional perfusion, whereby circulation and oxygenation of an organ is restored *in situ* using extracorporeal assistance.

For NMP to provide a useful model of liver LV capture and transduction the perfused liver must meet normal human health and function parameters. The NMP device provides a range of outputs enabling liver function to be tracked and controlled, which can be complemented by blood gas and chemistry measurements using separate blood analyzers.

**Figure 2:**
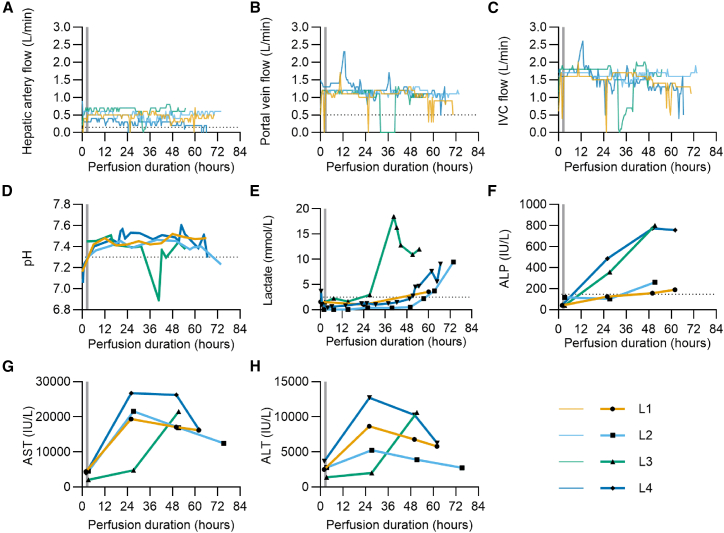
Metrics of liver function. Four discarded whole human livers (“L1” to “L4”) were maintained by normothermic machine perfusion and dosed with lentiviral vector (A) Hepatic artery blood flow. (B) Portal vein blood flow. (C) Inferior vena cava (IVC) blood flow. (D) Perfusate pH. (E) Perfusate lactate concentration. (F) Perfusate alkaline phosphatase (ALP) concentration. (G) Perfusate aspartate aminotransferase (AST) concentration. (H) Perfusate alanine aminotransferase (ALT) concentration. Dotted lines in (A), (B), and (D) indicate minimum values, and dotted line in (E) indicates maximum value required to meet viability criteria described by Mergental et al.^23^ Dotted line in (F) indicates the maximum normal range for patient ALP. Normal ranges for AST and ALT not shown due to scale of y axis.

After establishment of hemodynamics and blood chemistry in the normal physiological range (achieved in under four hours for all livers), each liver was dosed with GFP-expressing LV and perfusion maintained for up to 74 h (Table 1). Mergantal et al. proposed that one of two major criteria (bile production or lactate concentration below 2.5 mmol/L) and two or more minor criteria (blood pH > 7.3, glucose metabolism, hepatic arterial flow >150 mL/min, portal vein (PV) flow >500 mL/min, or homogeneous perfusion) must be met by donor livers receiving NMP to predict successful function in transplant recipients,^23^ and these criteria were used to assess liver viability in this study (as indicated by the dotted lines in Figure 2).

As shown in Figures 2A–2C, all livers achieved flow rates in or close to the physiological range for the majority of perfusion. Average arterial flow was 0.47, 0.49, 0.63, and 0.28 L/min (SD 0.12, 0.10, 0.14, 0.10) for L1-L4, respectively, and average portal flow was 1.04, 1.13, 0.97, and 1.23 L/min (SD 0.19, 0.09, 0.40, 0.24), well above the thresholds proposed by Mergental et al.^23^ and in line with normal *in vivo* flow rates (normal adult total hepatic blood flow is between 1.5 and 1.9 L/min, two-thirds of which is supplied by the hepatic PV and the remainder by the hepatic artery [HA]).^24^ Normal flow for L3 was temporarily interrupted between 31 and 39 h after perfusion start due to an infusion pump fault, which altered perfusate volume and pressure, with no PV flow received during that period. Normal flow rates were resumed; however, this malfunction likely contributed to the premature decline of L3.

Normal blood pH is between 7.35 and 7.45. Perfusate was acidotic for L1, L2, and L4 at the start of perfusion, correcting to the normal range in under 6 h, while L3 began at pH 7.45 (Figure 2D). L3 pH dropped severely within the period of interrupted perfusate flow but was rapidly returned to the normal range upon the resumption of normal flow rates.

Lactate clearance has been established as a reliable indicator of liver function.^25^ Perfusate lactate concentration for all four livers remained below 2.5 mmol/L, the suggested threshold value, for the first 24 h after perfusion start (Figure 2E). By 39 h, L3 lactate had dramatically risen to nearly 20 mmol/L, almost certainly a result of the interrupted flow rates previously described. It soon decreased below 10 mmol/L; however, after 54 h the concentration continued to increase: this strongly suggests a suboptimally functioning liver beyond 15 h, the last time point when lactate was falling and was below 2.5 mmol/L. In contrast, L1 and L2 lactate only increased beyond 2.5 mmol/L after 60 h and L4 after 50 h, indicating normal metabolic activity throughout the majority of perfusion.

The enzymes alkaline phosphatase (ALP), alanine aminotransferase (ALT), and aspartate aminotransferase (AST) are serum biomarkers of liver damage and have commonly been used as surrogate endpoints for long-term graft survival in clinical studies of liver transplantation.^26^^,^^27^ For all livers, ALP was within the healthy range (<147 IU/L) prior to dosing but increased beyond that by 50 h (Figure 2F).^28^ ALP was particularly high at approximately 800 IU/L for L3 and L4 at 50 h, whereas the increase was more gradual for L1 and L2. Prior to dosing, all three livers displayed levels of AST and ALT above the normal patient range^29^^,^^30^ but in line with expectations after 2–3 h of reperfusion (Figures 2G and 2H).^31^^,^^32^^,^^33^^,^^34^ The substantially raised levels observed in samples taken at 24 h post-LV dosing may represent damage resulting from LV infection or delayed reperfusion injury responses as ALT and AST levels greater than 1,000 IU/L can be indicative of severe liver damage from a number of causes such as ischemia, drug-induced damage, or viral hepatitis.^35^

All perfusions were maintained beyond 48 h, extending to 74 h for L2. L1 perfusion was ended due to excessive post-biopsy bleeding, and suturing of biopsy wounds was adopted to limit this in the following two perfusions. Perfusion of L2, L3, and L4 was ended due to increasing lactate concentrations. Overall, physiological flow rates combined with pH maintenance and lactate clearance suggest normal conditions were maintained throughout the majority of perfusion, and liver damage biomarkers, although high, did show signs of recovery.

### LV is rapidly removed from circulation and achieves replicable transduction in NMP human livers

**Figure 3:**
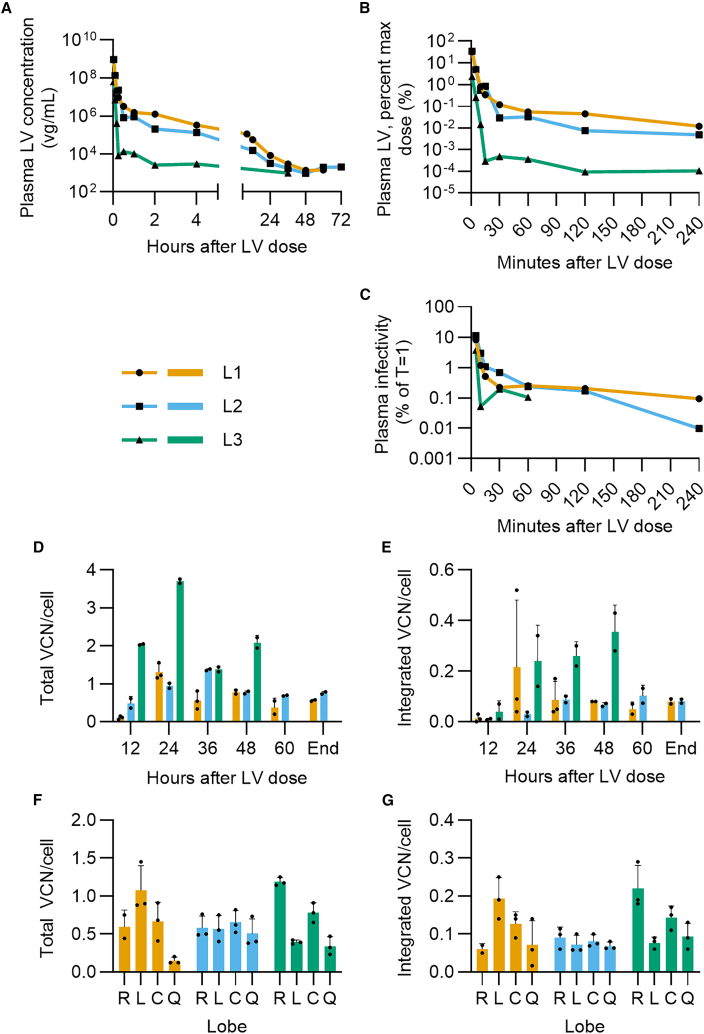
Pharmacokinetics, integration, and distribution of LV administered to NMP livers (A) LV genome (vg) concentration in plasma fraction of perfusate following administration of 5.8 × 10^10^ TU (3.34 × 10^12^ vg) to perfused human livers, first time point at 1 min after dose. (B) LV genome concentration in perfusate as a percentage of the theoretical maximum concentration, calculated from measured LV concentration relative to input LV genomes using a perfusate volume of 1,200 mL and assuming homogenous distribution of LV in the perfusate. (C) Infectivity of plasma as a percentage of T = 1 transducing concentration (TU/mL). Plasma TU/mL was determined by *in vitro* infectivity assay and is presented as a percentage of T = 1 to account for different sample treatment, which may have affected measured infectivity (L1 plasma was subjected to an additional round of freeze-thaw after storage). (D) Total VCN/cell (integrated and non-integrated) and (E) integrated VCN/cell of liver tissue taken from the right lobe of each liver every 12 h via core biopsies. (F) Total VCN/cell and (G) integrated VCN/cell of tissue from each liver lobe at the end of perfusion. For (D) to (G), black circles indicate biopsy technical replicates, bars represent the mean, error bars indicate standard deviation. For (F) and (G), R, L, C, and Q represent the Right, Left, Caudate, and Quadrate lobes, respectively, the four lobes of the human liver.

Following normalization of blood flow and biochemistry, L1-L3 were dosed with 5.8 × 10^10^ transducing units (TU) (3.34 × 10^12^ RNA vector genomes, vg) LV with GFP transgene under control of a hepatocyte-specific promoter in a 20 mL bolus to the HA. This dosing was based on a previous experiment performed on isolated porcine-derived liver (Clark et al. under review). The fourth liver “L4” received a 5-fold lower dose to investigate linearity of PK and PD outputs and the potential for dose sparing. The PK was defined in terms of both vector RNA copy number and transducing vector concentration circulating in plasma. RNA copy number was quantified by reverse-transcriptase digital PCR (RT-dPCR) of serially collected plasma samples (Figures 3A and 3B), while the transducing titer was determined by application of plasma samples to *in vitro* cell monolayers and subsequent quantification of integrated LV genomes by serial passaging and dPCR (Figure 3C), a method adapted from that used by Moore-Kelly et al.^36^

The theoretical maximum dose, presuming homogenous dissolution of the whole dose within the total perfusate volume, was calculated by division of the total input RNA vector genomes by the total perfusate volume. Following administration, LV genomes were rapidly cleared from L1, L2, and L3, with plasma concentration dropping more than 100-fold in the first 10 min to below 1% of the maximum dose (Figure 3B). By 4 h, less than 0.1% of the maximum dose was in circulation. Clearance for L1 and L2 is biphasic, with an initial fast half-life of 1.42 and 1.32 min (Table S1). One-phase decay followed by a plateau appears a better fit for L3, with a half-life of 1.26 min. Following this the clearance rate slowed, and RNA genomes continued to circulate at extremely low levels for the remainder of all perfusions. Application of the same dose to the perfusion device without a liver connected established that there is minimal LV loss to the system when a liver is not present (Figure S1), suggesting that the rapid decline in LV genome concentration observed is in fact a result of uptake by the liver. Transduction of cell monolayers *in vitro* was used to validate the RT-dPCR data and also probe the loss or retention of activity in the circulating LV dose (Figures 3C and S2). Clearance of actively transducing LV (TU/mL) closely reflects the observed changes in vg/mL.

In L4, dosing at one-fifth of the dose used in L1-3 led to no LV genomes being detectable beyond 60 min (Figure S3), indicating some saturation of a clearance mechanism may be achieved at the higher dose. LV genomes were detected in L1 bile at 8 h after dosing but had greatly reduced by 24 h. For L2-L4, little or no LV was detected in bile throughout perfusion (Figure S4).

Having defined clearance from the circulation, total vector copy number (VCN)/cell and integrated VCN/cell were quantified from tissue samples taken from the right lobe of each liver every 12 h after dosing. Total VCN includes both integrated and non-integrated vector DNA copies in cells, while integrated VCN refers to only those vector DNA copies that have inserted into the host cell genome. All livers exhibited a peak in total VCN/cell, between 24 and 36 h, before subsequently declining (Figure 3D). L1 and L2 peaked at 1.3 and 1.4 VCN/cell before declining to 0.6 and 0.8 by the end of perfusion, while L3 had a greater peak of 3.7 VCN/cell at 24 h, which subsequently declined to 2.1 by 48 h. It may be that the more rapid plasma clearance observed for L3 resulted in greater delivery to tissues and increased infection.

Integrated VCN/cell was established by quantification of vector genomes in isolated genomic DNA as described in the methods (Figure 3E).^37^ Integrated VCN/cell peaked at 24 h for L1, reaching 0.2 before stabilizing until the end of perfusion. L2 did not exhibit such a peak but increased until 36 h and then remained level at 0.08–0.09 until the end of perfusion, whereas L3 VCN/cell continued to increase to 0.36 by 48 h after which no further samples were available.

At the end of perfusion, VCN/cell was calculated from samples across all lobes (Figures 3F and 3G). VCN/cell varied across lobes, but integrated VCN/cell was only significantly different between the right and left and right and quadrate lobes of L3 (Tables S2, S3, S4, and S5). Average total VCN/cell at endpoint was very similar between L1 and L3 at 0.621 (SD 0.381) for L1, 0.577 (SD 0.062) for L2, and 0.675 (SD 0.393) for L3, showing no significant difference between perfusions despite the more rapid clearance rate observed in L3 perfusate (Tables S6 and S7). Mean integrated VCN/cell also showed no significant difference at 0.113, 0.078, and 0.133 for L1, L2, and L3, respectively (SD 0.061, 0.010, 0.064) (Tables S8 and S9), equivalent to transduction of 11%, 8%, and 13% of cells. L4 demonstrated similar transduction trends to L1-L3 but achieved lower VCN/cell at endpoint of 0.057 (total) and 0.007 (integrated), 5–10% of the VCN achieved by L1-L3 and in line with the lower dose received (Figures S3D–S3G).

### Cytokine profiles reveal pre-existing inflammation variability

**Figure 4:**
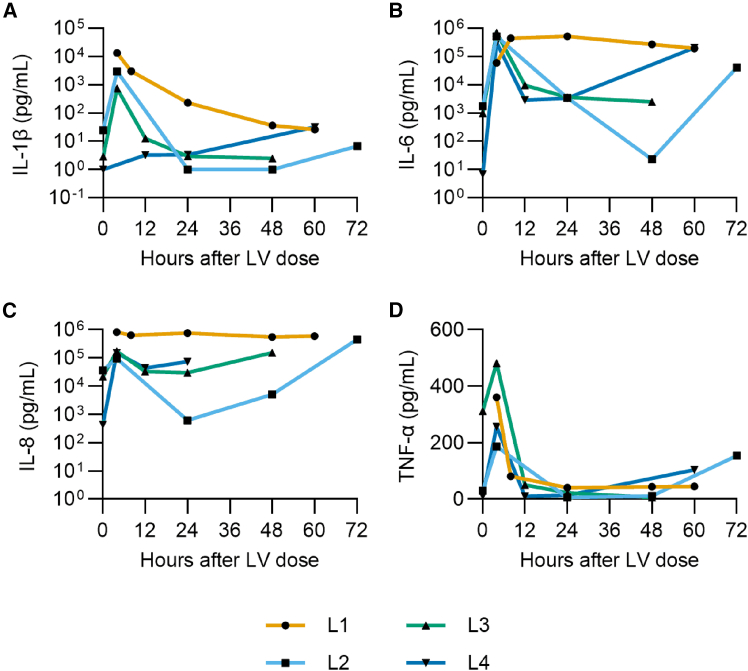
Cytokine profiles of NMP livers dosed with LV Filtrate was collected from the hemoconcentrator throughout each perfusion and cytokine concentration quantified with a multiplex assay cassette. (A) IL-1β, (B) IL-6, (C) IL-8, (D) TNF-α. LV dose was given at time 0; time 0 samples were collected immediately prior to dosing.

Analysis of cytokine levels may provide information on background health status of donated organs, levels of surgical and perfusion injury, and/or the impact of LV dosing. Inflammatory cytokines in filtrate collected from the hemoconcentrator were quantified via a multiplex cassette system as described in the methods (Figure 4). For most livers, all cytokines exhibited a peak at 4 h after dosing, suggesting a strong immune response resulting from vector administration. L2 and L3 interleukin-1 beta (IL-1β) increased 100-fold within 4 h of dosing, from 25 pg/mL and 3 pg/mL to 2,955 pg/mL and 748 pg/mL, respectively. L1 peaked at 13,150 pg/mL at 4 h, and all three declined below time 0 concentration by 48 h. In contrast, IL-1β concentration of L4 gradually increased throughout, from <1 pg/mL at time 0 to 32 pg/mL by 60 h. This could reflect the lower dose received by L4.

L2, L3, and L4 interleukin-6 (IL-6) all peak at greater than 300,000 pg/mL at 4 h after dosing before declining approximately 100-fold by 24 h, again suggesting activation of an anti-viral response. By the end of perfusion L2 and L4 IL-6 increased. This may be due to liver condition rather than in response to LV dose, as IL-6 is strongly associated with liver damage response pathways with both pro- and anti-inflammatory functions.^38^ Interleukin-8 (IL-8) follows a similar pattern, with a peak at 4 h for L2-4 and a gradual increase from 24 h onwards.

Similar to the other cytokines, tumor necrosis factor alpha (TNF-α) peaked at 4 h and then decreased for all livers up to 48 h, increasing again for L2 and L4 beyond 60 h, which could reflect declining viability. The initial high concentration of TNF-α for L3 may reflect a more inflamed liver condition, and it is possible that higher inflammation may have increased leakiness of L3 vasculature and contributed to the faster clearance of vector from plasma previously described.

### GFP expression detectable in perfused livers by 51 h

**Figure 5:**
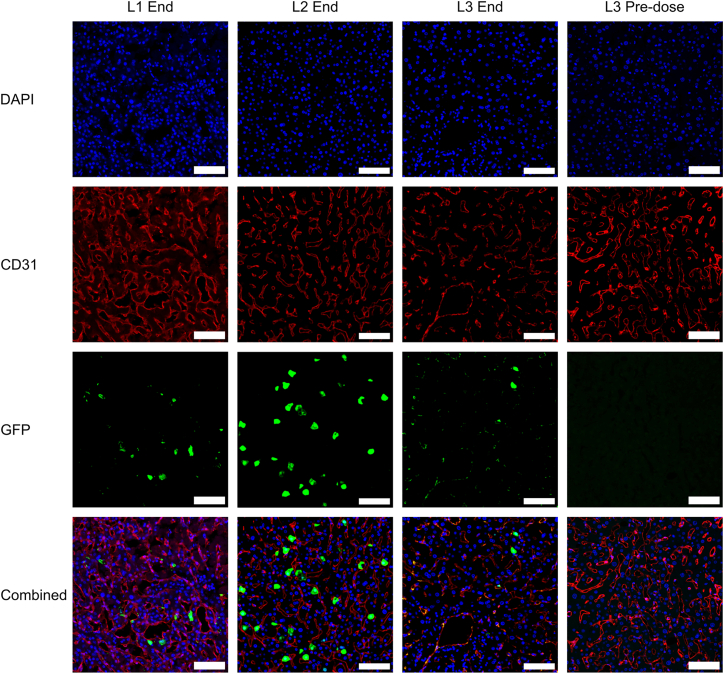
GFP expression detectable in NMP livers dosed with LV GFP expression in tissue of L1, L2, and L3. Following perfusion tissue segments from L1 were cryopreserved in OCT compound and 10 μm sections produced. Segments from L2 and L3 were preserved by FFPE and 4 μm sections produced. All sections were stained with DAPI (blue) and antibodies against GFP (green) and the epithelial marker CD31 (red). L3 Pre-dose biopsy taken during perfusion shortly before dosing with LV. Scale bars, 50 μm.

Sections from the right lobes of L1-L3 were fluorescently stained for GFP to assess the level and distribution of GFP transgene expression (Figure 5). The sections were also stained for the epithelial marker CD31 to highlight the complex vascular structure of the liver.

GFP expression was detected across all livers, confirming successful transduction of tissue in all perfusions (Figures 5 and S5). Expression was most extensive in L2 with GFP detectable in approximately 4% of L2 cells but in fewer than 1% of L1 and L3 cells despite L2 having the lowest and L3 the highest integrated VCN/cell (Table S10). This may be partly explained by the extended perfusion duration achieved by L2 providing time for increased transcription and translation of the GFP transgene. The difference in L2 integration and expression (7% vs. 4%) suggests that not all transduced cells are expressing GFP at a sufficient level to be detectable. This is expected as the vector is pseudotyped with a broad tropism vesicular stomatitis virus G protein (VSV-G) envelope and the transgene is under control of a hepatocyte-specific promoter, although, interestingly, L3 exhibited some GFP expression in CD31-positive cells.

Analysis of the spatial deposition of GFP expression within liver lobes was performed as described in the methods. The data shown in Figure S6 demonstrate that expression is achieved in equivalent levels for the entire span between the portal triad and the central vein. This is an interesting finding running contrary to other studies of nanoparticle distribution in the liver lobule,^39^ which may be due to differences in the test species used (i.e., rat) or the “hardness” of the materials used.

### Gene set variation coincides with observed liver function and inflammation

**Figure 6:**
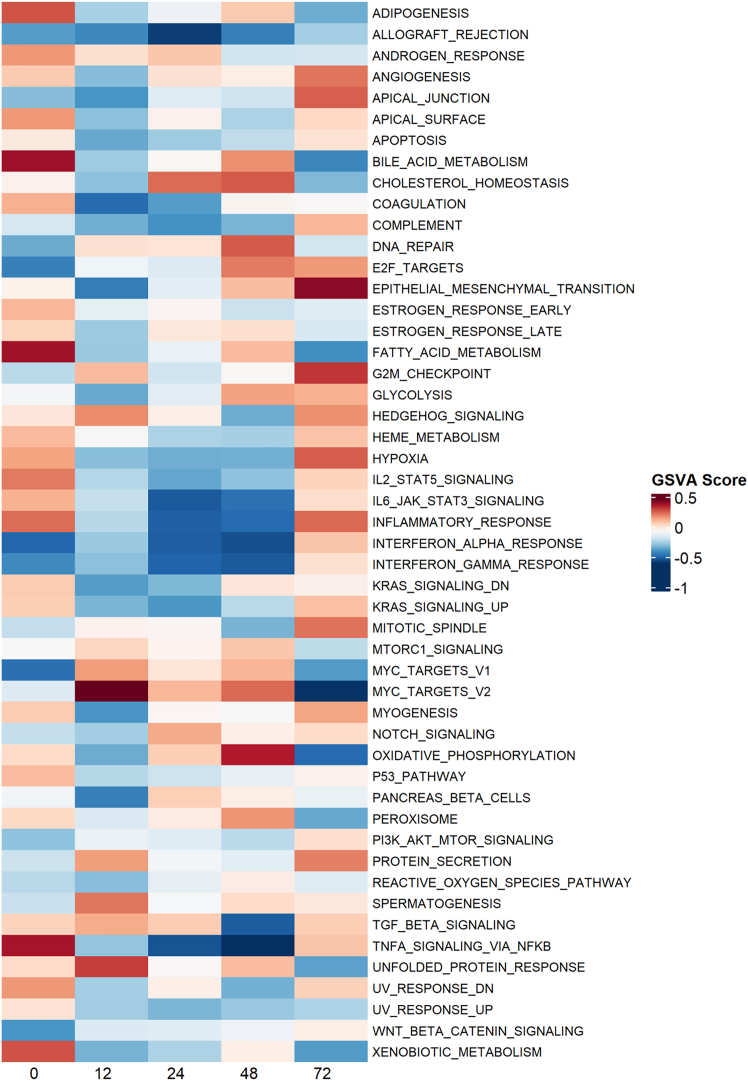
Gene set variation analysis of Hallmark pathways in L2 Total mRNA from L2 tissue samples was isolated and sequenced and gene set variation analysis performed using MSigDB Hallmark gene sets. Tissue samples were taken at 0 (pre-dose), 12, 24, 48, and 72 h after dosing with LV.

**Figure 7:**
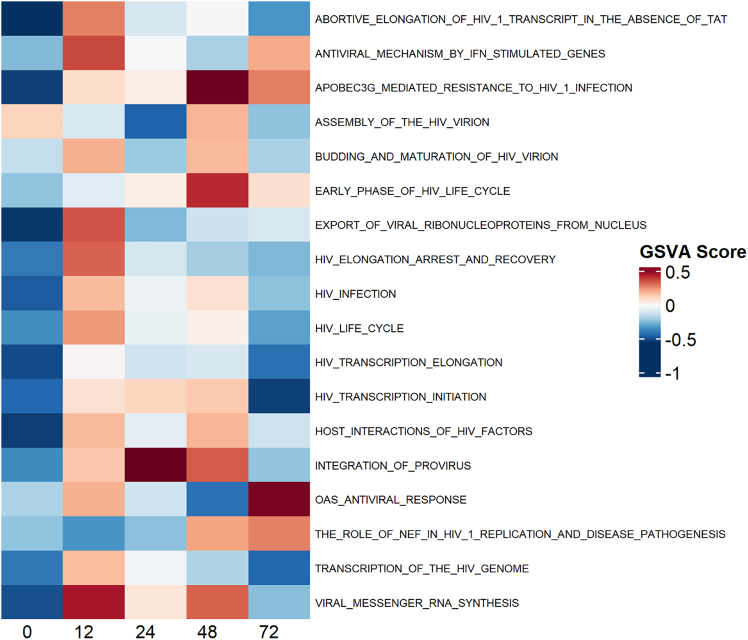
Gene set variation analysis of viral Reactome pathways in L2 Total mRNA from L2 tissue samples was isolated and sequenced and gene set variation analysis performed using MSigDB Reactome gene sets. Presented data were filtered for terms “HIV” and “viral.” Tissue samples were taken at 0 (pre-dose), 12, 24, 48, and 72 h after dosing with LV.

RNA extraction and mRNA sequencing of tissue samples from L2 was performed (Figures 6 and 7). Gene set enrichment analysis was performed using the Molecular Signatures Database (MSigDB) Hallmark collection^40^^,^^41^^,^^42^ to identify pathway expression changes throughout perfusion relative to a tissue sample taken immediately prior to dosing with LV.

At time 0, metabolic pathways including those associated with oxidative phosphorylation and fatty acid metabolism were upregulated, likely indicating an increase in metabolism in response to the cessation of SCS and the initiation of NMP. At 72 h, these pathways were inhibited and those associated with hypoxia and glycolysis were upregulated, coinciding with increased lactate levels and denoting a change in the metabolic profile of cells at later time points. Between 48 and 72 h pathways associated with cell-cycle regulation and differentiation (e.g., G2M checkpoint and epithelial-mesenchymal transition) were upregulated, suggesting a process of repair and tissue restructuring had started.

Inflammatory response pathways were activated prior to dosing and at the end of perfusion, probably a reflection of the liver’s inflamed state following harvest and transport and an increase in inflammation as its condition deteriorated toward the end of perfusion. In particular, IL-6 and TNF-α signaling corresponds with observed changes in cytokine concentration. The decreasing oxidative metabolism and increased inflammation pathways combined with the increased lactate and inflammatory cytokines observed support the notion that liver health was in decline by 72 h.

Interferon responses demonstrated a modest activation at 12 h relative to time 0, followed by strong activation at 72 h. Initial activation at 12 h was likely in response to LV delivery. The resurgence at 72 h may be in response to production of the LV transgenes in transduced cells, or a result of an increasingly inflamed environment.

Gene set variation analysis (GSVA) was also performed with the MSigDB Reactome collection, which details 1,736 gene sets versus the 50 contained in the Hallmark collection and so should enable a more granular analysis of signaling pathways. GSVA results were filtered for terms pertaining to HIV and viral infection response (Figure 7). As expected, nearly all HIV-associated pathways were inactive prior to dosing. At 12 h after LV dosing there was activation of both viral infection and antiviral pathways such as interferon and APOBEC3G antiviral responses. These are reflective of both active transduction of cells and the innate immune response raised against the vector.

Between 12 and 48 h there was an increase in provirus integration pathways, corresponding to the increase in integrated VCN/cell observed. By 72 h most viral life cycle pathways were downregulated and antiviral responses were again increased, coinciding with the interferon response identified with the Hallmark gene sets.

## Discussion

NMP is now a well-established procedure that can maintain liver viability between donation and transplant and improve outcomes for patients receiving from higher-risk DCD (donation after circulatory death) donors.^20^^,^^43^^,^^44^ Recently, there has been increasing interest in use of perfused organs as pre-clinical models as the only way to truly recapitulate the anatomy and function of a human patient.^21^^,^^22^ Indeed, the approach allows a level of sampling and analysis that is not practically or ethically feasible in clinical trials, an important feature when profiling the PK and PD of advanced biologic therapies. The growing availability of commercial perfusion systems also makes NMP livers an increasingly available research tool.

Lentiviral GTs have great promise for long-term treatment of liver resident and extra-hepatic inherited and acquired disorders, but IV delivery of LV to patients is still in its infancy. In this study, we demonstrate that NMP livers are capable of profiling the PK and transduction of a liver-directed lentiviral GT vector, thereby demonstrating the utility of the system and also gaining insights into LV delivery and activity.

Four human livers were maintained by NMP for up to 74 h. Defined against the limits suggested by Mergental et al.,^23^ three of the four livers were functionally viable for the majority of perfusion duration, and perfusion was ended following deviation from these limits. The duration achieved did not match the 13 days reported by Lau et al.^45^ but was sufficient to observe LV transduction and integration events, as well as transgene expression (as evidenced by the stabilization of integrated VCN/cell after 48 h) and GFP fluorescence.

Following dosing, rapid clearance from the perfusate was observed. Perfusate LV genome concentration declined over 100-fold within 10 min, faster clearance than that observed with an AAV vector in a similar, albeit open, perfusion system, which experienced a decline to 9% of the initial vector concentration after 24 h^21^ Through binding the low-density lipoprotein receptor, VSV-G confers the LV with a broad tropism that likely contributes to its rapid removal from the NMP system.^46^ This tropism includes KCs, which, combined with their phagocytic ability, makes them primarily responsible for LV clearance and limiting hepatocyte transduction.^47^ High levels of transduction by LV are well documented in human macrophages *in vitro* and in mouse KCs following IV delivery.^16^^,^^48^^,^^49^ Lower KC transduction has been reported in primates, although this is typically after dosing with “stealthy” immune-evading vectors,^16^^,^^49^ and it remains to be seen how much KC transduction is achieved in human patients or in a whole human liver setting. Observed clearance also surpassed reported rates for LV in some animal models. Delivery of 2 × 10^9^ TU/kg of HIV-derived vector to NHPs resulted in a half-life of 1 h,^50^ much longer than the sub-2 min rates observed here. This discrepancy may relate to differing total doses leading to saturation of clearance mechanisms in the NHP experiments, which may not have been achieved here; however, the total dose in the NHP study was not specified. In addition, the first NHP time point was taken at 30 min after dosing, so substantial initial vector clearance may have been missed. Alternatively, it may relate to physiological differences in the livers of the two species. We have already observed that LV experiments in perfused porcine livers (Clark et al., under review) gave extended circulation compared with the results reported here. Porcine liver has been reported to have a smaller fenestrated endothelial gap size (82 nm) vs. human (107 nm), which could impede hepatocyte access in the porcine model and comparatively reduce clearance.^51^ Similar effects may be expected in the NHP model (gap size reported as 77–82 nm in baboons), but regardless it is clear that our studies raise concerns about the scientific validity of using large animal models to profile liver clearance of all viral and non-viral nanoscale therapeutics.

The NMP system enabled the regular retrieval of tissue biopsies throughout perfusion, a feat not easily attainable in animal models or human clinical trials patients, which facilitated LV transduction to be profiled over time. Total VCN/cell peaked at 24 h for L1 and L3 and at 36 h for L2. Similar 24-h peaks have been reported in hematopoietic stem cells transduced with LV *ex vivo*.^52^ Integration was detectable by 12 h after dosing in all livers. Livers 1 and 2 exhibited a rise and fall, with peak integration at 24 and 36 h, respectively, while L3 showed a continued increase until 48 h. The explanation for the peak and subsequent decline in integrated genomes remains unclear. It may be due to immune removal of transduced cells or shedding of KCs, which primarily clear particles entering the liver. It may also be the consequence of initially improved perfusion of this lobe; however, the variability in the three separately taken biopsies suggests the high mean value may be skewed by one anomalous reading. Although the initial pathology of the livers, the perfusion durations, and the consequent infection profiles differed across the replicates, the average total and integrated copy numbers in tissue were remarkably similar, equivalent to between 7 and 13% of cells being transduced by the end of perfusion with no statistically significant difference. It was noticeable that deeper tissue analysis of L3 produced a lower LV copy number than surface sampling with a biopsy needle. In future studies we advise that needle biopsy should be the standardized sampling technique throughout perfusion.

By end of perfusion GFP expression was observed in all livers. Highest expression level was observed in L2, likely a result of the longer perfusion duration achieved. Application of more cell-specific stains and flow cytometry methods may better elucidate cell types successfully expressing the transgene and enable better assessment of vector transduction specificity. Comparison of the GFP % positivity (up to 4%) with the integrated VCN percentage (7–13%) suggests that there may be a pool of non-hepatocyte cells with integrated LV but without the capacity to express the GFP transgene under the control of the hepatocyte-specific enhanced transthyretin (ET) promotor.^49^ GFP expression was observed in CD31-positive cells of L3 alone, suggesting tightness of promoter control may vary between patients. Endothelia in L3 may also have been more permissive to the vector than for other livers, enabling a higher uptake by liver sinusoidal endothelial cells (LSECs) and contributing toward L3’s rapid plasma clearance and higher peak VCN during perfusion. This supports the continuation of research efforts to achieve consistent retargeting of LV at both the level of LV interaction with the cell surface (i.e., through tropism modification)^16^^,^^53^^,^^54^ as well as by engineering for enhanced promoter selectivity.^55^^,^^56^^,^^57^

Filtrate from the hemoconcentrator was also sampled to titrate inflammatory cytokines and gauge the immune environment of the perfused livers. A peak in inflammatory cytokines was observed at 4 h after dosing (Figure 4), possibly in response to vector delivery. Peak concentrations of IL-1β, IL-6, and IL-8 were higher than those reported at similar time points in non-dosed perfusion studies.^31^^,^^58^ In recently published clinical trial data whereby a CAR-T LV was administered IV, three of four patients exhibited spikes in plasma IL-6 at 24 or 48 h, reaching between 300 and 12,000 pg/mL, a similar range to the 24 h samples here.^59^ In that trial, cytokines were not measured at 4 h after dosing; however, immediately after LV infusion all patients developed acute inflammatory reactions, with three patients developing cytokine release syndrome, suggesting high levels of IL-6 (and other cytokine) release may have occurred shortly after dosing as was observed in this perfused liver model.

In our study, while L1 demonstrated consistently high levels of IL-6 and IL-8, L2 and L4 exhibited a decrease at 12–24 h followed by raised IL-1, IL-6, IL-8, and TNF-α at the end of perfusion, potentially indicating the deteriorating and inflamed condition of the liver that coincides with a breakdown in function as indicated by increasing lactate. Many cytokines, for example, IL-6, are implicated in both the liver’s acute phase response to damage as well as immune response to infection,^60^^,^^61^ and discerning between the underlying impact of surgical and reperfusion injury and the impact of LV infection in these circumstances can be challenging.

From Figures 2G and 2H it is clear that initial AST and ALT rise substantially between pre-LV dose levels and 24 h post-dosing. It is expected that these damage markers would be higher than in a normal healthy patient: the livers have been surgically recovered and spent over 12 h without circulation. Furthermore, the livers were all rejected for transplant precisely because of their suboptimal condition (Table 1), for example, the presence of steatosis or hematomas; therefore, some transaminase elevation is not unexpected, and levels in excess of 9,000 IU/mL have been observed following transplant with successful patient recovery.^62^ The decline evident in transaminase levels of livers L1, L2, and L4 between 24 h and endpoint could be indicative of resolution of hepatocyte health. To address this, we have compared the T1/2 for transaminase removal as published by Kim et al.^63^ and found that our T1/2 for ALT matches while our T1/2 for AST is greater than the published value (Table S11). The matching ALT half-life could indicate resolution of hepatocyte health. Indeed, during the clinical trial described by Nasralla et al., which examined liver transplant outcomes following organ preservation by NMP,^20^ patient ALT and AST peaked within seven days of transplant but only returned to normal range after six months, so a relatively slow rate of transaminase clearance is not unexpected even when the long-term outcome is positive. What is not so clear is whether initial rises up to 24 h are the consequence of LV dosing and transduction or reperfusion injury. Data from Eshmuminov et al. and Mohamed et al. showing lower transaminase levels at 24 h than reported here indicate that the former is more likely.^34^^,^^64^

Inclusion of a non-LV dosed control would have helped definitively separate inflammation and toxicity events caused by LV dosing from those caused by re-perfusion injury. However, multiple studies indicate that perfusion stabilizes cytokines and enzymes reversing transplant and cold storage injury^31^^,^^32^^,^^58^^,^^65^; hence, time and resources were not directed toward collecting further data from non-dosed livers.

Transcriptomics approaches can provide some further insight. Here, it was observed that inflammatory pathways are enriched prior to dosing and toward the end of perfusion as liver viability declines, suggesting that inflammation and liver damage is not solely a result of vector delivery in this system. Viral transcript pathways are enriched throughout but interferon pathways are enriched only at 12 and 72 h, which, with the spike in cytokines observed, suggests that an innate immune response is raised to the vector dose and possibly the expressed transgene later on, but not sufficiently to cause catastrophic inflammation and tissue damage.

It has been demonstrated that NMP livers can provide a great depth of data. In the case of LVs, this includes PK, transduction, integration, transgene expression profiles and transcriptome analysis. However, an organ in isolation presents clear limitations as a model, primarily due to the absence of additional organs and circulatory components. This limits the attainment of biodistribution data, for example, and so the transition away from animal models is still far from complete. The PK data are also unlikely to be truly representative of clearance following standard IV dosing at this stage: additional tissues and circulatory vessels undoubtedly would impact the amount of LV deposited in the liver from the bloodstream. The spleen, for instance, is instrumental in both filtration of pathogens from the bloodstream and initiation of innate and adaptive immune responses and could therefore have a major impact on vector PK and immune impact.^66^ In counter to this, it should be noted that, HA or PV infusion, although not ideal for ease of widespread clinical adoption, is becoming an accepted route for dosing with viral-based GT vectors. These routes and the rapid “first-pass” clearance they accentuate closely mimic the dosing performed in our *ex vivo* experiments.

The relatively short perfusion duration achieved is also of consequence. Despite robust methodology ensuring only integrated genomes were measured, we would expect that (in common with reported LV behavior in NHP) over time there would be silencing of expression through viral or transgene-directed immune responses,^16^ which are not detected within the limitations of this model. As perfusion technology and methodology improves, it is plausible that future studies may extend beyond two weeks and provide data concerning waning transgene expression and production of transgene-directed antibodies.

A further limitation of these studies is that the perfusate used was primarily composed of packed red blood cells, meaning complement, white blood cells, and free protein are largely absent. This may be addressed by utilization of whole blood or supplementing the current perfusate with individual components to establish the impact of each. Cabanes-Creus et al. achieved a similar effect by addition of AAV-neutralizing plasma to their perfusions.^21^ This ability to add and subtract each key blood component in order to quantify and stratify their impact and importance is a function that no other *in vivo* testing platform permits and certainly could not be achieved in a clinical trial.

The reproducibility of the system remains a major hurdle, stemming from variation in donor patient profile and liver condition, although it could be argued that this is more reflective of the ultimate intended patient population than the use of inbred strains of lab animals. Notably, despite marked difference in donor health and some differences in PK profile, the viral integration results observed in this study exhibit close similarity with no significant difference in total or integrated VCN between livers at the end of perfusion. During perfusion, triplicate biopsies were not always obtained. As these experiments were designed as a proof-of-concept exploration of what can be achieved in terms of sampling, dosing, and analysis, priority was given to providing a thorough examination of the time-series. Hence, the balance between optimizing sampling frequency, blood loss control/liver health, and replicate number was tipped toward the former. With the optimal sample timing now defined future studies can reduce sampling frequency, providing the opportunity to strengthen replicate number and the opportunity for statistical analysis*.* Some researchers have addressed access to livers and reproducibility by splitting livers, with one-half serving as a matched donor control for the other.^67^^,^^68^ In the United Kingdom, 1,099 whole livers were donated from deceased donors in the year 2023/24, with 25% of these (273) being rejected for transplant.^69^ This suggests a pool of around 250–300 livers available per year. The ability to make best use of this considerable and valuable resource will be reliant on access to perfusion devices and technical skills, which may prove limiting. Furthermore, ongoing improvements to perfusion systems aim to reduce the number of available organs that are deemed unsuitable for transplant.

These data are a proof-of-concept demonstration that helps define perfusion, sampling, and analysis procedures to enable whole human livers to be used for the profiling of LV PK, transduction, and integration. Although a small sample size, this preliminary study has demonstrated remarkable similarity between replicate perfusions of different age and health. This makes an important contribution to the argument that further optimization and ultimately adoption of this approach provides a means to reduce the unsustainable expense, time, and ethical cost associated with pre-clinical testing in rodents, pigs, and NHP. Such moves would be aligned with new regulatory guidance and a growing acceptance that the high attrition rates of therapeutic development can be addressed by moving away from animal models.

## Materials and methods

### Study design

Organs maintained by NMP present an attractive opportunity for preclinical testing of advanced therapies, potentially bridging the gap between animal models and human patients or replacing animal models entirely. In this study, four whole human livers rejected for transplant were maintained by NMP using a commercially available NMP device and dosed with 5.8 × 10^10^ TU or 1.16 × 10^10^ TU of VSV-G-pseudotyped LV containing a GFP reporter transgene under the control of a liver-specific ET promoter. Liver health was monitored by the device’s inbuilt sensors and biochemical analysis of the blood-based perfusate. Throughout perfusions, plasma and tissue loads of LV were determined by dPCR and GFP expression assessed by immunofluorescence at the end of perfusion.

### Ethical statement

The human livers used in this study were obtained from consenting organ donors and had been deemed unsuitable for transplant. Donor organs were accepted from NHSBT in accordance with a study plan approved by the NHS Health Research Authority (South West – Frenchay Research Ethics Committee, REC reference 20/SW/0133, study number ODT105). Human blood components used in the perfusate were sourced from NHSBT.

### Normothermic machine perfusion of whole human livers

Four whole human livers were perfused using a commercially available NMP device with FDA, European, Australian, and Canadian regulatory approvals for liver preservation prior to transplantation (metra, OrganOx Ltd, Oxford, UK). A 40 kDa hemoconcentrator (Medica S.p.A., Medolla, Italy) and two infusion pumps (Alaris SE, Becton, Dickinson and Company, Franklin Lakes, New Jersey, USA) were incorporated for control of perfusate volume and filtration.

Perfusion was started according to the manufacturers’ instructions for use. The device was primed according to protocol with 500 mL of Gelofusine colloidal volume replacement solution (B. Braun, Melsungen, Germany) and two units of packed human red blood cells (NC15, NHSBT, Bristol, UK), totaling approximately 1,200 mL of perfusate. After priming, boluses of 500 mg meropenem (Synchrony Pharma Ltd, Stevenage, UK) in 10 mL saline (Baxter International, Deerfield, Illinois, USA), 10,000 units of heparin (Wockhardt, Mumbai, India) in 10 mL saline, and 10 mL of 10% calcium gluconate (DEMO S.A. Pharmaceutical Industry, Athens, Greece) were administered. Infusions of sodium taurocholate (OrganOx Ltd), heparin, epoprostenol (GSK plc, Brentford, UK), and insulin (Novo Nordisk A/S, Bagsværd, Denmark) were continuously administered during perfusion, regulated by the device. Infusion medications were replaced, and the meropenem bolus was repeated every 24 h. Total parenteral nutrition was provided by attaching Nutriflex Special infusion solution (B. Braun) to the inbuilt nutrition pump.

Livers were received on ice, cannulated, and flushed with cold saline before connecting to the device via the HA, PV, inferior vena cava (IVC), and bile duct.

For the liver-free perfusion described in Figure S1 the NMP device was set up as described above with the same perfusate, boluses, and infusions, but in place of a liver a Y-connector attached the HA and PV tubing directly to the IVC.

### Liver function and hemodynamics

The NMP device recorded flow rates and pressures from the HA, PV, and IVC, and perfusate pH, pO2, pCO2, temperature, and bile production. Bile production is not included in this study as collection for LV quantification interrupted measurement of bile flow. Gas and biochemistry in the perfusate were measured using an i-STAT1 blood analyzer with CG4+ and CG8+ cartridges (Abbott, Green Oaks, Illinois, USA) and a Piccolo Xpress blood analyzer with Piccolo Comprehensive Metabolic Panel cartridge (Abaxis, Union City, California, USA). AST, ALT, and ALP were quantified by John Radcliffe Hospital clinical biochemistry laboratories. Glucose concentration was also measured with GlucoRx Nexus Blood Meter test strips (GlucoRx, Guildford, UK).

### Lentiviral vector

A non-replicative HIV-based LV encoding GFP under control of a liver-specific promoter was supplied by Oxford Biomedica (UK) Ltd. The vector was stored at −80°C until use.

### LV administration and plasma, bile, filtrate, and tissue sampling

5.8 × 10^10^ TU or 1.16 × 10^10^ TU of LV were administered as a 20 mL bolus via a two-way stopcock in the HA cannula over a period of approximately 10 s.

Five milliliters of perfusate was collected in BD Vacutainer Lithium Heparin blood collection tubes (Becton, Dickinson and Company) throughout perfusion. After 30–120 min of incubation at room temperature, the perfusate was centrifuged and the plasma fraction was collected and stored at −80°C. Every 12 h, 1 mL of bile and 1 mL of filtrate were collected and stored at −80°C. Tissue core biopsies were collected from the right lobe prior to onboarding and every 12 h during perfusion using a BioPince Ultra Full Core Biopsy Instrument (Argon Medical Devices, Plano, Texas, USA). Biopsies were stored in RNALater (Thermo Fisher Scientific, Waltham, Massachusetts, USA) at room temperature for 24 h before snap freezing with liquid nitrogen and storing at −80°C. After perfusion, ∼2 cm^3^ biopsies from each lobe were stored in RNALater or 10% neutral-buffered formalin. Tissue in RNALater was incubated at room temperature for 24 h, snap frozen with liquid nitrogen, and stored at −80°C. For L1, tissue was incubated in formalin at room temperature for 24 h before transfer to 30% sucrose and incubation at 4°C for 4 days. After sucrose incubation, tissue was transferred to plastic sample holder, coated in O.C.T. compound and snap frozen with liquid nitrogen. For L2-L4, after at least 24 h in formalin tissue was paraffin embedded.

### Quantification of LV RNA genomes in perfusate and bile

Perfusate, 0.424 mL, or 1 mL bile was centrifuged at full speed at 4°C for 30 min. The supernatant was discarded and the pellet resuspended in 140 μL DPBS (Gibco, Thermo Fisher Scientific). Subsequent RNA extraction and DNA removal were performed according to kit protocols (QIAamp Viral RNA Mini Kit 52904, QIAGEN, Hilden, Germany; DNA-free DNA Removal Kit AM1906, Thermo Fisher Scientific) with elution into 80 μL Buffer AVE. LV RNA genomes were quantified by reverse-transcription digital PCR (RT-dPCR) (QIAcuity One, QIAGEN) using the QIAcuity Probe PCR Kit (250101, QIAGEN) and a 26k 24-well nanoplate (250001, QIAGEN) with primers targeting the HIV packaging sequence (Table S12).

Vector half-life in perfusate was calculated using GraphPad Prism analysis tools.

### Quantification of active virus in perfusate

LV activity in plasma was determined using serial passaging.^36^ Twelve-well plates were seeded with 90,000 HEK293T cells per well in 1 mL growth medium (DMEM 10% fetal bovine serum 1% penicillin-streptomycin) and incubated at 37°C, 5% CO_2_. On the following day cells from two wells were removed with trypsin and counted. Media were removed from wells and replaced with 500 μL of 1:1 plasma:media mix and plates returned to incubation. About 3–6 h later wells were supplemented with an additional 1 mL growth medium. After 3 days incubation cells were passaged. A further two passages were performed, with the final transferring cells into 6-well plates. Once the wells of the final passage achieved confluency, cells were removed by trypsin, pelleted, resuspended in 200 μL DPBS and stored at −80°C until DNA extraction. DNA extraction of cell pellets was performed using PureLink Genomic DNA Mini Kit (K182002, Thermo Fisher Scientific) according to kit protocol. LV DNA genomes and the cellular gene *RPPH1* were quantified via dPCR. There are two copies of *RPPH1* per cell, so the total VCN per cell was calculated using Equation 1.

(Equation 1) $$\text{Vector}\;\text{Copy}\;\text{Number}\;\text{per}\;\text{Cell}=2\times \frac{\text{LV}\;\text{genome}\;\text{copies}}{\mathrm{RPPH}1\;\text{copies}}$$

The VCN/cell was then used in conjunction with the cell numbers at infection and infection volume to calculate the TU present in each plasma sample. Primer and probe sequences for the HIV packaging sequence and *RPPH1* are defined in supplementary materials Table S12.

### Quantification of LV DNA genomes in tissue

DNA extraction was performed using the DNeasy Blood and Tissue Kit (69504, QIAGEN). Tissue samples were weighed and normalized to 23 ± 1.5 mg. When <21.5 mg was available, all available tissue was used. Tissue was minced and incubated overnight with 180 μL of Buffer ATL (939011, QIAGEN) and 20 μL of proteinase K at 65°C with shaking at 750 rpm. DNA was then extracted according to kit protocol. LV DNA genomes and the cellular gene *RPPH1* were quantified via dPCR. VCN/cell was calculated using the formula previously described, whereby total VCN/cell is the total number of DNA LV genome copies (both integrated and non-integrated) per cellular genome and integrated VCN/cell is the number of DNA LV genome copies integrated into cellular genomes (per cellular genome) as quantified by the following method.

To quantify integrated VCN/cell, extracted DNA underwent gel electrophoresis size exclusion with a 15KB cut-off (PippinHT with 0.75% agarose high-pass cassette, Sage Science, Beverly, Massachusetts, USA) to isolate genomic DNA and exclude non-integrated LV genomes. VCN/cell was then quantified via dPCR.

### Tissue immunofluorescent imaging

For L1 cryopreserved samples, 10 μm sections were produced with a cryostat. Tissue sectioning of L2-L4 samples and staining and imaging of all sections were performed by the University of Oxford Cancer Translational Histopathology Laboratory. Four micrometer sections were produced from formalin-fixed paraffin-embedded (FFPE) samples. Sections were stained with DAPI, anti-GFP (ab183734, Abcam, Cambridge, UK), and anti-CD31 (M0823, Agilent, Santa Clara, California, USA). Anti-GFP was diluted 1/100 and incubated for 45 min, then antigen retrieval was performed with PH9 according to the Leica BOND retrieval protocol. Anti-CD31 was diluted 1/500 and incubated for 30 min, then antigen retrieval was performed with PH6 as per the Leica BOND retrieval protocol. Imaging was performed with PhenoImager HT (Akoya Biosciences, Marlborough, Massachusetts, USA).

### GFP spatial distribution analysis

Distribution of GFP across the liver lobule was assessed using the large images seen in Figure S6 in the image analysis software ImageJ.^70^^,^^71^ For each image, portal triads were identified, and rectangular regions of interest were selected between the PV and a nearby central vein, spanning the lobule axis. In the GFP channel, fluorescence intensity (gray values) was measured against distance along the axis from portal to central vein. Distance was normalized to a scale of 0 (PV) to 100 (central vein). This was repeated for multiple regions across the image, and gray values were averaged.

### Tissue RNA extraction and transcriptomics

Frozen tissue samples in RNALater were thawed at room temperature and weighed and transferred to fresh microcentrifuge tubes on ice. A limited number of replicates were used (0 h *n* = 2, 12 h *n* = 1, 24 h *n* = 2, 48 h *n* = 2, 72 h *n* = 2) to limit bleeding during perfusion and due to loss of 12 h replicate during sample processing. Total RNA extraction was performed using the PureLink RNA Mini Kit (12183018A Thermo Fisher Scientific) according to kit protocol, using microtube pestles and syringes for tissue lysis and homogenization. DNA removal was performed using the DNA-free DNA Removal Kit (AM1906 Thermo Fisher Scientific), and the eluted RNA was stored at −20°C.

Strand-specific mRNA sequencing was performed by Novogene (UK) Co. Ltd. Read pseudoalignment was performed with Kallisto^72^ using the *Homo sapiens* GRCh38.p14 genome assembly from Ensembl as a reference.^73^ Following alignment, transcripts were annotated using Bioconductor Ensembl based annotation package (EnsDb.Hsapiens.v86) in R.^74^ Following filtering of low counts and normalization, GSVA was performed using the Hallmark (H) and Reactome (C2:CP:REACTOME) curated gene sets.^40^^,^^75^ For presented data, Reactome gene sets were filtered for those containing the terms “HIV” and “Viral.”

### Statistical analysis

Descriptive statistics (mean and standard deviation) of continuous outcome measurements such as arterial flow rates or VCN/cell are presented in the text. Differences in total and integrated VCN/cell at endpoint were assessed by one-way ANOVA, and differences in total and integrated VCN/cell between lobes were assessed by one-way ANOVA followed by the Tukey multiple comparisons test using GraphPad Prism. Vector half-life in plasma was calculated using GraphPad Prism’s linear regression tool to fit two-phase exponential decay models, and GraphPad was also used to calculate the area under the curve. C_max_ and T_max_ were also calculated.

## Data and code availability

Data will be made available upon reasonable request.

## Acknowledgments

We thank Mandy Townsend from the Institute of Biomedical Engineering at the University of Oxford for her assistance with tissue sample preservation and sectioning.

We also thank the Oxford Cancer Translational Histopathology Laboratory for their assistance with sample sectioning, staining, and immunofluorescent imaging.

R.C.C. and C.C.C. are grateful for the generous benefaction of Mr. Donald Porteous who supports their research.

B.R.M.N. is supported by the 10.13039/501100000268BBSRC Studentship Advanced Bioscience of Viral Products, grant number BB/Y51343X/1.

The work was funded by a 10.13039/100014013UKRI 10.13039/501100000268BBSRC and Oxford Biomedica (OXB)-funded Advanced Bioscience of Viral Products (ABViP) Collaborative Training Partnership (CTP), grant number: BB/W009420/1. ABViP is a doctoral training program between Oxford Biomedica, 10.13039/501100000765University College London, and the 10.13039/501100000769University of Oxford and includes financial and in-kind support from the UK university partners.

## Author contributions

Conceptualization: C.C.C., K.A.M., R.C.C.; methodology: R.C.C., B.R.M.N., C.C.C., K.A.M., A.K., R.A.S.R.; investigation: B.R.M.N., D.J., A.K., R.A.S.R.; visualization: R.C.C., D.J., C.C.C., K.A.M.; funding acquisition: C.C.C., R.C.C., K.A.M.; project administration: R.C.C., K.A.M., A.K., C.C.C.; supervision: C.C.C., R.C.C.; writing – original draft: B.R.M.N., R.C.C.; writing – review & editing: B.R.M.N., R.C.C., C.C.C., K.A.M., A.K., R.A.S.R.

## Declaration of interests

The work was funded by Oxford Biomedica (UK) Ltd. K.A.M., A.K., and R.A.S.R. are employees of Oxford Biomedica (UK) Ltd and received compensation in the form of salary and stock options.

In addition to his academic role as chair of biomedical engineering, C.C.C. is also a founder, director, and shareholder and receives consultancy income from OrganOx Ltd.

## Supplemental information

Document S1. Figures S1–S6 and Tables S1–S12

Document S2. Article plus supplemental information
