# Supplementary material for: Normothermic perfusion of human livers for profiling lentiviral vector pharmacokinetics and transduction
Source: Mol Ther Adv. 2025 Dec 26;34(1):201660. doi: 10.1016/j.omta.2025.201660 (PMC13182791; doi:10.1016/j.omta.2025.201660)
Supplement: Document S1. Figures S1–S6 and Tables S1–S12 [file mmc1.pdf]

OMTA, Volume *34*

Supplemental information

Normothermic perfusion of human livers for profiling lentiviral vector pharmacokinetics and transduction

Brannon R.M. Nicholls, David Johnson, Anurag Kulkarni, Rui André Saraiva Raposo, Kyriacos A. Mitrophanous, Constantin C. Coussios, and Robert C. Carlisle

Supplemental materials

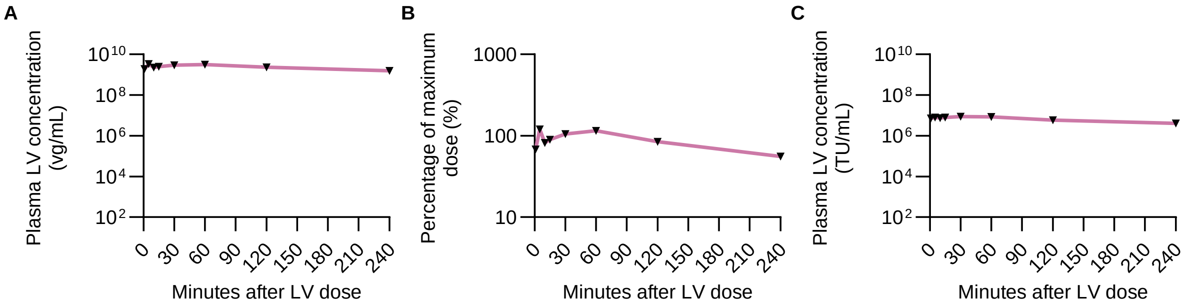

Figure S1. **Pharmacokinetics of LV administered to a liver-free perfusions (A)** LV genome (vg) concentration in plasma fraction of perfusate following administration of 5.8 × 10^{10} TU to liver-free perfusion system, first time point at 1 minute after dose. **(B)** LV genome concentration in perfusate as a percentage of the theoretical maximum concentration, calculated from measured LV concentration relative to input LV genomes using a perfusate volume of 1200 mL and assuming homogenous distribution of LV in the perfusate. **(C)** LV infectious particle concentration (transducing units (TU)/mL) in perfusate as quantified by *in vitro* infectivity assay.

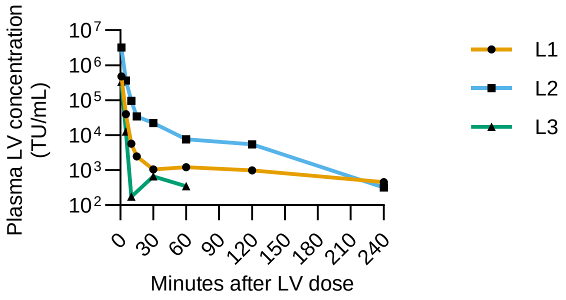

Figure S2. **Clearance of active LV in L1, L2 and L3 plasma.** LV transducing unit concentration (transducing units (TU)/mL) in plasma as quantified by *in vitro* infectivity assay, using plasma collected following administration of 5.8×10^{10} TU to perfused livers L1, L2 and L3.

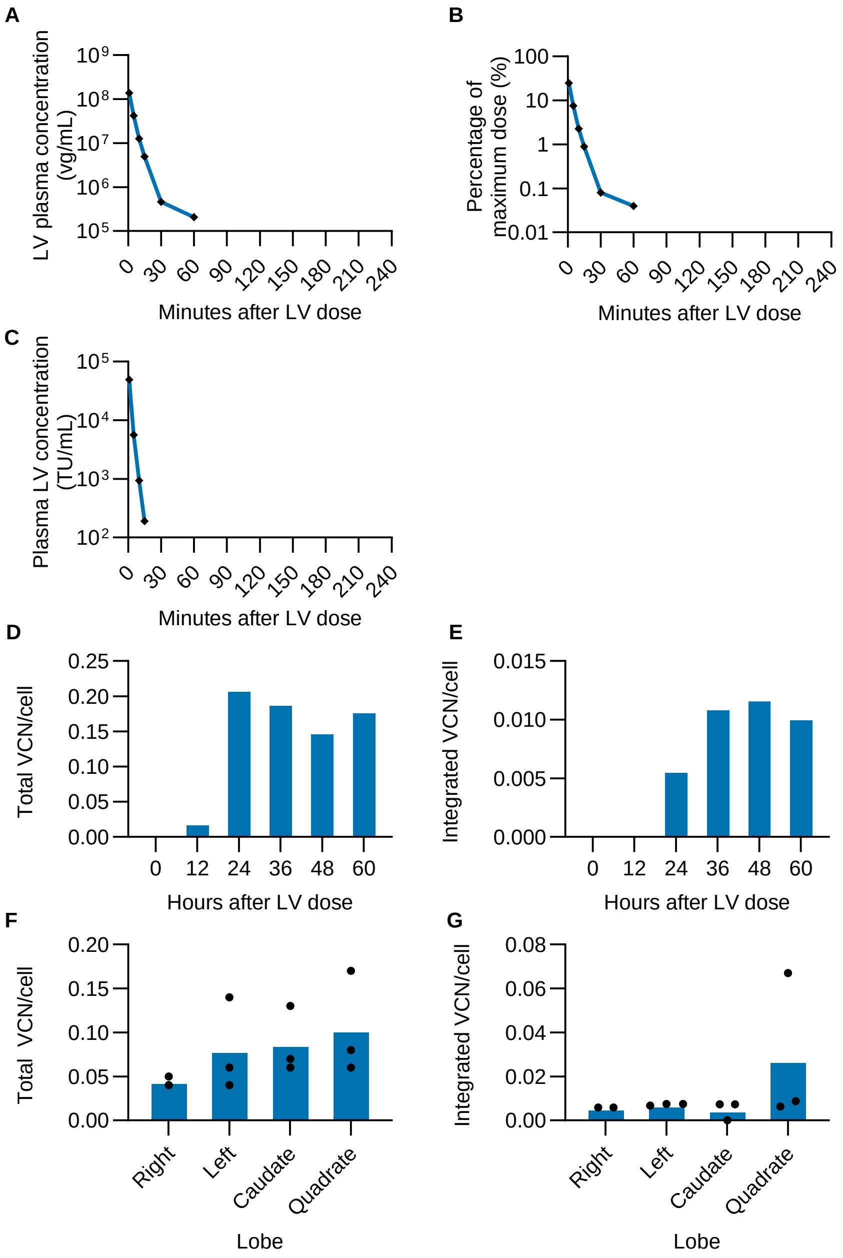

Fig. S3. Pharmacokinetics, integration and distribution of LV administered to perfused liver L4. (A) LV genome (vg) concentration in plasma fraction of perfusate following administration of 1.16×10^{10} TU LV to perfused liver L4, first time point at 1 minute after dose. **(B)** LV genome concentration in perfusate as a percentage of the theoretical maximum concentration, calculated from measured LV concentration relative

to input LV genomes using a perfusate volume of 1200 mL and assuming homogenous distribution of LV in the perfusate. **(C)** LV infectious particle concentration (transducing units (TU)/mL) in perfusate as quantified by *in vitro* infectivity assay. **(D)** Total VCN/cell and **(E)** integrated VCN/cell of liver tissue taken from the right lobe of L4 every 12 hours via core biopsies. **(F)** Total VCN/cell and **(G) i**ntegrated VCN/cell of tissue from L4 lobes at the end of perfusion. For **(F)** and **(G),** black circles indicate biopsy technical replicates and bars represent the mean.

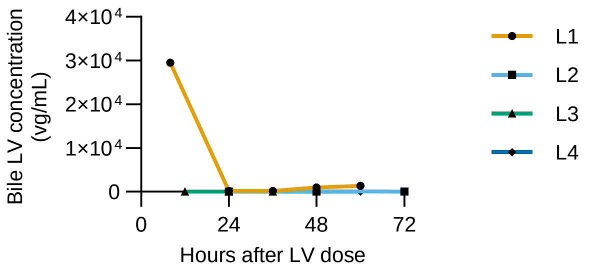

Fig. S4. Vector concentration in bile of perfused livers. LV concentration in bile produced by livers L1, L2, L3 and L4 was quantified by RT-dPCR.

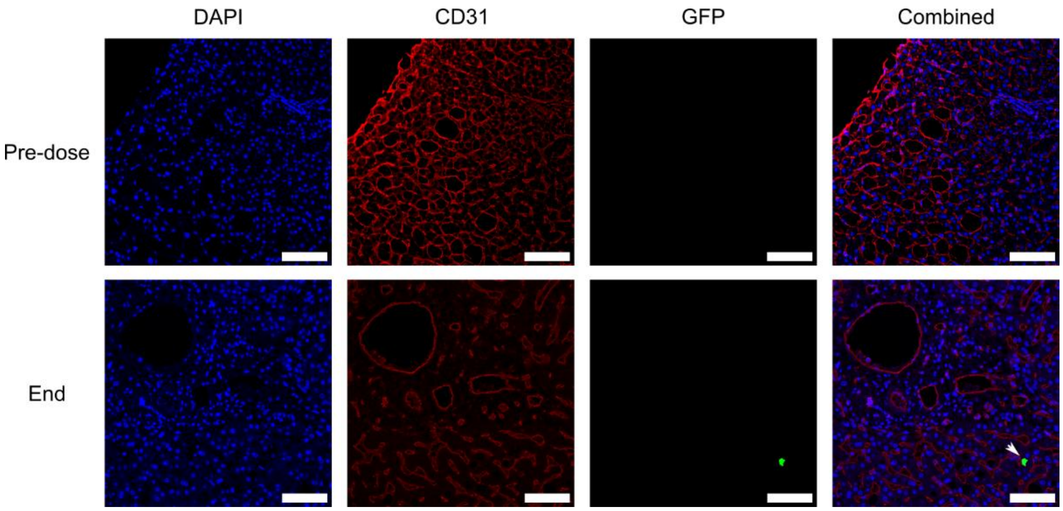

Fig S5. **Liver 4 histology.** Biopsies from L4 before LV dose (Pre-dose) and at the end of perfusion (End) were taken and preserved by FFPE. 4 µm sections were stained with DAPI (blue) and antibodies against CD31 (red) and GFP (green). Scale bar 50 µm. White arrow indicates GFP expressing cell.

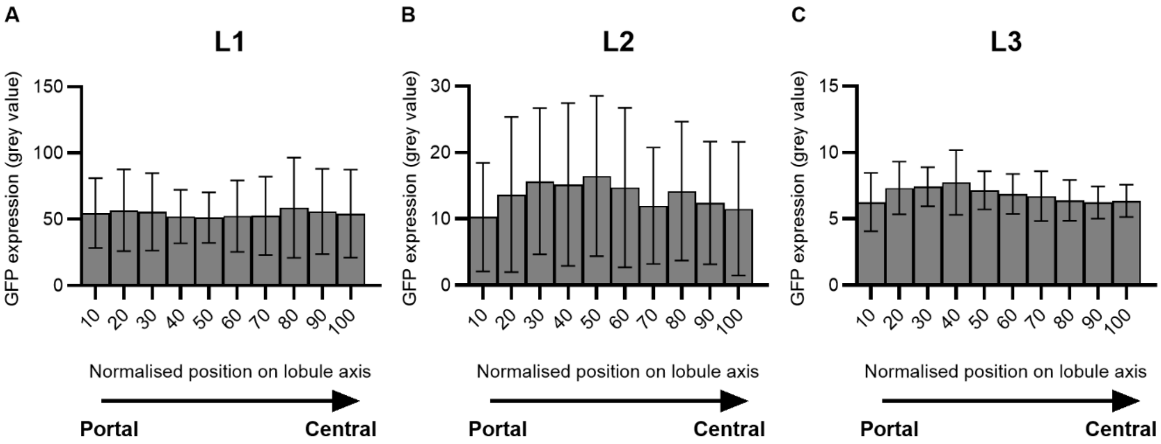

Figure S6. Spatial deposition of GFP expression. GFP expression (grey value) in immunofluorescent images was measured across the lobule axis from portal vein to central vein for livers L1-L3. Multiple axes were measured for each liver and distance normalised to a 0-100 scale, 0 being adjacent to the portal vein and 100 adjacent to the

central vein. Bars represent average grey value in the GFP channel and error bars standard deviation.

Table S1. Pharmacokinetic parameters of L1, L2, L3 and L4. Pharmacokinetic parameters of vector clearance from perfusate were calculated for L1-L4.

Liver	C_{max} (vg/mL)	T_{max} (minutes)	Slow half-life (minutes)	Fast half-life (minutes)	AUC (vg·hours/mL)
L1	9.09×10^8	1	13.20	1.42	5.05×10^7
L2	9.58×10^8	1	13.99	1.31	4.94×10^7
L3	6.44×10^7	1	1.25	1.25	2.80×10^6
L4	1.37×10^8	1	4.95	1.91	9.84×10^6

Table S2. One-way analysis of variance of liver lobe total VCN/cell at endpoint of L1, L2, L3 and L4.

ANOVA table	SS	DF	MS	F (DFn, DFd)	P value
**L1**					
Treatment (between columns)	1.305	3	0.4351	F (3, 7) = 7.874	P=0.0121
Residual (within columns)	0.3869	7	0.05526		
Total	1.692	10			
**L2**					
Treatment (between columns)	0.03420	3	0.01140	F (3, 8) = 0.3923	P=0.7620
Residual (within columns)	0.2325	8	0.02906		
Total	0.2667	11			
**L3**					
Treatment (between columns)	1.393	3	0.4644	F (3, 8) = 52.37	P<0.0001
Residual (within columns)	0.07093	8	0.008867		
Total	1.464	11			
**L4**					
Treatment (between columns)	0.004180	3	0.001393	F (3, 7) = 0.6341	P=0.6163
Residual (within columns)	0.01538	7	0.002198		
Total	0.01956	10			

Table S3. Tukey's multiple comparisons test comparing total VCN/cell of each lobe at endpoint of L1, L2, L3 and L4.

Tukey's multiple comparisons test	Mean diff.	95.00% CI of diff.	Below threshold?	Summary	Adjusted P Value
**L1**					
Right vs. Left	-0.4817	-1.192 to 0.2287	No	ns	0.2009
Right vs. Caudate	-0.07167	-0.7820 to 0.6387	No	ns	0.9861
Right vs. Quadrate	0.4483	-0.2620 to 1.159	No	ns	0.2446
Left vs. Caudate	0.4100	-0.2254 to 1.045	No	ns	0.2306
Left vs. Quadrate	0.9300	0.2946 to 1.565	Yes	**	0.0078
Caudate vs. Quadrate	0.5200	-0.1154 to 1.155	No	ns	0.1097
**L2**					
Right vs. Left	0.01667	-0.4290 to 0.4624	No	ns	0.9993
Right vs. Caudate	-0.07000	-0.5157 to 0.3757	No	ns	0.9561
Right vs. Quadrate	0.08000	-0.3657 to 0.5257	No	ns	0.9369
Left vs. Caudate	-0.08667	-0.5324 to 0.3590	No	ns	0.9219
Left vs. Quadrate	0.06333	-0.3824 to 0.5090	No	ns	0.9668
Caudate vs. Quadrate	0.1500	-0.2957 to 0.5957	No	ns	0.7117
**L3**					
Right vs. Left	0.7933	0.5471 to 1.040	Yes	****	<0.0001
Right vs. Caudate	0.4067	0.1605 to 0.6529	Yes	**	0.0033
Right vs. Quadrate	0.8467	0.6005 to 1.093	Yes	****	<0.0001
Left vs. Caudate	-0.3867	-0.6329 to -0.1405	Yes	**	0.0045
Left vs. Quadrate	0.05333	-0.1929 to 0.2995	No	ns	0.8967
Caudate vs. Quadrate	0.4400	0.1938 to 0.6862	Yes	**	0.0020

L4

Right vs. Left	-0.03500	-0.1767 to 0.1067	No	ns	0.8444
Right vs. Caudate	-0.04167	-0.1833 to 0.09999	No	ns	0.7683
Right vs. Quadrate	-0.05833	-0.2000 to 0.08332	No	ns	0.5567
Left vs. Caudate	-0.006667	-0.1334 to 0.1200	No	ns	0.9979
Left vs. Quadrate	-0.02333	-0.1500 to 0.1034	No	ns	0.9258
Caudate vs. Quadrate	-0.01667	-0.1434 to 0.1100	No	ns	0.9704

Table S4. One-way analysis of variance of liver lobe integrated VCN/cell at endpoint of L1, L2, L3 and L4.

ANOVA table	SS	DF	MS	F (DFn, DFd)	P value
**L1**					
Treatment (between columns)	0.03046	3	0.01015	F (3, 7) = 4.376	P=0.0493
Residual (within columns)	0.01624	7	0.002320		
Total	0.04669	10			
**L2**					
Treatment (between columns)	0.0008978	3	0.0002993	F (3, 8) = 0.6926	P=0.5819
Residual (within columns)	0.003457	8	0.0004321		
Total	0.004354	11			
**L3**					
Treatment (between columns)	0.03727	3	0.01242	F (3, 8) = 8.146	P=0.0082
Residual (within columns)	0.01220	8	0.001525		
Total	0.04947	11			
**L4**					
Treatment (between columns)	0.0009998	3	0.0003333	F (3, 7) = 0.9737	P=0.4573
Residual (within columns)	0.002396	7	0.0003423		
Total	0.003396	10			

Table S5. Tukey's multiple comparisons test comparing integrated VCN/cell of each lobe at endpoint of L1, L2, L3 and L4.

Tukey's multiple comparisons test	Mean diff.	95.00% CI of diff.	Below threshold?	Summary	Adjusted P Value
**L1**					
Right vs. Left	-0.1333	-0.2789 to 0.01221	No	ns	0.0717
Right vs. Caudate	-0.06667	-0.2122 to 0.07887	No	ns	0.4771
Right vs. Quadrate	-0.01153	-0.1571 to 0.1340	No	ns	0.9931
Left vs. Caudate	0.06667	-0.06351 to 0.1968	No	ns	0.3923
Left vs. Quadrate	0.1218	-0.008375 to 0.2520	No	ns	0.0659
Caudate vs. Quadrate	0.05513	-0.07504 to 0.1853	No	ns	0.5360
**L2**					
Right vs. Left	0.01860	-0.03576 to 0.07295	No	ns	0.7019
Right vs. Caudate	0.009388	-0.04496 to 0.06374	No	ns	0.9431
Right vs. Quadrate	0.02231	-0.03204 to 0.07666	No	ns	0.5798
Left vs. Caudate	-0.009207	-0.06356 to 0.04514	No	ns	0.9460
Left vs. Quadrate	0.003712	-0.05064 to 0.05806	No	ns	0.9960
Caudate vs. Quadrate	0.01292	-0.04143 to 0.06727	No	ns	0.8695
**L3**					
Right vs. Left	0.1433	0.04123 to 0.2454	Yes	**	0.0087
Right vs. Caudate	0.07667	-0.02544 to 0.1788	No	ns	0.1534
Right vs. Quadrate	0.1267	0.02456 to 0.2288	Yes	*	0.0173
Left vs. Caudate	-0.06667	-0.1688 to 0.03544	No	ns	0.2345
Left vs. Quadrate	-0.01667	-0.1188 to 0.08544	No	ns	0.9512

Caudate vs. Quadrate	0.05000	-0.05211 to 0.1521	No	ns	0.4456
**L4**					
Right vs. Left	-0.001282	-0.05719 to 0.05462	No	ns	0.9998
Right vs. Caudate	0.001052	-0.05485 to 0.05696	No	ns	>0.9999
Right vs. Quadrate	-0.02141	-0.07731 to 0.03450	No	ns	0.6087
Left vs. Caudate	0.002333	-0.04767 to 0.05234	No	ns	0.9986
Left vs. Quadrate	-0.02012	-0.07013 to 0.02988	No	ns	0.5734
Caudate vs. Quadrate	-0.02246	-0.07246 to 0.02755	No	ns	0.4920

Table S6. Descriptive statistics of total VCN/cell at endpoint of L1, L2 and L3.

Liver	1	2	3
Mean	0.6213	0.5767	0.6750
Std. Deviation	0.3810	0.06164	0.3934

Table S7. One-way analysis of variance of mean total VCN/cell at endpoint of L1, L2 and L3.

ANOVA table	SS	DF	MS	F (DFn, DFd)	P value
Treatment (between columns)	0.01939	2	0.009697	F (2, 9) = 0.09579	P=0.9096
Residual (within columns)	0.9112	9	0.1012		
Total	0.9306	11			

Table S8. Descriptive statistics of integrated VCN/cell at endpoint of L1, L2 and L3.

Liver	1	2	3
Mean	0.1129	0.07792	0.1333
Std. Deviation	0.06102	0.009988	0.06435

Table S9. One-way analysis of variance of mean integrated VCN/cell at endpoint of L1, L2 and L3.

ANOVA table	SS	DF	MS	F (DFn, DFd)	P value
Treatment (between columns)	0.006281	2	0.003140	F (2, 9) = 1.183	P=0.3498
Residual (within columns)	0.02389	9	0.002654		
Total	0.03017	11			

Table S10. Estimated GFP expression in immunofluorescently stained FFPE sections. Total and GFP positive cell counts in 1.3 x 1.3 mm regions of fluorescently stained sections from L1, L2 and L3. No percentage listed for L4: very few GFP cells counted across the entire 1cm^2 section in Fig. S2D.

Liver	Total cells counted	GFP cells counted	Percent GFP expression
L1	8023	50	0.62
L2	4082	165	4.04
L3	3006	10	0.33

Table S11. Comparison of ALT and AST half-life to published values. Half-life of ALT and AST was calculated for L1-L4 and compared to values published by Kim et al.[63]

Liver	ALT T1/2 (hours)	AST T1/2 (hours)
L1	54	108
L2	50	57
L3	N/A	N/A
L4	35	46
Kim et al. 2008	47 +/- 10	17 +/-5

Table S12. Primer sequences for digital PCR quantification of viral RNA and DNA genomes.

Target	Forward primer sequence	Reverse primer sequence	Probe sequence
HIV packaging signal	5’ TGGGCAAGCAGGGAGCTA 3’	5’ TCCTGTCTGAAGGGATGGTTG T 3’	5’ FAM-AACGATTCGCAGTTAATCCTG GCCTGTT-TAMRA 3’
RPPH1	5’ CCCTAGTCTCAGACCTTCCCA AG 3’	5’ GCGGAGGGAAGCTCATCAG 3’	5’ VIC-CCACGAGCTGAGTGCGTCCT GTCA-TAMRA 3’
