# Supplementary material for: Normothermic perfusion of human livers for profiling lentiviral vector pharmacokinetics and transduction
Source: Mol Ther Adv. 2025 Dec 26;34(1):201660. doi: 10.1016/j.omta.2025.201660 (PMC13182791; doi:10.1016/j.omta.2025.201660)
Supplement: Document S2. Article plus supplemental information [file mmc2.pdf]

Molecular Therapy
Advances
Original Article

Normothermic perfusion of human livers for profiling lentiviral vector pharmacokinetics and transduction

Brannon R.M. Nicholls,[1] David Johnson,[1] Anurag Kulkarni,[2] Rui André Saraiva Raposo,[2] Kyriacos A. Mitrophanous,[2] Constantin C. Coussios,[1] and Robert C. Carlisle[1]

[1]Institute of Biomedical Engineering, Department of Engineering Science, University of Oxford, Botnar 3 Research Building, Windmill Road, Headington, Oxford OX3 7LD, UK; [2]Oxford Biomedica (UK) Ltd, Windrush Court, Transport Way, Oxford OX4 6LT, UK

Lentiviral vectors (LVs) hold significant potential for gene therapy (GT) due to their ability to integrate into non-dividing cells, potentially offering lifelong cures from a single dose. The liver is an attractive GT target due to its role in inherited disorders and as a sink for intravenously delivered therapeutics. However, clinical translation of LV therapy remains challenging due to the poor predictive value of animal models. Normothermic machine perfusion (NMP) maintains human organs under physiological conditions *ex vivo*, creating an opportunity to reduce reliance on animal studies and de-risk human clinical trials. We used NMP to assess pharmacokinetics and transduction in four human livers dosed with an LV encoding green fluorescent protein (GFP). Perfusion was maintained for up to 74 h, achieving physiological viability and function. Rapid LV clearance was observed, with less than 1% remaining in perfusate after 10 min. Integrated viral copy number per cell reached 0.07–0.13, with detectable GFP expression. Transcriptomic analysis revealed dynamic changes in metabolic and inflammatory pathways correlating with liver function and transduction outcomes. This study demonstrates that NMP provides a useful model to assess LV delivery and transduction, supporting its potential as a platform to enhance translation into human clinical applications.

INTRODUCTION

Lentiviral vectors (LVs) are of particular interest for use in gene therapies (GTs) due to their ability to transduce quiescent cells and integrate transgenes into the target cell's genome, promoting long-term therapeutic expression and potentially offering a lifelong cure for genetic disorders.[1] LVs also possess sufficient capacity for multiple genes to be delivered, and a low rate of patient preexposure reduces the likelihood of pre-existing antibody-based immunity to LVs,[2] an issue with many adeno-associated virus (AAV) vectors.[3–5] However, neutralization in human serum still occurs through complement responses to common LV pseudotypes and allogeneic responses to producer cell membrane proteins such as major histocompatibility complex.[6–8] Such responses have contributed to a lack of development of LV GT vectors for *in vivo* use following intravenous (IV) delivery.

Although LVs are used extensively for *ex vivo* GT, the clinical landscape *in vivo* remains poor: while there are nine *ex vivo* LV GTs approved by the US Food and Drug Administration (FDA) or European Medicines Agency (EMA), there are none approved for IV delivery.[9,10] There are signs of improvement: three phase I clinical trials using IV LV delivery for *in vivo* generation of CAR-T cells are underway,[11] but the scientific and clinical progress made with *ex vivo* delivery has yet to be matched.

The liver is a popular target for *in vivo* GT as it is the site of many genetic disorders such as hemophilia and alpha-1 antitrypsin deficiency.[12] Addressing such diseases with LV is dependent on limited neutralization in blood, evasion of liver resident macrophages, Kupffer cells (KCs), and efficient transduction and integration into the genome of hepatocytes. Notably, the liver is the major clearance site for bloodborne particles and can remove and inactivate >90% of circulating virus following IV delivery.[13] Understanding LV delivery and capture in human livers is therefore a prerequisite for developing GTs with both hepatic and extra-hepatic targets. Test models of appropriate scale and anatomy for this are lacking, despite studies in large animal models including pigs and non-human primates (NHPs).[14–16] A review by Baruteau et al. highlighted a number of examples where animal studies of AAV liver-directed GTs failed to predict outcomes in human patients,[17] and such findings are likely to also be observed for LV. Without accurate recapitulation of human anatomy and physiology there is a risk that safety and efficacy evidence gathered in mice and NHP models will be as misleading for LV as it is for other classes of drug: just 10% of new pharmaceuticals that successfully navigate preclinical studies ultimately gain approval for human use.[18] Notably, 50% of this unsustainable attrition rate is due to a lack of efficacy, highlighting an industry-wide need for alternative preclinical models.[19]

Received 7 July 2025; accepted 18 December 2025;
https://doi.org/10.1016/j.omta.2025.201660.

Correspondence: Robert C. Carlisle, Institute of Biomedical Engineering, Department of Engineering Science, University of Oxford, Botnar 3 Research Building, Windmill Road, Headington, Oxford OX3 7LD, UK.
E-mail: robert.carlisle@eng.ox.ac.uk

Published by Elsevier Inc. on behalf of The American Society of Gene and Cell Therapy.
This is an open access article under the CC BY license (http://creativecommons.org/licenses/by/4.0/).

Table 1. Description of perfused human livers

Liver	Patient death	Reason declined for transplant	Condition notes	Weight (kg)	Patient age (years)	Patient sex (M/F)	Total ischemia time (HH:MM)	Total perfusion duration (HH:MM)	Perfusion duration after dose (HH:MM)	Reason for perfusion end
L1	DBD	Fatty	Round edges, mildly steatotic	2.2	74	M	13:14	70:56	68:52	Excessive bleeding post biopsy
L2	DCD	Function on NRP	AST increased during normothermic regional perfusion	Not recorded	58	M	12:22	74:03	71:13	Increasing lactate
L3	DBD	Anatomy	Longitudinal hematoma, arterial thrombosis, mildly fibrotic	1.614	78	F	13:09	55:22	51:22	Increasing lactate
L4	DCD	Untransplantable	Calcification of hepatic artery, low-moderately fatty, underperfused segment VII	2.18	66	M	13:44	66:40	64:34	Increasing lactate

Four whole human livers were maintained by normothermic machine perfusion following rejection for transplant. DBD: donation after brainstem death, the patient has been declared dead based on neurological criteria but circulation continues. DCD: donation after circulatory death, the patient has been declared dead after cessation of circulatory and respiratory function. DCD organs may be exposed to longer warm ischemia times, increasing the risk of organ damage. NRP: normothermic regional perfusion, whereby circulation and oxygenation of an organ is restored *in situ* using extracorporeal assistance.

One potential alternative to animal models is the use of normothermic machine perfusion (NMP) to maintain human organs under physiological conditions *ex vivo*. Commercially available NMP devices have been approved for clinical use and demonstrated to increase preservation time, reduce graft injury, and improve patient outcomes for higher-risk livers when compared with static cold storage (SCS).[20] Recent studies have used NMP to study the delivery and liver cell distribution of AAVs and identify serotypes with preferential infection of hepatocytes.[21,22] To date, no studies have used the approach to profile LV pharmacokinetics (PK) and pharmacodynamics (PD).

In this study, we administered a reporter-encoding LV to human donor livers maintained by NMP and profiled clearance from circulation, viral entry and reverse transcription, integration, and transgene expression. Furthermore, we obtained temporally discrete data from tissue biopsies throughout the perfusion following a sampling regimen, which would not be possible or permissible in animal models or patients. In this manner, we provide insights into the effective delivery of LV to human organs while adding to the growing body of evidence supporting use of NMP organs as the most relevant and effective preclinical models.

RESULTS

Liver function maintained for up to 70 h following LV administration

Four whole human livers classified as suitable for donation at point of resection (L1, L2, L3, and L4) but subsequently deemed unsuitable for transplant (Table 1) were obtained from National Health Service Blood and Transplant (NHSBT) and connected to the NMP device as described in the methods (Figure 1).

For NMP to provide a useful model of liver LV capture and transduction the perfused liver must meet normal human health and function parameters. The NMP device provides a range of outputs enabling liver function to be tracked and controlled, which can be complemented by blood gas and chemistry measurements using separate blood analyzers.

After establishment of hemodynamics and blood chemistry in the normal physiological range (achieved in under four hours for all livers), each liver was dosed with GFP-expressing LV and perfusion maintained for up to 74 h (Table 1). Mergantal et al. proposed that one of two major criteria (bile production or lactate concentration below 2.5 mmol/L) and two or more minor criteria (blood pH > 7.3, glucose metabolism, hepatic arterial flow >150 mL/min, portal vein (PV) flow >500 mL/min, or homogeneous perfusion) must be met by donor livers receiving NMP to predict successful function in transplant recipients,[23] and these criteria were used to assess liver viability in this study (as indicated by the dotted lines in Figure 2).

As shown in Figures 2A–2C, all livers achieved flow rates in or close to the physiological range for the majority of perfusion. Average arterial flow was 0.47, 0.49, 0.63, and 0.28 L/min (SD 0.12, 0.10, 0.14, 0.10) for L1-L4, respectively, and average portal flow was 1.04, 1.13, 0.97, and 1.23 L/min (SD 0.19, 0.09, 0.40, 0.24), well above the thresholds proposed by Mergental et al.[23] and in line with normal *in vivo* flow rates (normal adult total hepatic blood flow is between 1.5 and 1.9 L/min, two-thirds of which is supplied by the hepatic PV and the remainder by the hepatic artery [HA]).[24] Normal flow for L3 was temporarily interrupted between 31 and 39 h after perfusion start due to an infusion pump fault, which altered perfusate volume and pressure, with no PV flow received during that period. Normal flow rates were resumed; however, this malfunction likely contributed to the premature decline of L3.

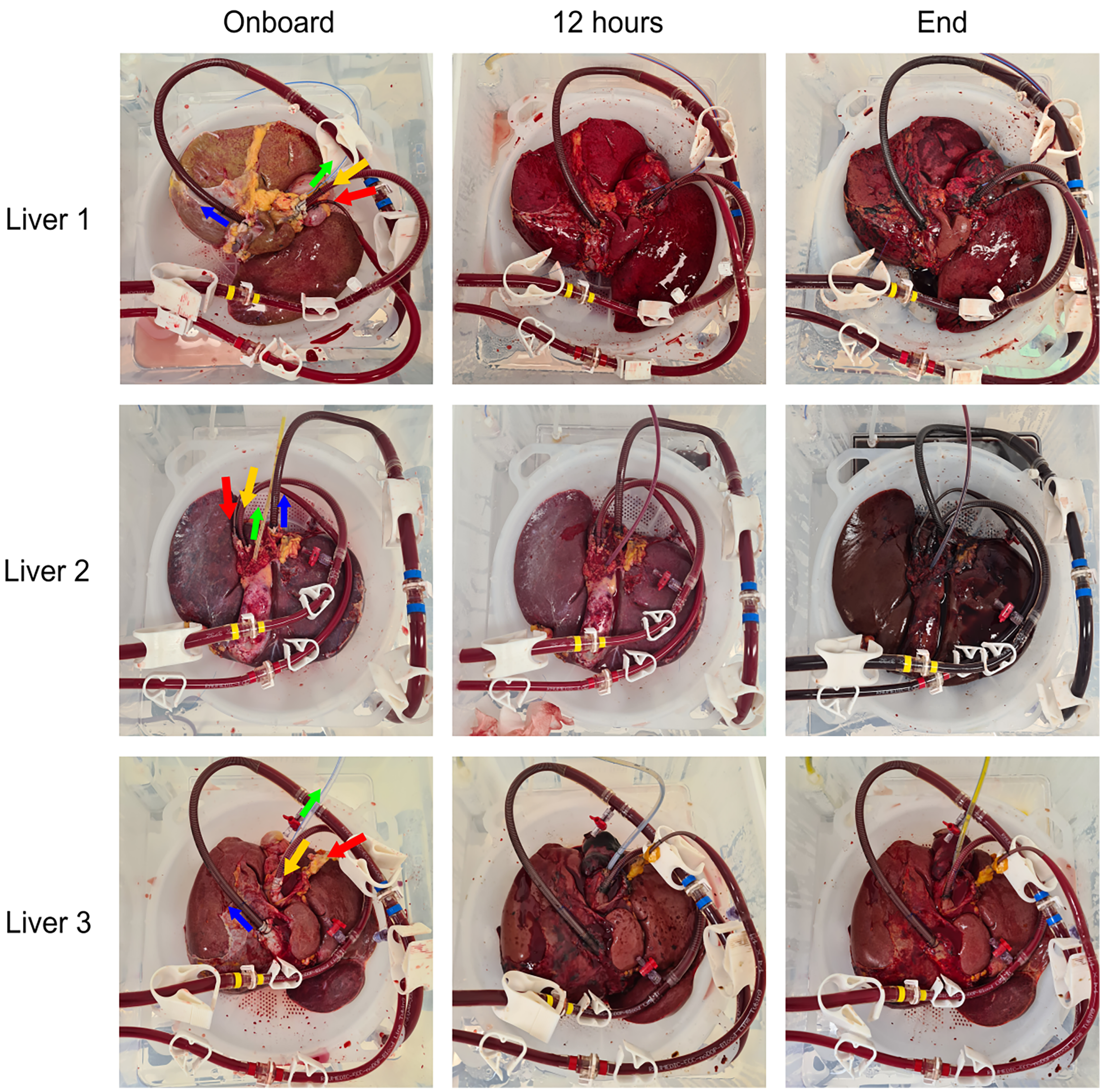

Figure 1. Normothermic machine perfusion of whole human livers

Four whole human livers were dosed with LV and maintained by NMP for up to 74 h. Top-down view of livers connected to the NMP device throughout perfusion. Arrows indicate vessel tubing and flow direction: red, hepatic artery; yellow, hepatic portal vein; blue, inferior vena cava; green, bile duct. Following perfusion livers change from pale/blue to a deep red signifying the successful re-establishment of flow and health.

Normal blood pH is between 7.35 and 7.45. Perfusate was acidotic for L1, L2, and L4 at the start of perfusion, correcting to the normal range in under 6 h, while L3 began at pH 7.45 (Figure 2D). L3 pH dropped severely within the period of interrupted perfusate flow but was rapidly returned to the normal range upon the resumption of normal flow rates.

Lactate clearance has been established as a reliable indicator of liver function.[25] Perfusate lactate concentration for all four livers remained below 2.5 mmol/L, the suggested threshold value, for the first 24 h after perfusion start (Figure 2E). By 39 h, L3 lactate had dramatically risen to nearly 20 mmol/L, almost certainly a result of the interrupted flow rates previously described. It soon decreased below 10 mmol/L; however, after 54 h the concentration continued to increase: this strongly suggests a suboptimally functioning liver beyond 15 h, the last time point when lactate was falling and was below 2.5 mmol/L. In contrast, L1 and L2 lactate only increased beyond 2.5 mmol/L after 60 h and L4 after 50 h, indicating normal metabolic activity throughout the majority of perfusion.

The enzymes alkaline phosphatase (ALP), alanine aminotransferase (ALT), and aspartate aminotransferase (AST) are serum biomarkers of liver damage and have commonly been used as surrogate endpoints for long-term graft survival in clinical studies of liver

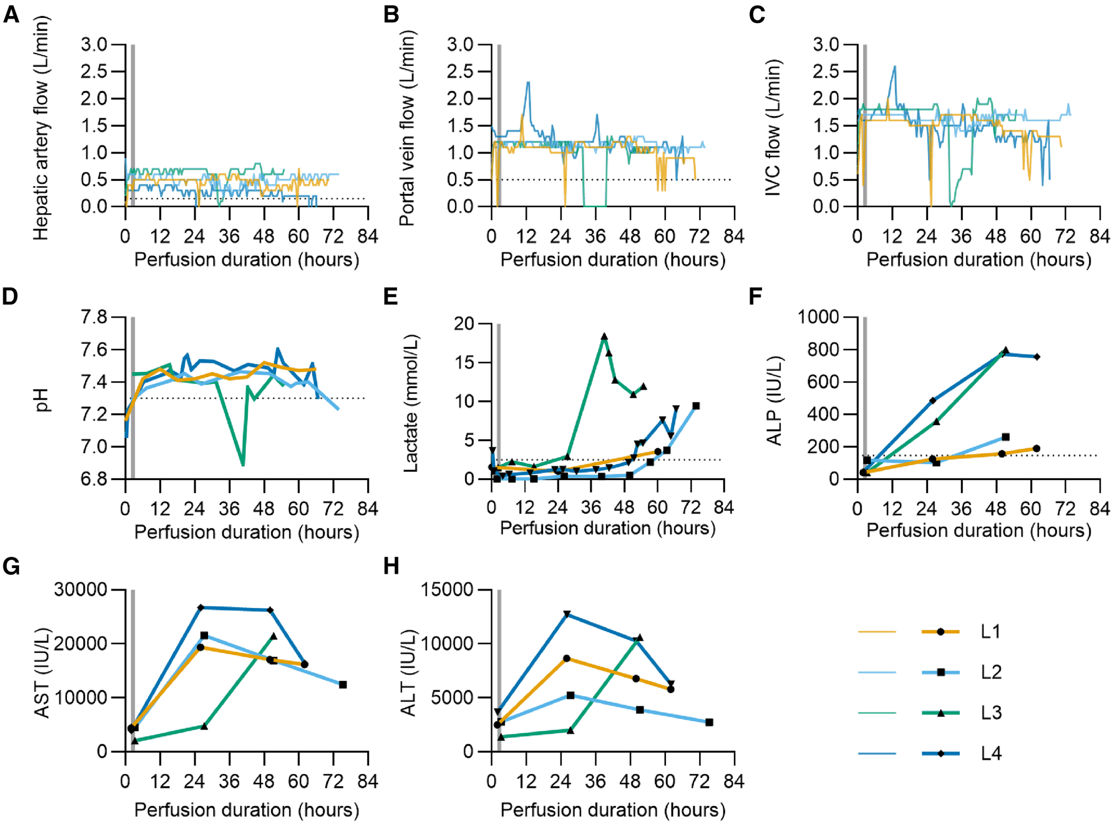

Figure 2. Metrics of liver function. Four discarded whole human livers ("L1" to "L4") were maintained by normothermic machine perfusion and dosed with lentiviral vector

(A) Hepatic artery blood flow. (B) Portal vein blood flow. (C) Inferior vena cava (IVC) blood flow. (D) Perfusate pH. (E) Perfusate lactate concentration. (F) Perfusate alkaline phosphatase (ALP) concentration. (G) Perfusate aspartate aminotransferase (AST) concentration. (H) Perfusate alanine aminotransferase (ALT) concentration. Dotted lines in (A), (B), and (D) indicate minimum values, and dotted line in (E) indicates maximum value required to meet viability criteria described by Mergental et al.[23] Dotted line in (F) indicates the maximum normal range for patient ALP. Normal ranges for AST and ALT not shown due to scale of y axis.

transplantation.[26,27] For all livers, ALP was within the healthy range (<147 IU/L) prior to dosing but increased beyond that by 50 h (Figure 2F).[28] ALP was particularly high at approximately 800 IU/L for L3 and L4 at 50 h, whereas the increase was more gradual for L1 and L2. Prior to dosing, all three livers displayed levels of AST and ALT above the normal patient range[29,30] but in line with expectations after 2–3 h of reperfusion (Figures 2G and 2H).[31–34] The substantially raised levels observed in samples taken at 24 h post-LV dosing may represent damage resulting from LV infection or delayed reperfusion injury responses as ALT and AST levels greater than 1,000 IU/L can be indicative of severe liver damage from a number of causes such as ischemia, drug-induced damage, or viral hepatitis.[35]

All perfusions were maintained beyond 48 h, extending to 74 h for L2. L1 perfusion was ended due to excessive post-biopsy bleeding, and suturing of biopsy wounds was adopted to limit this in the following two perfusions. Perfusion of L2, L3, and L4 was ended due to increasing lactate concentrations. Overall, physiological flow rates combined with pH maintenance and lactate clearance suggest normal conditions were maintained throughout the majority of perfusion, and liver damage biomarkers, although high, did show signs of recovery.

LV is rapidly removed from circulation and achieves replicable transduction in NMP human livers

Following normalization of blood flow and biochemistry, L1-L3 were dosed with 5.8×10^{10} transducing units (TU) (3.34×10^{12} RNA vector genomes, vg) LV with GFP transgene under control of a hepatocyte-specific promoter in a 20 mL bolus to the HA. This dosing was based on a previous experiment performed on isolated porcine-derived liver (Clark et al. under review). The fourth liver "L4" received a 5-fold lower dose to investigate linearity of PK and PD outputs and the potential for dose sparing. The PK was defined in terms of both vector RNA copy number and transducing vector concentration circulating in plasma. RNA copy number was quantified by reverse-transcriptase digital PCR (RT-dPCR) of serially collected plasma samples (Figures 3A and 3B), while the transducing titer was determined by application of plasma samples to *in vitro* cell

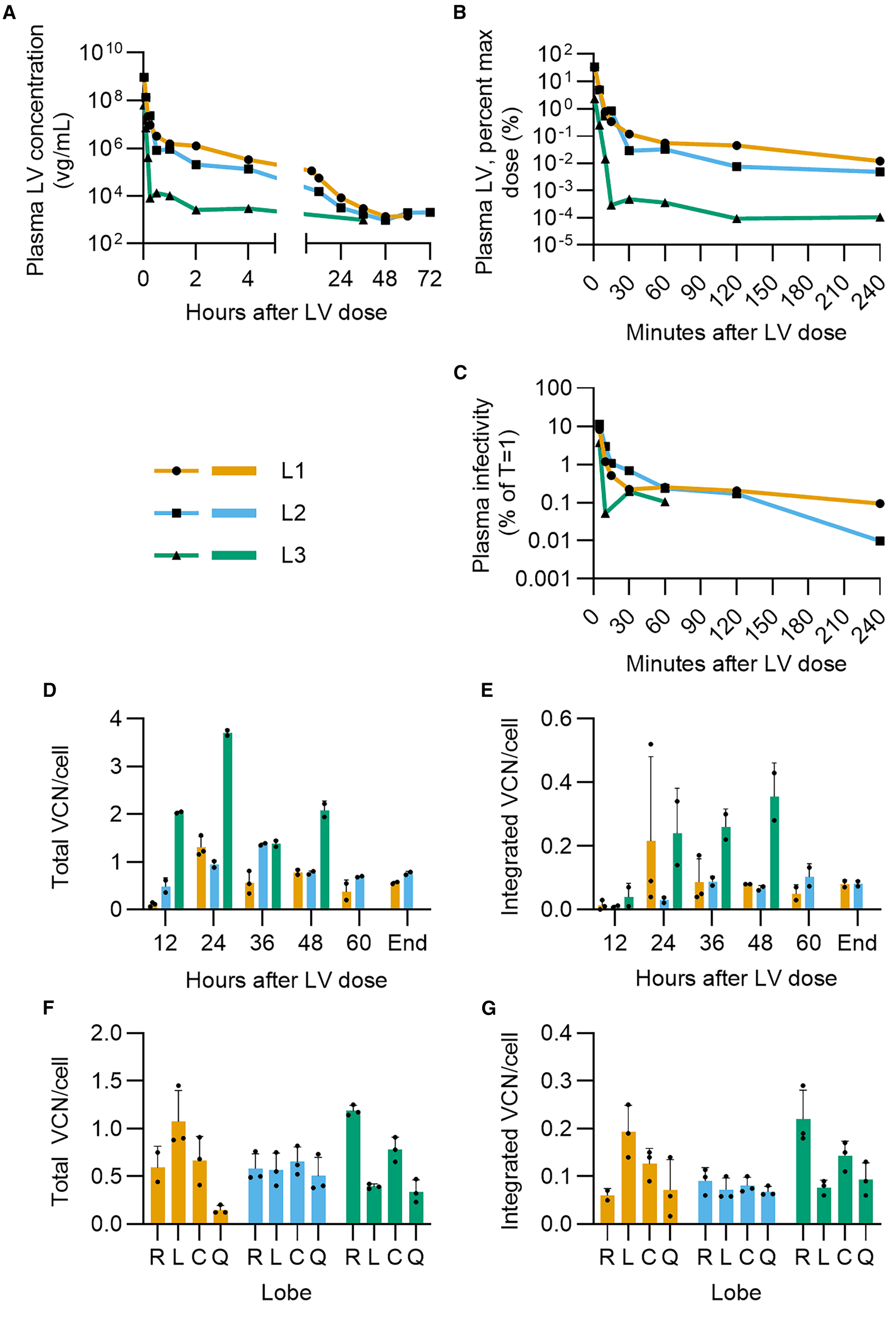

(legend on next page)

monolayers and subsequent quantification of integrated LV genomes by serial passaging and dPCR (Figure 3C), a method adapted from that used by Moore-Kelly et al.[36]

The theoretical maximum dose, presuming homogenous dissolution of the whole dose within the total perfusate volume, was calculated by division of the total input RNA vector genomes by the total perfusate volume. Following administration, LV genomes were rapidly cleared from L1, L2, and L3, with plasma concentration dropping more than 100-fold in the first 10 min to below 1% of the maximum dose (Figure 3B). By 4 h, less than 0.1% of the maximum dose was in circulation. Clearance for L1 and L2 is biphasic, with an initial fast half-life of 1.42 and 1.32 min (Table S1). One-phase decay followed by a plateau appears a better fit for L3, with a half-life of 1.26 min. Following this the clearance rate slowed, and RNA genomes continued to circulate at extremely low levels for the remainder of all perfusions. Application of the same dose to the perfusion device without a liver connected established that there is minimal LV loss to the system when a liver is not present (Figure S1), suggesting that the rapid decline in LV genome concentration observed is in fact a result of uptake by the liver. Transduction of cell monolayers *in vitro* was used to validate the RT-dPCR data and also probe the loss or retention of activity in the circulating LV dose (Figures 3C and S2). Clearance of actively transducing LV (TU/mL) closely reflects the observed changes in vg/mL.

In L4, dosing at one-fifth of the dose used in L1-3 led to no LV genomes being detectable beyond 60 min (Figure S3), indicating some saturation of a clearance mechanism may be achieved at the higher dose. LV genomes were detected in L1 bile at 8 h after dosing but had greatly reduced by 24 h. For L2-L4, little or no LV was detected in bile throughout perfusion (Figure S4).

Having defined clearance from the circulation, total vector copy number (VCN)/cell and integrated VCN/cell were quantified from tissue samples taken from the right lobe of each liver every 12 h after dosing. Total VCN includes both integrated and non-integrated vector DNA copies in cells, while integrated VCN refers to only those vector DNA copies that have inserted into the host cell genome. All livers exhibited a peak in total VCN/cell, between 24 and 36 h, before subsequently declining (Figure 3D). L1 and L2 peaked at 1.3 and 1.4 VCN/cell before declining to 0.6 and 0.8 by the end of perfusion, while L3 had a greater peak of 3.7 VCN/cell at 24 h, which subsequently declined to 2.1 by 48 h. It may be that the more rapid plasma clearance observed for L3 resulted in greater delivery to tissues and increased infection.

Integrated VCN/cell was established by quantification of vector genomes in isolated genomic DNA as described in the methods (Figure 3E).[37] Integrated VCN/cell peaked at 24 h for L1, reaching 0.2 before stabilizing until the end of perfusion. L2 did not exhibit such a peak but increased until 36 h and then remained level at 0.08–0.09 until the end of perfusion, whereas L3 VCN/cell continued to increase to 0.36 by 48 h after which no further samples were available.

At the end of perfusion, VCN/cell was calculated from samples across all lobes (Figures 3F and 3G). VCN/cell varied across lobes, but integrated VCN/cell was only significantly different between the right and left and right and quadrate lobes of L3 (Tables S2, S3, S4, and S5). Average total VCN/cell at endpoint was very similar between L1 and L3 at 0.621 (SD 0.381) for L1, 0.577 (SD 0.062) for L2, and 0.675 (SD 0.393) for L3, showing no significant difference between perfusions despite the more rapid clearance rate observed in L3 perfusate (Tables S6 and S7). Mean integrated VCN/cell also showed no significant difference at 0.113, 0.078, and 0.133 for L1, L2, and L3, respectively (SD 0.061, 0.010, 0.064) (Tables S8 and S9), equivalent to transduction of 11%, 8%, and 13% of cells. L4 demonstrated similar transduction trends to L1-L3 but achieved lower VCN/cell at endpoint of 0.057 (total) and 0.007 (integrated), 5–10% of the VCN achieved by L1-L3 and in line with the lower dose received (Figures S3D–S3G).

Cytokine profiles reveal pre-existing inflammation variability

Analysis of cytokine levels may provide information on background health status of donated organs, levels of surgical and perfusion injury, and/or the impact of LV dosing. Inflammatory cytokines in filtrate collected from the hemoconcentrator were quantified via a multiplex cassette system as described in the methods (Figure 4). For most livers, all cytokines exhibited a peak at 4 h after dosing, suggesting a strong immune response resulting from vector administration. L2 and L3 interleukin-1 beta (IL-1β) increased 100-fold within 4 h of dosing, from 25 pg/mL and 3 pg/mL to 2,955 pg/mL and 748 pg/mL, respectively. L1 peaked at 13,150 pg/mL at 4 h, and all three declined below time 0 concentration by 48 h. In contrast, IL-1β concentration of L4 gradually increased throughout, from <1 pg/mL at time 0 to 32 pg/mL by 60 h. This could reflect the lower dose received by L4.

L2, L3, and L4 interleukin-6 (IL-6) all peak at greater than 300,000 pg/mL at 4 h after dosing before declining approximately 100-fold

Figure 3. Pharmacokinetics, integration, and distribution of LV administered to NMP livers

(A) LV genome (vg) concentration in plasma fraction of perfusate following administration of 5.8×10^{10} TU (3.34×10^{12} vg) to perfused human livers, first time point at 1 min after dose. (B) LV genome concentration in perfusate as a percentage of the theoretical maximum concentration, calculated from measured LV concentration relative to input LV genomes using a perfusate volume of 1,200 mL and assuming homogenous distribution of LV in the perfusate. (C) Infectivity of plasma as a percentage of T = 1 transducing concentration (TU/mL). Plasma TU/mL was determined by *in vitro* infectivity assay and is presented as a percentage of T = 1 to account for different sample treatment, which may have affected measured infectivity (L1 plasma was subjected to an additional round of freeze-thaw after storage). (D) Total VCN/cell (integrated and non-integrated) and (E) integrated VCN/cell of liver tissue taken from the right lobe of each liver every 12 h via core biopsies. (F) Total VCN/cell and (G) integrated VCN/cell of tissue from each liver lobe at the end of perfusion. For (D) to (G), black circles indicate biopsy technical replicates, bars represent the mean, error bars indicate standard deviation. For (F) and (G), R, L, C, and Q represent the Right, Left, Caudate, and Quadrate lobes, respectively, the four lobes of the human liver.

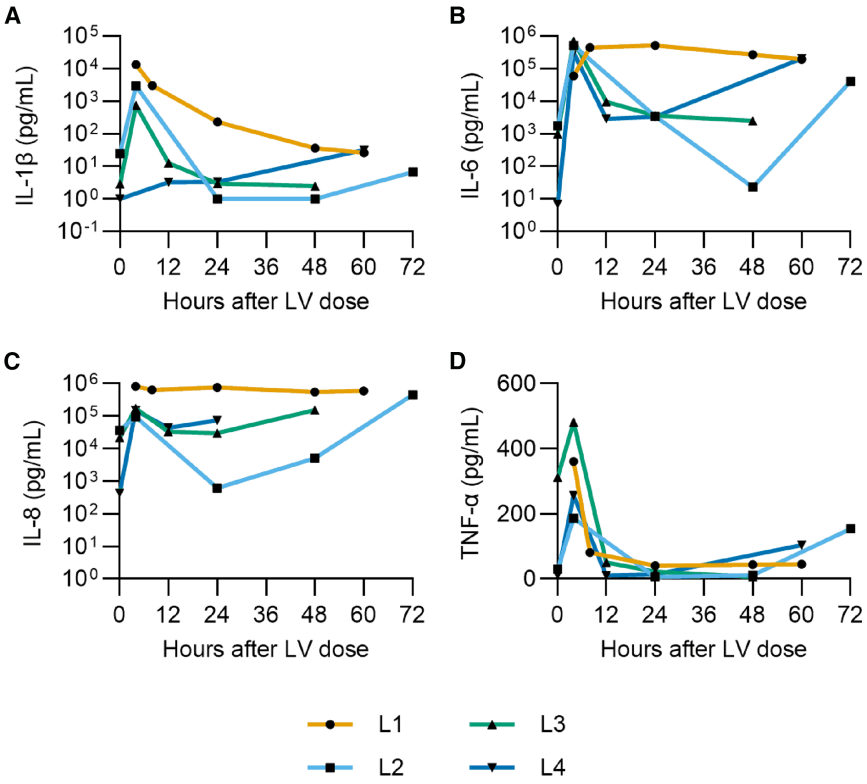

Figure 4. Cytokine profiles of NMP livers dosed with LV

Filtrate was collected from the hemoconcentrator throughout each perfusion and cytokine concentration quantified with a multiplex assay cassette. (A) IL-1β, (B) IL-6, (C) IL-8, (D) TNF-α. LV dose was given at time 0; time 0 samples were collected immediately prior to dosing.

by 24 h, again suggesting activation of an anti-viral response. By the end of perfusion L2 and L4 IL-6 increased. This may be due to liver condition rather than in response to LV dose, as IL-6 is strongly associated with liver damage response pathways with both pro- and anti-inflammatory functions.[38] Interleukin-8 (IL-8) follows a similar pattern, with a peak at 4 h for L2-4 and a gradual increase from 24 h onwards.

Similar to the other cytokines, tumor necrosis factor alpha (TNF-α) peaked at 4 h and then decreased for all livers up to 48 h, increasing again for L2 and L4 beyond 60 h, which could reflect declining viability. The initial high concentration of TNF-α for L3 may reflect a more inflamed liver condition, and it is possible that higher inflammation may have increased leakiness of L3 vasculature and contributed to the faster clearance of vector from plasma previously described.

GFP expression detectable in perfused livers by 51 h

Sections from the right lobes of L1-L3 were fluorescently stained for GFP to assess the level and distribution of GFP transgene expression (Figure 5). The sections were also stained for the epithelial marker CD31 to highlight the complex vascular structure of the liver.

GFP expression was detected across all livers, confirming successful transduction of tissue in all perfusions (Figures 5 and S5). Expression was most extensive in L2 with GFP detectable in approximately 4% of L2 cells but in fewer than 1% of L1 and L3 cells despite L2 having the lowest and L3 the highest integrated VCN/cell (Table S10). This may be partly explained by the extended perfusion duration achieved by L2 providing time for increased transcription and translation of the GFP transgene. The difference in L2 integration and expression (7% vs. 4%) suggests that not all transduced cells are expressing GFP at a sufficient level to be detectable. This is expected as the vector is pseudotyped with a broad tropism vesicular stomatitis virus G protein (VSV-G) envelope and the transgene is under control of a hepatocyte-specific promoter, although, interestingly, L3 exhibited some GFP expression in CD31-positive cells.

Analysis of the spatial deposition of GFP expression within liver lobes was performed as described in the methods. The data shown in Figure S6 demonstrate that expression is achieved in equivalent levels for the entire span between the portal triad and the central vein. This is an interesting finding running contrary to other studies of nanoparticle distribution in the liver lobule,[39] which may be due to differences in the test species used (i.e., rat) or the "hardness" of the materials used.

Gene set variation coincides with observed liver function and inflammation

RNA extraction and mRNA sequencing of tissue samples from L2 was performed (Figures 6 and 7). Gene set enrichment analysis was performed using the Molecular Signatures Database (MSigDB) Hallmark collection[40–42] to identify pathway expression changes throughout perfusion relative to a tissue sample taken immediately prior to dosing with LV.

At time 0, metabolic pathways including those associated with oxidative phosphorylation and fatty acid metabolism were upregulated, likely indicating an increase in metabolism in response to the cessation of SCS and the initiation of NMP. At 72 h, these pathways were inhibited and those associated with hypoxia and glycolysis were upregulated, coinciding with increased lactate levels and denoting a change in the metabolic profile of cells at later time points. Between 48 and 72 h pathways associated with cell-cycle regulation and differentiation (e.g., G2M checkpoint and epithelial-mesenchymal transition) were upregulated, suggesting a process of repair and tissue restructuring had started.

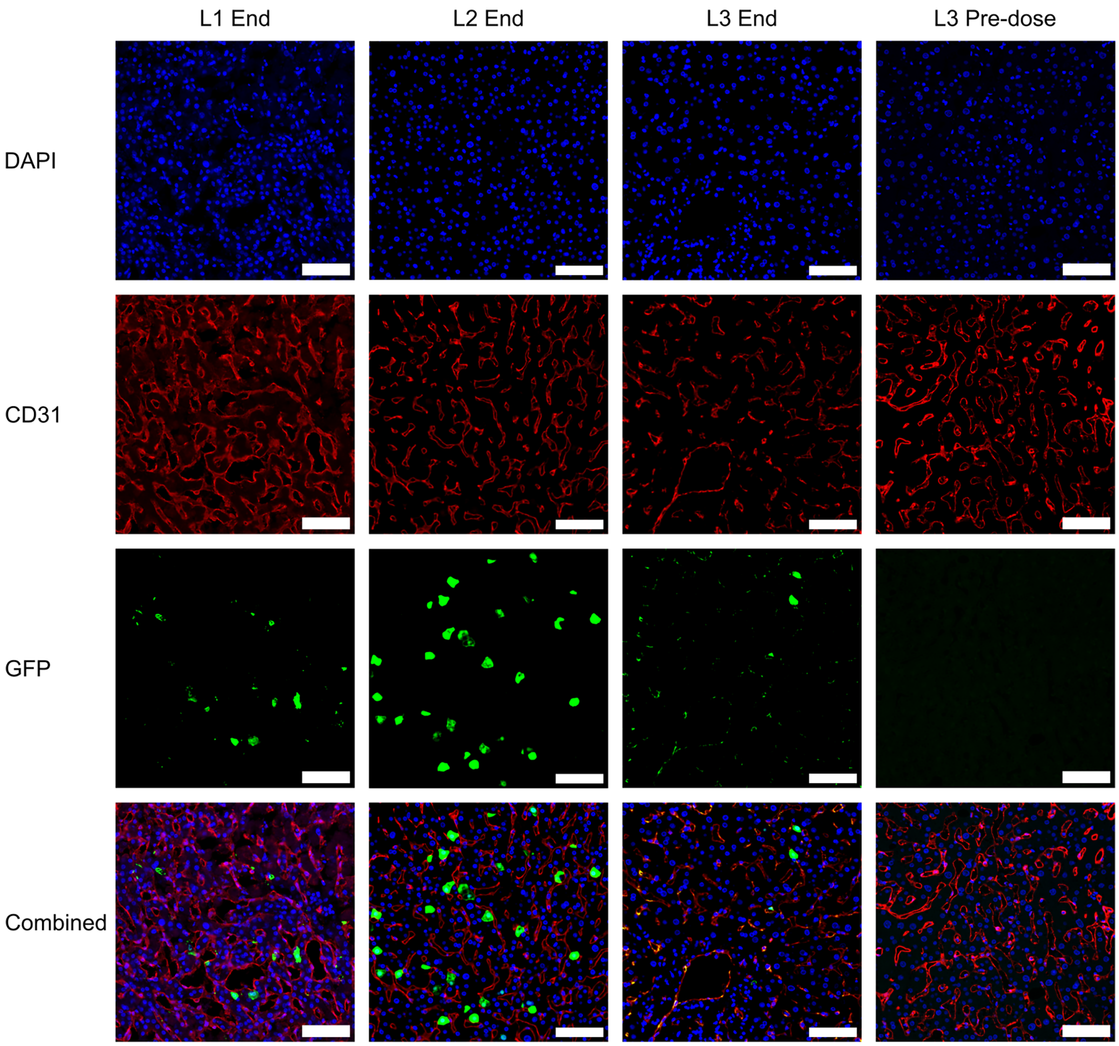

Figure 5. GFP expression detectable in NMP livers dosed with LV

GFP expression in tissue of L1, L2, and L3. Following perfusion tissue segments from L1 were cryopreserved in OCT compound and 10 μm sections produced. Segments from L2 and L3 were preserved by FFPE and 4 μm sections produced. All sections were stained with DAPI (blue) and antibodies against GFP (green) and the epithelial marker CD31 (red). L3 Pre-dose biopsy taken during perfusion shortly before dosing with LV. Scale bars, 50 μm.

Inflammatory response pathways were activated prior to dosing and at the end of perfusion, probably a reflection of the liver's inflamed state following harvest and transport and an increase in inflammation as its condition deteriorated toward the end of perfusion. In particular, IL-6 and TNF-α signaling corresponds with observed changes in cytokine concentration. The decreasing oxidative metabolism and increased inflammation pathways combined with the increased lactate and inflammatory cytokines observed support the notion that liver health was in decline by 72 h.

Interferon responses demonstrated a modest activation at 12 h relative to time 0, followed by strong activation at 72 h. Initial activation at 12 h was likely in response to LV delivery. The resurgence at 72 h may be in response to production of the LV transgenes in transduced cells, or a result of an increasingly inflamed environment.

Gene set variation analysis (GSVA) was also performed with the MSigDB Reactome collection, which details 1,736 gene sets versus the 50 contained in the Hallmark collection and so should enable a more granular analysis of signaling pathways. GSVA results were filtered for terms pertaining to HIV and viral infection response (Figure 7). As expected, nearly all HIV-associated pathways were inactive prior to dosing. At 12 h after LV dosing there was activation of both viral infection and antiviral pathways such as interferon and APOBEC3G antiviral responses. These are reflective of both active transduction of cells and the innate immune response raised against the vector.

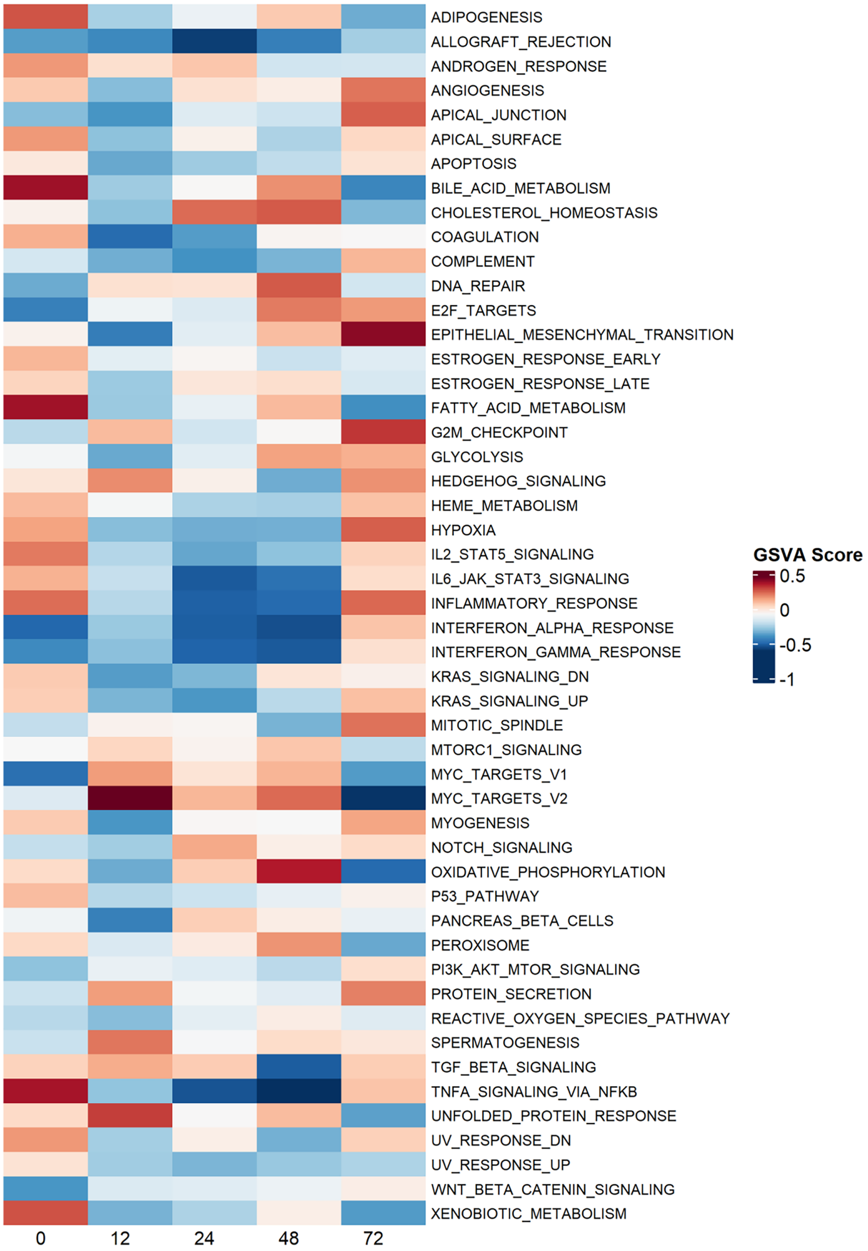

Figure 6. Gene set variation analysis of Hallmark pathways in L2

Total mRNA from L2 tissue samples was isolated and sequenced and gene set variation analysis performed using MSigDB Hallmark gene sets. Tissue samples were taken at 0 (pre-dose), 12, 24, 48, and 72 h after dosing with LV.

Between 12 and 48 h there was an increase in provirus integration pathways, corresponding to the increase in integrated VCN/cell observed. By 72 h most viral life cycle pathways were down-regulated and antiviral responses were again increased, coinciding with the interferon response identified with the Hallmark gene sets.

DISCUSSION

NMP is now a well-established procedure that can maintain liver viability between donation and transplant and improve outcomes for patients receiving from higher-risk DCD (donation after circulatory death) donors.[20,43,44] Recently, there has been increasing interest in use of perfused organs as pre-clinical models as the only way to truly recapitulate the anatomy and function of a human patient.[21,22] Indeed, the approach allows a level of sampling and analysis that is not practically or ethically feasible in clinical trials, an important feature when profiling the PK and PD of advanced biologic therapies. The growing availability of commercial perfusion systems also makes NMP livers an increasingly available research tool.

Lentiviral GTs have great promise for long-term treatment of liver resident and extra-hepatic inherited and acquired disorders, but IV delivery of LV to patients is still in its infancy. In this study, we demonstrate that NMP livers are capable of profiling the PK and transduction of a liver-directed lentiviral GT vector, thereby demonstrating the utility of the system and also gaining insights into LV delivery and activity.

Four human livers were maintained by NMP for up to 74 h. Defined against the limits suggested by Mergental et al.,[23] three of the four livers were functionally viable for the majority of perfusion duration, and perfusion was ended following deviation from these limits. The duration achieved did not match the 13 days reported by Lau et al.[45] but was sufficient to observe LV transduction and integration events, as well as transgene expression (as evidenced by the stabilization of integrated VCN/cell after 48 h) and GFP fluorescence.

Following dosing, rapid clearance from the perfusate was observed. Perfusate LV genome concentration declined over 100-fold within 10 min, faster clearance than that observed with an AAV vector in a similar, albeit open, perfusion system, which experienced a decline to 9% of the initial vector concentration after 24 h[21] Through binding the low-density lipoprotein receptor, VSV-G confers the LV with a broad tropism that likely contributes to its rapid removal from the NMP system.[46] This tropism includes KCs, which, combined with

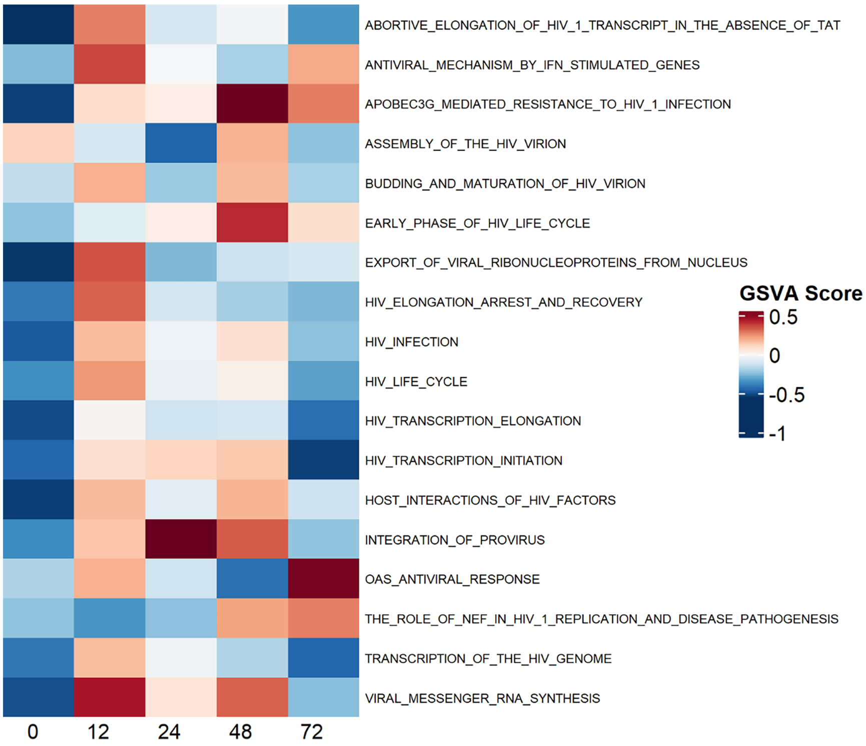

Figure 7. Gene set variation analysis of viral Reactome pathways in L2

Total mRNA from L2 tissue samples was isolated and sequenced and gene set variation analysis performed using MSigDB Reactome gene sets. Presented data were filtered for terms "HIV" and "viral." Tissue samples were taken at 0 (pre-dose), 12, 24, 48, and 72 h after dosing with LV.

their phagocytic ability, makes them primarily responsible for LV clearance and limiting hepatocyte transduction.[47] High levels of transduction by LV are well documented in human macrophages *in vitro* and in mouse KCs following IV delivery.[16,48,49] Lower KC transduction has been reported in primates, although this is typically after dosing with "stealthy" immune-evading vectors,[16,49] and it remains to be seen how much KC transduction is achieved in human patients or in a whole human liver setting. Observed clearance also surpassed reported rates for LV in some animal models. Delivery of 2×10^9 TU/kg of HIV-derived vector to NHPs resulted in a half-life of 1 h,[50] much longer than the sub-2 min rates observed here. This discrepancy may relate to differing total doses leading to saturation of clearance mechanisms in the NHP experiments, which may not have been achieved here; however, the total dose in the NHP study was not specified. In addition, the first NHP time point was taken at 30 min after dosing, so substantial initial vector clearance may have been missed. Alternatively, it may relate to physiological differences in the livers of the two species. We have already observed that LV experiments in perfused porcine livers (Clark et al., under review) gave extended circulation compared with the results reported here. Porcine liver has been reported to have a smaller fenestrated endothelial gap size (82 nm) vs. human (107 nm), which could impede hepatocyte access in the porcine model and comparatively reduce clearance.[51] Similar effects may be expected in the NHP model (gap size reported as 77–82 nm in baboons), but regardless it is clear that our studies raise concerns about the scientific validity of using large animal models to profile liver clearance of all viral and non-viral nanoscale therapeutics.

The NMP system enabled the regular retrieval of tissue biopsies throughout perfusion, a feat not easily attainable in animal models or human clinical trials patients, which facilitated LV transduction to be profiled over time. Total VCN/cell peaked at 24 h for L1 and L3 and at 36 h for L2. Similar 24-h peaks have been reported in hematopoietic stem cells transduced with LV *ex vivo*.[52] Integration was detectable by 12 h after dosing in all livers. Livers 1 and 2 exhibited a rise and fall, with peak integration at 24 and 36 h, respectively, while L3 showed a continued increase until 48 h. The explanation for the peak and subsequent decline in integrated genomes remains unclear. It may be due to immune removal of transduced cells or shedding of KCs, which primarily clear particles entering the liver. It may also be the consequence of initially improved perfusion of this lobe; however, the variability in the three separately taken biopsies suggests the high mean value may be skewed by one anomalous reading. Although the initial pathology of the livers, the perfusion durations, and the consequent infection profiles differed across the replicates, the average total and integrated copy numbers in tissue were remarkably similar, equivalent to between 7 and 13% of cells being transduced by the end of perfusion with no statistically significant difference. It was noticeable that deeper tissue analysis of L3 produced a lower LV copy number than surface sampling with a biopsy needle. In future studies we advise that needle biopsy should be the standardized sampling technique throughout perfusion.

By end of perfusion GFP expression was observed in all livers. Highest expression level was observed in L2, likely a result of the longer perfusion duration achieved. Application of more cell-specific stains and flow cytometry methods may better elucidate cell types successfully expressing the transgene and enable better assessment of vector transduction specificity. Comparison of the GFP % positivity (up to 4%) with the integrated VCN percentage (7–13%) suggests that there may be a pool of non-hepatocyte cells with integrated LV but without the capacity to express the GFP transgene under the control of the hepatocyte-specific enhanced transthyretin (ET) promotor.[49] GFP expression was observed in CD31-positive cells of L3 alone, suggesting tightness of promoter control may vary between patients. Endothelia in L3 may also have been more permissive to the vector than for other livers, enabling a higher uptake by liver sinusoidal

endothelial cells (LSECs) and contributing toward L3's rapid plasma clearance and higher peak VCN during perfusion. This supports the continuation of research efforts to achieve consistent retargeting of LV at both the level of LV interaction with the cell surface (i.e., through tropism modification)[16,53,54] as well as by engineering for enhanced promoter selectivity.[55–57]

Filtrate from the hemoconcentrator was also sampled to titrate inflammatory cytokines and gauge the immune environment of the perfused livers. A peak in inflammatory cytokines was observed at 4 h after dosing (Figure 4), possibly in response to vector delivery. Peak concentrations of IL-1β, IL-6, and IL-8 were higher than those reported at similar time points in non-dosed perfusion studies.[31,58] In recently published clinical trial data whereby a CAR-T LV was administered IV, three of four patients exhibited spikes in plasma IL-6 at 24 or 48 h, reaching between 300 and 12,000 pg/mL, a similar range to the 24 h samples here.[59] In that trial, cytokines were not measured at 4 h after dosing; however, immediately after LV infusion all patients developed acute inflammatory reactions, with three patients developing cytokine release syndrome, suggesting high levels of IL-6 (and other cytokine) release may have occurred shortly after dosing as was observed in this perfused liver model.

In our study, while L1 demonstrated consistently high levels of IL-6 and IL-8, L2 and L4 exhibited a decrease at 12–24 h followed by raised IL-1, IL-6, IL-8, and TNF-α at the end of perfusion, potentially indicating the deteriorating and inflamed condition of the liver that coincides with a breakdown in function as indicated by increasing lactate. Many cytokines, for example, IL-6, are implicated in both the liver's acute phase response to damage as well as immune response to infection,[60,61] and discerning between the underlying impact of surgical and reperfusion injury and the impact of LV infection in these circumstances can be challenging.

From Figures 2G and 2H it is clear that initial AST and ALT rise substantially between pre-LV dose levels and 24 h post-dosing. It is expected that these damage markers would be higher than in a normal healthy patient: the livers have been surgically recovered and spent over 12 h without circulation. Furthermore, the livers were all rejected for transplant precisely because of their suboptimal condition (Table 1), for example, the presence of steatosis or hematomas; therefore, some transaminase elevation is not unexpected, and levels in excess of 9,000 IU/mL have been observed following transplant with successful patient recovery.[62] The decline evident in transaminase levels of livers L1, L2, and L4 between 24 h and endpoint could be indicative of resolution of hepatocyte health. To address this, we have compared the T1/2 for transaminase removal as published by Kim et al.[63] and found that our T1/2 for ALT matches while our T1/2 for AST is greater than the published value (Table S11). The matching ALT half-life could indicate resolution of hepatocyte health. Indeed, during the clinical trial described by Nasralla et al., which examined liver transplant outcomes following organ preservation by NMP,[20] patient ALT and AST peaked within seven days of transplant but only returned to normal range after six months, so a relatively slow rate of transaminase clearance is not unexpected even when the long-term outcome is positive. What is not so clear is whether initial rises up to 24 h are the consequence of LV dosing and transduction or reperfusion injury. Data from Eshmuminov et al. and Mohamed et al. showing lower transaminase levels at 24 h than reported here indicate that the former is more likely.[34,64]

Inclusion of a non-LV dosed control would have helped definitively separate inflammation and toxicity events caused by LV dosing from those caused by re-perfusion injury. However, multiple studies indicate that perfusion stabilizes cytokines and enzymes reversing transplant and cold storage injury[31,32,58,65]; hence, time and resources were not directed toward collecting further data from non-dosed livers.

Transcriptomics approaches can provide some further insight. Here, it was observed that inflammatory pathways are enriched prior to dosing and toward the end of perfusion as liver viability declines, suggesting that inflammation and liver damage is not solely a result of vector delivery in this system. Viral transcript pathways are enriched throughout but interferon pathways are enriched only at 12 and 72 h, which, with the spike in cytokines observed, suggests that an innate immune response is raised to the vector dose and possibly the expressed transgene later on, but not sufficiently to cause catastrophic inflammation and tissue damage.

It has been demonstrated that NMP livers can provide a great depth of data. In the case of LVs, this includes PK, transduction, integration, transgene expression profiles and transcriptome analysis. However, an organ in isolation presents clear limitations as a model, primarily due to the absence of additional organs and circulatory components. This limits the attainment of biodistribution data, for example, and so the transition away from animal models is still far from complete. The PK data are also unlikely to be truly representative of clearance following standard IV dosing at this stage: additional tissues and circulatory vessels undoubtedly would impact the amount of LV deposited in the liver from the bloodstream. The spleen, for instance, is instrumental in both filtration of pathogens from the bloodstream and initiation of innate and adaptive immune responses and could therefore have a major impact on vector PK and immune impact.[66] In counter to this, it should be noted that, HA or PV infusion, although not ideal for ease of widespread clinical adoption, is becoming an accepted route for dosing with viral-based GT vectors. These routes and the rapid "first-pass" clearance they accentuate closely mimic the dosing performed in our *ex vivo* experiments.

The relatively short perfusion duration achieved is also of consequence. Despite robust methodology ensuring only integrated genomes were measured, we would expect that (in common with reported LV behavior in NHP) over time there would be silencing of expression through viral or transgene-directed immune responses,[16] which are not detected within the limitations of this model. As perfusion technology and methodology improves, it is plausible that

future studies may extend beyond two weeks and provide data concerning waning transgene expression and production of transgene-directed antibodies.

A further limitation of these studies is that the perfusate used was primarily composed of packed red blood cells, meaning complement, white blood cells, and free protein are largely absent. This may be addressed by utilization of whole blood or supplementing the current perfusate with individual components to establish the impact of each. Cabanes-Creus et al. achieved a similar effect by addition of AAV-neutralizing plasma to their perfusions.[21] This ability to add and subtract each key blood component in order to quantify and stratify their impact and importance is a function that no other *in vivo* testing platform permits and certainly could not be achieved in a clinical trial.

The reproducibility of the system remains a major hurdle, stemming from variation in donor patient profile and liver condition, although it could be argued that this is more reflective of the ultimate intended patient population than the use of inbred strains of lab animals. Notably, despite marked difference in donor health and some differences in PK profile, the viral integration results observed in this study exhibit close similarity with no significant difference in total or integrated VCN between livers at the end of perfusion. During perfusion, triplicate biopsies were not always obtained. As these experiments were designed as a proof-of-concept exploration of what can be achieved in terms of sampling, dosing, and analysis, priority was given to providing a thorough examination of the time-series. Hence, the balance between optimizing sampling frequency, blood loss control/liver health, and replicate number was tipped toward the former. With the optimal sample timing now defined future studies can reduce sampling frequency, providing the opportunity to strengthen replicate number and the opportunity for statistical analysis. Some researchers have addressed access to livers and reproducibility by splitting livers, with one-half serving as a matched donor control for the other.[67,68] In the United Kingdom, 1,099 whole livers were donated from deceased donors in the year 2023/24, with 25% of these (273) being rejected for transplant.[69] This suggests a pool of around 250–300 livers available per year. The ability to make best use of this considerable and valuable resource will be reliant on access to perfusion devices and technical skills, which may prove limiting. Furthermore, ongoing improvements to perfusion systems aim to reduce the number of available organs that are deemed unsuitable for transplant.

These data are a proof-of-concept demonstration that helps define perfusion, sampling, and analysis procedures to enable whole human livers to be used for the profiling of LV PK, transduction, and integration. Although a small sample size, this preliminary study has demonstrated remarkable similarity between replicate perfusions of different age and health. This makes an important contribution to the argument that further optimization and ultimately adoption of this approach provides a means to reduce the unsustainable expense, time, and ethical cost associated with pre-clinical testing in rodents, pigs, and NHP. Such moves would be aligned with new regulatory guidance and a growing acceptance that the high attrition rates of therapeutic development can be addressed by moving away from animal models.

MATERIALS AND METHODS

Study design

Organs maintained by NMP present an attractive opportunity for preclinical testing of advanced therapies, potentially bridging the gap between animal models and human patients or replacing animal models entirely. In this study, four whole human livers rejected for transplant were maintained by NMP using a commercially available NMP device and dosed with 5.8×10^{10} TU or 1.16×10^{10} TU of VSV-G-pseudotyped LV containing a GFP reporter transgene under the control of a liver-specific ET promoter. Liver health was monitored by the device's inbuilt sensors and biochemical analysis of the blood-based perfusate. Throughout perfusions, plasma and tissue loads of LV were determined by dPCR and GFP expression assessed by immunofluorescence at the end of perfusion.

Ethical statement

The human livers used in this study were obtained from consenting organ donors and had been deemed unsuitable for transplant. Donor organs were accepted from NHSBT in accordance with a study plan approved by the NHS Health Research Authority (South West – Frenchay Research Ethics Committee, REC reference 20/SW/0133, study number ODT105). Human blood components used in the perfusate were sourced from NHSBT.

Normothermic machine perfusion of whole human livers

Four whole human livers were perfused using a commercially available NMP device with FDA, European, Australian, and Canadian regulatory approvals for liver preservation prior to transplantation (metra, OrganOx Ltd, Oxford, UK). A 40 kDa hemoconcentrator (Medica S.p.A., Medolla, Italy) and two infusion pumps (Alaris SE, Becton, Dickinson and Company, Franklin Lakes, New Jersey, USA) were incorporated for control of perfusate volume and filtration.

Perfusion was started according to the manufacturers' instructions for use. The device was primed according to protocol with 500 mL of Gelofusine colloidal volume replacement solution (B. Braun, Melsungen, Germany) and two units of packed human red blood cells (NC15, NHSBT, Bristol, UK), totaling approximately 1,200 mL of perfusate. After priming, boluses of 500 mg meropenem (Synchrony Pharma Ltd, Stevenage, UK) in 10 mL saline (Baxter International, Deerfield, Illinois, USA), 10,000 units of heparin (Wockhardt, Mumbai, India) in 10 mL saline, and 10 mL of 10% calcium gluconate (DEMO S.A. Pharmaceutical Industry, Athens, Greece) were administered. Infusions of sodium taurocholate (OrganOx Ltd), heparin, epoprostenol (GSK plc, Brentford, UK), and insulin (Novo Nordisk A/S, Bagsværd, Denmark) were continuously administered during perfusion, regulated by the device. Infusion medications were replaced, and the meropenem bolus was repeated every 24 h. Total

parenteral nutrition was provided by attaching Nutriflex Special infusion solution (B. Braun) to the inbuilt nutrition pump.

Livers were received on ice, cannulated, and flushed with cold saline before connecting to the device via the HA, PV, inferior vena cava (IVC), and bile duct.

For the liver-free perfusion described in Figure S1 the NMP device was set up as described above with the same perfusate, boluses, and infusions, but in place of a liver a Y-connector attached the HA and PV tubing directly to the IVC.

Liver function and hemodynamics

The NMP device recorded flow rates and pressures from the HA, PV, and IVC, and perfusate pH, pO2, pCO2, temperature, and bile production. Bile production is not included in this study as collection for LV quantification interrupted measurement of bile flow. Gas and biochemistry in the perfusate were measured using an i-STAT1 blood analyzer with CG4+ and CG8+ cartridges (Abbott, Green Oaks, Illinois, USA) and a Piccolo Xpress blood analyzer with Piccolo Comprehensive Metabolic Panel cartridge (Abaxis, Union City, California, USA). AST, ALT, and ALP were quantified by John Radcliffe Hospital clinical biochemistry laboratories. Glucose concentration was also measured with GlucoRx Nexus Blood Meter test strips (GlucoRx, Guildford, UK).

Lentiviral vector

A non-replicative HIV-based LV encoding GFP under control of a liver-specific promoter was supplied by Oxford Biomedica (UK) Ltd. The vector was stored at −80°C until use.

LV administration and plasma, bile, filtrate, and tissue sampling

5.8×10^{10} TU or 1.16×10^{10} TU of LV were administered as a 20 mL bolus via a two-way stopcock in the HA cannula over a period of approximately 10 s.

Five milliliters of perfusate was collected in BD Vacutainer Lithium Heparin blood collection tubes (Becton, Dickinson and Company) throughout perfusion. After 30–120 min of incubation at room temperature, the perfusate was centrifuged and the plasma fraction was collected and stored at −80°C. Every 12 h, 1 mL of bile and 1 mL of filtrate were collected and stored at −80°C. Tissue core biopsies were collected from the right lobe prior to onboarding and every 12 h during perfusion using a BioPince Ultra Full Core Biopsy Instrument (Argon Medical Devices, Plano, Texas, USA). Biopsies were stored in RNALater (Thermo Fisher Scientific, Waltham, Massachusetts, USA) at room temperature for 24 h before snap freezing with liquid nitrogen and storing at −80°C. After perfusion, ~2 cm^3 biopsies from each lobe were stored in RNALater or 10% neutral-buffered formalin. Tissue in RNALater was incubated at room temperature for 24 h, snap frozen with liquid nitrogen, and stored at −80°C. For L1, tissue was incubated in formalin at room temperature for 24 h before transfer to 30% sucrose and incubation at 4°C for 4 days. After sucrose incubation, tissue was transferred to plastic sample holder, coated in O.C.T. compound and snap frozen with liquid nitrogen. For L2-L4, after at least 24 h in formalin tissue was paraffin embedded.

Quantification of LV RNA genomes in perfusate and bile

Perfusate, 0.424 mL, or 1 mL bile was centrifuged at full speed at 4°C for 30 min. The supernatant was discarded and the pellet resuspended in 140 μL DPBS (Gibco, Thermo Fisher Scientific). Subsequent RNA extraction and DNA removal were performed according to kit protocols (QIAamp Viral RNA Mini Kit 52904, QIAGEN, Hilden, Germany; DNA-free DNA Removal Kit AM1906, Thermo Fisher Scientific) with elution into 80 μL Buffer AVE. LV RNA genomes were quantified by reverse-transcription digital PCR (RT-dPCR) (QIAcuity One, QIAGEN) using the QIAcuity Probe PCR Kit (250101, QIAGEN) and a 26k 24-well nanoplate (250001, QIAGEN) with primers targeting the HIV packaging sequence (Table S12).

Vector half-life in perfusate was calculated using GraphPad Prism analysis tools.

Quantification of active virus in perfusate

LV activity in plasma was determined using serial passaging.[36] Twelve-well plates were seeded with 90,000 HEK293T cells per well in 1 mL growth medium (DMEM 10% fetal bovine serum 1% penicillin-streptomycin) and incubated at 37°C, 5% CO_2. On the following day cells from two wells were removed with trypsin and counted. Media were removed from wells and replaced with 500 μL of 1:1 plasma:media mix and plates returned to incubation. About 3–6 h later wells were supplemented with an additional 1 mL growth medium. After 3 days incubation cells were passaged. A further two passages were performed, with the final transferring cells into 6-well plates. Once the wells of the final passage achieved confluency, cells were removed by trypsin, pelleted, resuspended in 200 μL DPBS and stored at −80°C until DNA extraction. DNA extraction of cell pellets was performed using PureLink Genomic DNA Mini Kit (K182002, Thermo Fisher Scientific) according to kit protocol. LV DNA genomes and the cellular gene *RPPH1* were quantified via dPCR. There are two copies of *RPPH1* per cell, so the total VCN per cell was calculated using Equation 1.

$$\text{Vector Copy Number per Cell} = 2 \times \frac{\text{LV genome copies}}{\textit{RPPH1}\text{ copies}} \quad \text{(Equation 1)}$$

The VCN/cell was then used in conjunction with the cell numbers at infection and infection volume to calculate the TU present in each plasma sample. Primer and probe sequences for the HIV packaging sequence and *RPPH1* are defined in supplementary materials Table S12.

Quantification of LV DNA genomes in tissue

DNA extraction was performed using the DNeasy Blood and Tissue Kit (69504, QIAGEN). Tissue samples were weighed and normalized

to 23 ± 1.5 mg. When <21.5 mg was available, all available tissue was used. Tissue was minced and incubated overnight with 180 μL of Buffer ATL (939011, QIAGEN) and 20 μL of proteinase K at 65°C with shaking at 750 rpm. DNA was then extracted according to kit protocol. LV DNA genomes and the cellular gene *RPPH1* were quantified via dPCR. VCN/cell was calculated using the formula previously described, whereby total VCN/cell is the total number of DNA LV genome copies (both integrated and non-integrated) per cellular genome and integrated VCN/cell is the number of DNA LV genome copies integrated into cellular genomes (per cellular genome) as quantified by the following method.

To quantify integrated VCN/cell, extracted DNA underwent gel electrophoresis size exclusion with a 15KB cut-off (PippinHT with 0.75% agarose high-pass cassette, Sage Science, Beverly, Massachusetts, USA) to isolate genomic DNA and exclude non-integrated LV genomes. VCN/cell was then quantified via dPCR.

Tissue immunofluorescent imaging

For L1 cryopreserved samples, 10 μm sections were produced with a cryostat. Tissue sectioning of L2-L4 samples and staining and imaging of all sections were performed by the University of Oxford Cancer Translational Histopathology Laboratory. Four micrometer sections were produced from formalin-fixed paraffin-embedded (FFPE) samples. Sections were stained with DAPI, anti-GFP (ab183734, Abcam, Cambridge, UK), and anti-CD31 (M0823, Agilent, Santa Clara, California, USA). Anti-GFP was diluted 1/100 and incubated for 45 min, then antigen retrieval was performed with PH9 according to the Leica BOND retrieval protocol. Anti-CD31 was diluted 1/500 and incubated for 30 min, then antigen retrieval was performed with PH6 as per the Leica BOND retrieval protocol. Imaging was performed with PhenoImager HT (Akoya Biosciences, Marlborough, Massachusetts, USA).

GFP spatial distribution analysis

Distribution of GFP across the liver lobule was assessed using the large images seen in Figure S6 in the image analysis software ImageJ.[70,71] For each image, portal triads were identified, and rectangular regions of interest were selected between the PV and a nearby central vein, spanning the lobule axis. In the GFP channel, fluorescence intensity (gray values) was measured against distance along the axis from portal to central vein. Distance was normalized to a scale of 0 (PV) to 100 (central vein). This was repeated for multiple regions across the image, and gray values were averaged.

Tissue RNA extraction and transcriptomics

Frozen tissue samples in RNALater were thawed at room temperature and weighed and transferred to fresh microcentrifuge tubes on ice. A limited number of replicates were used (0 h n = 2, 12 h n = 1, 24 h n = 2, 48 h n = 2, 72 h n = 2) to limit bleeding during perfusion and due to loss of 12 h replicate during sample processing. Total RNA extraction was performed using the PureLink RNA Mini Kit (12183018A Thermo Fisher Scientific) according to kit protocol, using microtube pestles and syringes for tissue lysis and homogenization. DNA removal was performed using the DNA-free DNA Removal Kit (AM1906 Thermo Fisher Scientific), and the eluted RNA was stored at −20°C.

Strand-specific mRNA sequencing was performed by Novogene (UK) Co. Ltd. Read pseudoalignment was performed with Kallisto[72] using the *Homo sapiens* GRCh38.p14 genome assembly from Ensembl as a reference.[73] Following alignment, transcripts were annotated using Bioconductor Ensembl based annotation package (EnsDb.Hsapiens.v86) in R.[74] Following filtering of low counts and normalization, GSVA was performed using the Hallmark (H) and Reactome (C2:CP:REACTOME) curated gene sets.[40,75] For presented data, Reactome gene sets were filtered for those containing the terms “HIV” and “Viral.”

Statistical analysis

Descriptive statistics (mean and standard deviation) of continuous outcome measurements such as arterial flow rates or VCN/cell are presented in the text. Differences in total and integrated VCN/cell at endpoint were assessed by one-way ANOVA, and differences in total and integrated VCN/cell between lobes were assessed by one-way ANOVA followed by the Tukey multiple comparisons test using GraphPad Prism. Vector half-life in plasma was calculated using GraphPad Prism’s linear regression tool to fit two-phase exponential decay models, and GraphPad was also used to calculate the area under the curve. C_{max} and T_{max} were also calculated.

DATA AND CODE AVAILABILITY

Data will be made available upon reasonable request.

ACKNOWLEDGMENTS

We thank Mandy Townsend from the Institute of Biomedical Engineering at the University of Oxford for her assistance with tissue sample preservation and sectioning.

We also thank the Oxford Cancer Translational Histopathology Laboratory for their assistance with sample sectioning, staining, and immunofluorescent imaging.

R.C.C. and C.C.C. are grateful for the generous benefaction of Mr. Donald Porteous who supports their research.

B.R.M.N. is supported by the BBSRC Studentship Advanced Bioscience of Viral Products, grant number BB/Y51343X/1.

The work was funded by a UKRI BBSRC and Oxford Biomedica (OXB)-funded Advanced Bioscience of Viral Products (ABViP) Collaborative Training Partnership (CTP), grant number: BB/W009420/1. ABViP is a doctoral training program between Oxford Biomedica, University College London, and the University of Oxford and includes financial and in-kind support from the UK university partners.

AUTHOR CONTRIBUTIONS

Conceptualization: C.C.C., K.A.M., R.C.C.; methodology: R.C.C., B.R.M.N., C.C.C., K.A.M., A.K., R.A.S.R.; investigation: B.R.M.N., D.J., A.K., R.A.S.R.; visualization: R.C.C., D.J., C.C.C., K.A.M.; funding acquisition: C.C.C., R.C.C., K.A.M.; project administration: R.C.C., K.A.M., A.K., C.C.C.; supervision: C.C.C., R.C.C.; writing – original draft: B.R.M.N., R.C.C.; writing – review & editing: B.R.M.N., R.C.C., C.C.C., K.A.M., A.K., R.A.S.R.

DECLARATION OF INTERESTS

The work was funded by Oxford Biomedica (UK) Ltd. K.A.M., A.K., and R.A.S.R. are employees of Oxford Biomedica (UK) Ltd and received compensation in the form of salary and stock options.

In addition to his academic role as chair of biomedical engineering, C.C.C. is also a founder, director, and shareholder and receives consultancy income from OrganOx Ltd.

SUPPLEMENTAL INFORMATION

Supplemental information can be found online at https://doi.org/10.1016/j.omta.2025.201660.

REFERENCES

1. Bulcha, J.T., Wang, Y., Ma, H., Tai, P.W.L., and Gao, G. (2021). Viral vector platforms within the gene therapy landscape. Signal Transduct. Target. Ther. *6*, 53. https://doi.org/10.1038/s41392-021-00487-6.
2. WHO (2025). Global Health Observatory HIV Data (World Health Organisation). https://www.who.int/data/gho/data/themes/hiv-aids.
3. Calcedo, R., Morizono, H., Wang, L., McCarter, R., He, J., Jones, D., Batshaw, M.L., and Wilson, J.M. (2011). Adeno-associated virus antibody profiles in newborns, children, and adolescents. Clin. Vaccine Immunol. *18*, 1586–1588. https://doi.org/10.1128/CVI.05107-11.
4. Calcedo, R., Vandenberghe, L.H., Gao, G., Lin, J., and Wilson, J.M. (2009). Worldwide epidemiology of neutralizing antibodies to adeno-associated viruses. J. Infect. Dis. *199*, 381–390. https://doi.org/10.1086/595830.
5. Klamroth, R., Hayes, G., Andreeva, T., Gregg, K., Suzuki, T., Mitha, I.H., Hardesty, B., Shima, M., Pollock, T., Slev, P., et al. (2022). Global Seroprevalence of Pre-existing Immunity Against AAV5 and Other AAV Serotypes in People with Hemophilia A. Hum. Gene Ther. *33*, 432–441. https://doi.org/10.1089/hum.2021.287.
6. DePolo, N.J., Reed, J.D., Sheridan, P.L., Townsend, K., Sauter, S.L., Jolly, D.J., and Dubensky, T.W., Jr. (2000). VSV-G pseudotyped lentiviral vector particles produced in human cells are inactivated by human serum. Mol. Ther. *2*, 218–222. https://doi.org/10.1006/mthe.2000.0116.
7. Milani, M., Annoni, A., Bartolaccini, S., Biffi, M., Russo, F., Di Tomaso, T., Raimondi, A., Lengler, J., Holmes, M.C., Scheiflinger, F., et al. (2017). Genome editing for scalable production of alloantigen-free lentiviral vectors for in vivo gene therapy. EMBO Mol. Med. *9*, 1558–1573. https://doi.org/10.15252/emmm.201708148.
8. Tesfay, M.Z., Ammayappan, A., Federspiel, M.J., Barber, G.N., Stojdl, D., Peng, K.W., and Russell, S.J. (2014). Vesiculovirus neutralization by natural IgM and complement. J. Virol. *88*, 6148–6157. https://doi.org/10.1128/JVI.00074-14.
9. FDA (2025). Approved Cellular and Gene Therapy Products (US Food and Drug Administration). https://www.fda.gov/vaccines-blood-biologics/cellular-gene-therapy-products/approved-cellular-and-gene-therapy-products.
10. European Medicines Agency Committee for Advanced Therapies (CAT) (2025). CAT quarterly highlights and approved ATMPs - May 2025. https://www.ema.europa.eu/en/documents/committee-report/cat-quarterly-highlights-approved-atmps-may-2025_en.pdf.
11. Mullard, A. (2024). In vivo CAR T cells move into clinical trials. Nat. Rev. Drug Discov. *23*, 727–730. https://doi.org/10.1038/d41573-024-00150-z.
12. Quaglia, A., Roberts, E.A., and Torbenson, M. (2018). Developmental and Inherited Liver Disease. In Macsween's Pathology of the Liver, A.D. Burt, L.D. Ferrell, and S.G. Hübscher, eds. (Elsevier), pp. 111–274. https://doi.org/10.1016/b978-0-7020-6697-9.00003-0.
13. Ganesan, L.P., Mohanty, S., Kim, J., Clark, K.R., Robinson, J.M., and Anderson, C.L. (2011). Rapid and efficient clearance of blood-borne virus by liver sinusoidal endothelium. PLoS Pathog. *7*, e1002281. https://doi.org/10.1371/journal.ppat.1002281.
14. Noda, M., Tatsumi, K., Matsui, H., Matsunari, Y., Sato, T., Fukuoka, Y., Hotta, A., Okano, T., Kichikawa, K., Sugimoto, M., et al. (2021). Development of alternative gene transfer techniques for ex vivo and in vivo gene therapy in a canine model. Regen. Ther. *18*, 347–354. https://doi.org/10.1016/j.reth.2021.08.009.
15. Nicolas, C.T., VanLith, C.J., Hickey, R.D., Du, Z., Hillin, L.G., Guthman, R.M., Cao, W.J., Haugo, B., Lillegard, A., Roy, D., et al. (2022). In vivo lentiviral vector gene therapy to cure hereditary tyrosinemia type 1 and prevent development of precancerous and cancerous lesions. Nat. Commun. *13*, 5012. https://doi.org/10.1038/s41467-022-32576-7.
16. Milani, M., Canepari, C., Liu, T., Biffi, M., Russo, F., Plati, T., Curto, R., Patarroyo-White, S., Drager, D., Visigalli, I., et al. (2022). Liver-directed lentiviral gene therapy corrects hemophilia A mice and achieves normal-range factor VIII activity in non-human primates. Nat. Commun. *13*, 2454. https://doi.org/10.1038/s41467-022-30102-3.
17. Baruteau, J., Waddington, S.N., Alexander, I.E., and Gissen, P. (2017). Gene therapy for monogenic liver diseases: clinical successes, current challenges and future prospects. J. Inherit. Metab. Dis. *40*, 497–517. https://doi.org/10.1007/s10545-017-0053-3.
18. Mullard, A. (2016). Parsing clinical success rates. Nat. Rev. Drug Discov. *15*, 447. https://doi.org/10.1038/nrd.2016.136.
19. Harrison, R.K. (2016). Phase II and phase III failures: 2013-2015. Nat. Rev. Drug Discov. *15*, 817–818. https://doi.org/10.1038/nrd.2016.184.
20. Nasralla, D., Coussios, C.C., Mergental, H., Akhtar, M.Z., Butler, A.J., Ceresa, C.D.L., Chiocchia, V., Dutton, S.J., García-Valdecasas, J.C., Heaton, N., et al. (2018). A randomized trial of normothermic preservation in liver transplantation. Nature *557*, 50–56. https://doi.org/10.1038/s41586-018-0047-9.
21. Cabanes-Creus, M., Liao, S.H.Y., Gale Navarro, R., Knight, M., Nazareth, D., Lau, N.S., Ly, M., Zhu, E., Roca-Pinilla, R., Bugallo Delgado, R., et al. (2024). Harnessing whole human liver ex situ normothermic perfusion for preclinical AAV vector evaluation. Nat. Commun. *15*, 1876. https://doi.org/10.1038/s41467-024-46194-y.
22. Kim, J.J., Kurial, S.N.T., Choksi, P.K., Nunez, M., Lunow-Luke, T., Bartel, J., Driscoll, J., Her, C.L., Dhillon, S., Yue, W., et al. (2025). AAV capsid prioritization in normal and steatotic human livers maintained by machine perfusion. Nat. Biotechnol. *43*, 1966–1978. https://doi.org/10.1038/s41587-024-02523-6.
23. Mergental, H., Stephenson, B.T.F., Laing, R.W., Kirkham, A.J., Neil, D.A.H., Wallace, L.L., Boteon, Y.L., Widmer, J., Bhogal, R.H., Perera, M.T.P.R., et al. (2018). Development of Clinical Criteria for Functional Assessment to Predict Primary Nonfunction of High-Risk Livers Using Normothermic Machine Perfusion. Liver Transpl. *24*, 1453–1469. https://doi.org/10.1002/lt.25291.
24. Crawford, J.M., Bioulac-Sage, P., and Hytiroglou, P. (2018). Structure, Function, and Responses to Injury. In Macsween's Pathology of the Liver, A.D. Burt, L.D. Ferrell, and S.G. Hübscher, eds. (Elsevier), pp. 1–87. https://doi.org/10.1016/b978-0-7020-6697-9.00001-7.
25. Hann, A., Lembach, H., Nutu, A., Mergental, H., Isaac, J.L., Isaac, J.R., Oo, Y.H., Armstrong, M.J., Rajoriya, N., Afford, S., et al. (2022). Assessment of Deceased Brain Dead Donor Liver Grafts via Normothermic Machine Perfusion: Lactate Clearance Time Threshold Can Be Safely Extended to 6 Hours. Liver Transpl. *28*, 493–496. https://doi.org/10.1002/lt.26317.
26. Lala, V., Zubair, M., and Minter, D. (2023). Liver Function Tests (StatPearls Publishing).
27. Chapman, W.C., Barbas, A.S., D'Alessandro, A.M., Vianna, R., Kubal, C.A., Abt, P., Sonnenday, C., Barth, R., Alvarez-Casas, J., Yersiz, H., et al. (2023). Normothermic Machine Perfusion of Donor Livers for Transplantation in the United States: A Randomized Controlled Trial. Ann. Surg. *278*, e912–e921. https://doi.org/10.1097/SLA.0000000000005934.
28. ALP - blood test (2025). US National Library of Medicine. https://medlineplus.gov/ency/article/003470.htm.
29. (2025). Aspartate aminotransferase (AST) blood test. (US National Library of Medicine). https://medlineplus.gov/ency/article/003472.htm.
30. (2025). Alanine transaminase (ALT) blood test. (US National Library of Medicine). https://medlineplus.gov/ency/article/003473.htm.
31. Eshmuminov, D., Becker, D., Bautista Borrego, L., Hefti, M., Schuler, M.J., Hagedorn, C., Muller, X., Mueller, M., Onder, C., Graf, R., et al. (2020). An integrated perfusion machine preserves injured human livers for 1 week. Nat. Biotechnol. *38*, 189–198. https://doi.org/10.1038/s41587-019-0374-x.
32. Watson, C.J.E., Kosmoliaptsis, V., Randle, L.V., Gimson, A.E., Brais, R., Klinck, J.R., Hamed, M., Tsyben, A., and Butler, A.J. (2017). Normothermic Perfusion in the Assessment and Preservation of Declined Livers Before Transplantation:

Hyperoxia and Vasoplegia-Important Lessons From the First 12 Cases. Transplantation *101*, 1084–1098. https://doi.org/10.1097/TP.0000000000001661.

33. Watson, C.J.E., Kosmoliaptsis, V., Pley, C., Randle, L., Fear, C., Crick, K., Gimson, A.E., Allison, M., Upponi, S., Brais, R., et al. (2018). Observations on the ex situ perfusion of livers for transplantation. Am. J. Transplant. *18*, 2005–2020. https://doi.org/10.1111/ajt.14687.
34. Mohamed, A., Shamaa, T., Francis, I., Crombez, C., Cui, J., Theisen, B.K., Lopez-Plaza, I., Nagai, S., Collins, K., Yoshida, A., et al. (2023). 24-hour normothermic machine perfusion of discarded human liver grafts: Case series single-center study. J. Liver Transplant. *12*, 100177. https://doi.org/10.1016/j.liver.2023.100177.
35. Galvin, Z., McDonough, A., Ryan, J., and Stewart, S. (2015). Blood alanine aminotransferase levels >1,000 IU/l - causes and outcomes. Clin. Med. *15*, 244–247. https://doi.org/10.7861/clinmedicine.15-3-244.
36. Moore-Kelly, C., Reddem, R., Alberts, B.M., Wright, J., Evans, T., Kulkarni, A., Clarkson, N.G., Farley, D.C., Mitrophanous, K.A., and Saraiva Raposo, R.A. (2025). Enhancing titers of therapeutic lentiviral vectors using PKC agonists. Mol. Ther. Methods Clin. Dev. *33*, 101484. https://doi.org/10.1016/j.omtm.2025.101484.
37. Lada, S.M., Huang, K., VanBelzen, D.J., Montaner, L.J., O'Doherty, U., and Richman, D.D. (2018). Quantitation of Integrated HIV Provirus by Pulsed-Field Gel Electrophoresis and Droplet Digital PCR. J. Clin. Microbiol. *56*, e01158-18. https://doi.org/10.1128/jcm.01158-01118.
38. Wang, M.J., Zhang, H.L., Chen, F., Guo, X.J., Liu, Q.G., and Hou, J. (2024). The double-edged effects of IL-6 in liver regeneration, aging, inflammation, and diseases. Exp. Hematol. Oncol. *13*, 62. https://doi.org/10.1186/s40164-024-00527-1.
39. Wu, Y., Feng, W., Liu, R., Xia, T., and Liu, S. (2020). Graphene Oxide Causes Disordered Zonation Due to Differential Intralobular Localization in the Liver. ACS Nano *14*, 877–890. https://doi.org/10.1021/acsnano.9b08127.
40. Subramanian, A., Tamayo, P., Mootha, V.K., Mukherjee, S., Ebert, B.L., Gillette, M.A., Paulovich, A., Pomeroy, S.L., Golub, T.R., Lander, E.S., and Mesirov, J.P. (2005). Gene set enrichment analysis: a knowledge-based approach for interpreting genome-wide expression profiles. Proc. Natl. Acad. Sci. USA *102*, 15545–15550. https://doi.org/10.1073/pnas.0506580102.
41. Liberzon, A., Birger, C., Thorvaldsdóttir, H., Ghandi, M., Mesirov, J.P., and Tamayo, P. (2015). The Molecular Signatures Database (MSigDB) hallmark gene set collection. Cell Syst. *1*, 417–425. https://doi.org/10.1016/j.cels.2015.12.004.
42. Liberzon, A., Subramanian, A., Pinchback, R., Thorvaldsdóttir, H., Tamayo, P., and Mesirov, J.P. (2011). Molecular signatures database (MSigDB) 3.0. Bioinformatics *27*, 1739–1740. https://doi.org/10.1093/bioinformatics/btr260.
43. van Leeuwen, O.B., de Vries, Y., Fujiyoshi, M., Nijsten, M.W.N., Ubbink, R., Pelgrim, G.J., Werner, M.J.M., Reyntjens, K.M.E.M., van den Berg, A.P., de Boer, M.T., et al. (2019). Transplantation of High-risk Donor Livers After Ex Situ Resuscitation and Assessment Using Combined Hypo- and Normothermic Machine Perfusion: A Prospective Clinical Trial. Ann. Surg. *270*, 906–914. https://doi.org/10.1097/SLA.0000000000003540.
44. Mergental, H., Laing, R.W., Kirkham, A.J., Perera, M.T.P.R., Boteon, Y.L., Attard, J., Barton, D., Curbishley, S., Wilkhu, M., Neil, D.A.H., et al. (2020). Transplantation of discarded livers following viability testing with normothermic machine perfusion. Nat. Commun. *11*, 2939. https://doi.org/10.1038/s41467-020-16251-3.
45. Lau, N.S., Ly, M., Dennis, C., Liu, K., Kench, J., Crawford, M., and Pulitano, C. (2022). Long-term normothermic perfusion of human livers for longer than 12 days. Artif. Organs *46*, 2504–2510. https://doi.org/10.1111/aor.14372.
46. Finkelshtein, D., Werman, A., Novick, D., Barak, S., and Rubinstein, M. (2013). LDL receptor and its family members serve as the cellular receptors for vesicular stomatitis virus. Proc. Natl. Acad. Sci. USA *110*, 7306–7311. https://doi.org/10.1073/pnas.1214441110.
47. van Til, N.P., Markusic, D.M., van der Rijt, R., Kunne, C., Hiralall, J.K., Vreeling, H., Frederiks, W.M., Oude-Elferink, R.P.J., and Seppen, J. (2005). Kupffer cells and not liver sinusoidal endothelial cells prevent lentiviral transduction of hepatocytes. Mol. Ther. *11*, 26–34. https://doi.org/10.1016/j.ymthe.2004.09.012.
48. Canepari, C., Milani, M., Simoni, C., Starinieri, F., Volpin, M., Fabiano, A., Biffi, M., Russo, F., Norata, R., Rocchi, M., et al. (2025). Enhancing the potency of in vivo lentiviral vector mediated gene therapy to hepatocytes. Nat. Commun. *16*, 4802. https://doi.org/10.1038/s41467-025-60073-0.
49. Milani, M., Annoni, A., Moalli, F., Liu, T., Cesana, D., Calabria, A., Bartolaccini, S., Biffi, M., Russo, F., Visigalli, I., et al. (2019). Phagocytosis-shielded lentiviral vectors improve liver gene therapy in nonhuman primates. Sci. Transl. Med. *11*, eaav7325. https://doi.org/10.1126/scitranslmed.aav7325.
50. Carbonaro Sarracino, D., Tarantal, A.F., Lee, C.C.I., Martinez, M., Jin, X., Wang, X., Hardee, C.L., Geiger, S., Kahl, C.A., and Kohn, D.B. (2014). Effects of vector backbone and pseudotype on lentiviral vector-mediated gene transfer: studies in infant ADA-deficient mice and rhesus monkeys. Mol. Ther. *22*, 1803–1816. https://doi.org/10.1038/mt.2014.88.
51. Higashi, N., Ueda, H., Yamada, O., Oikawa, S., Koiwa, M., Tangkawattana, P., and Takehana, K. (2002). Micromorphological characteristics of hepatic sinusoidal endothelial cells and their basal laminae in five different animal species. Okajimas Folia Anat. Jpn. *79*, 135–142. https://doi.org/10.2535/ofaj.79.135.
52. Uchida, N., Green, R., Ballantine, J., Skala, L.P., Hsieh, M.M., and Tisdale, J.F. (2016). Kinetics of lentiviral vector transduction in human CD34(+) cells. Exp. Hematol. *44*, 106–115. https://doi.org/10.1016/j.exphem.2015.10.003.
53. Sinn, P.L., Goreham-Voss, J.D., Arias, A.C., Hickey, M.A., Maury, W., Chikkanna-Gowda, C.P., and McCray, P.B., Jr. (2007). Enhanced gene expression conferred by stepwise modification of a nonprimate lentiviral vector. Hum. Gene Ther. *18*, 1244–1252. https://doi.org/10.1089/hum.2006.127.
54. Chai, N., Chang, H.E., Nicolas, E., Gudima, S., Chang, J., and Taylor, J. (2007). Assembly of hepatitis B virus envelope proteins onto a lentivirus pseudotype that infects primary human hepatocytes. J. Virol. *81*, 10897–10904. https://doi.org/10.1128/JVI.00959-07.
55. Condiotti, R., Curran, M.A., Nolan, G.P., Giladi, H., Ketzinel-Gilad, M., Gross, E., and Galun, E. (2004). Prolonged liver-specific transgene expression by a non-primate lentiviral vector. Biochem. Biophys. Res. Commun. *320*, 998–1006. https://doi.org/10.1016/j.bbrc.2004.06.044.
56. Fama, R., Borroni, E., Merlin, S., Airoldi, C., Pignani, S., Cucci, A., Cora, D., Bruscaggin, V., Scardellato, S., Faletti, S., et al. (2021). Deciphering the Ets-1/2-mediated transcriptional regulation of F8 gene identifies a minimal F8 promoter for hemophilia A gene therapy. Haematologica *106*, 1624–1635. https://doi.org/10.3324/haematol.2019.239202.
57. Ou, L., Przybilla, M.J., Koniar, B.L., and Whitley, C.B. (2016). Elements of lentiviral vector design toward gene therapy for treating mucopolysaccharidosis I. Mol. Genet. Metab. Rep. *8*, 87–93. https://doi.org/10.1016/j.ymgmr.2015.11.004.
58. Lee, A.C.H., Edobor, A., Lysandrou, M., Mirle, V., Sadek, A., Johnston, L., Piech, R., Rose, R., Hart, J., Amundsen, B., et al. (2022). The Effect of Normothermic Machine Perfusion on the Immune Profile of Donor Liver. Front. Immunol. *13*, 788935. https://doi.org/10.3389/fimmu.2022.788935.
59. Xu, J., Liu, L., Parone, P., Xie, W., Sun, C., Chen, Z., Zhang, J., Li, C., Hu, Y., and Mei, H. (2025). In-vivo B-cell maturation antigen CAR T-cell therapy for relapsed or refractory multiple myeloma. Lancet *406*, 228–231. https://doi.org/10.1016/S0140-6736(25)01030-X.
60. Heinrich, P.C., Castell, J.V., and Andus, T. (1990). Interleukin-6 and the acute phase response. Biochem. J. *265*, 621–636. https://doi.org/10.1042/bj2650621.
61. Rose-John, S., Winthrop, K., and Calabrese, L. (2017). The role of IL-6 in host defence against infections: immunobiology and clinical implications. Nat. Rev. Rheumatol. *13*, 399–409. https://doi.org/10.1038/nrrheum.2017.83.
62. Hoyer, D.P., Kaiser, G.M., Treckmann, J.W., Mathe, Z., Saner, F., Radunz, S., Bankfalvi, A., Gallinat, A., Minor, T., and Paul, A. (2013). AST 17600 U/l after liver transplantation, what are you up to? - A case report. Ann. Transplant. *18*, 218–222. https://doi.org/10.12659/AOT.883910.
63. Kim, W.R., Flamm, S.L., Di Bisceglie, A.M., and Bodenheimer, H.C.; Public Policy Committee of the American Association for the Study of Liver Disease (2008). Serum activity of alanine aminotransferase (ALT) as an indicator of health and disease. Hepatology *47*, 1363–1370. https://doi.org/10.1002/hep.22109.
64. Eshmuminov, D., Leoni, F., Schneider, M.A., Becker, D., Muller, X., Onder, C., Hefti, M., Schuler, M.J., Dutkowski, P., Graf, R., et al. (2018). Perfusion settings and additives in liver normothermic machine perfusion with red blood cells as oxygen carrier. A systematic review of human and porcine perfusion protocols. Transpl. Int. *31*, 956–969. https://doi.org/10.1111/tri.13306.

65. Jassem, W., Xystrakis, E., Ghnewa, Y.G., Yuksel, M., Pop, O., Martinez-Llordella, M., Jabri, Y., Huang, X., Lozano, J.J., Quaglia, A., et al. (2019). Normothermic Machine Perfusion (NMP) Inhibits Proinflammatory Responses in the Liver and Promotes Regeneration. Hepatology *70*, 682–695. https://doi.org/10.1002/hep.30475.

66. Bronte, V., and Pittet, M.J. (2013). The spleen in local and systemic regulation of immunity. Immunity *39*, 806–818. https://doi.org/10.1016/j.immuni.2013.10.010.

67. Lau, N.S., Ly, M., Dennis, C., Jacques, A., Cabanes-Creus, M., Toomath, S., Huang, J., Mestrovic, N., Yousif, P., Chanda, S., et al. (2023). Long-term ex situ normothermic perfusion of human split livers for more than 1 week. Nat. Commun. *14*, 4755. https://doi.org/10.1038/s41467-023-40154-8.

68. Lau, N.S., Ly, M., Jacques, A., Ewenson, K., Mestrovic, N., Almoflihi, A., Koutalistras, N., Liu, K., Kench, J., McCaughan, G., et al. (2021). Prolonged Ex Vivo Normothermic Perfusion of a Split Liver: An Innovative Approach to Increase the Number of Available Grafts. Transplant. Direct *7*, e763. https://doi.org/10.1097/TXD.0000000000001216.

69. NHS Blood and Transplant (2024). Organ and Tissue Donation and Transplantation Activity Report 2023/2024 (NHS Blood and Transplant). https://nhsbtdbe.blob.core.windows.net/umbraco-assets-corp/33779/activity-report-2023-2024.pdf.

70. Schindelin, J., Arganda-Carreras, I., Frise, E., Kaynig, V., Longair, M., Pietzsch, T., Preibisch, S., Rueden, C., Saalfeld, S., Schmid, B., et al. (2012). Fiji: an open-source platform for biological-image analysis. Nat. Methods *9*, 676–682. https://doi.org/10.1038/nmeth.2019.

71. Schneider, C.A., Rasband, W.S., and Eliceiri, K.W. (2012). NIH Image to ImageJ: 25 years of image analysis. Nat. Methods *9*, 671–675. https://doi.org/10.1038/nmeth.2089.

72. Bray, N.L., Pimentel, H., Melsted, P., and Pachter, L. (2016). Near-optimal probabilistic RNA-seq quantification. Nat. Biotechnol. *34*, 525–527. https://doi.org/10.1038/nbt.3519.

73. Dyer, S.C., Austine-Orimoloye, O., Azov, A.G., Barba, M., Barnes, I., Barrera-Enriquez, V.P., Becker, A., Bennett, R., Beracochea, M., Berry, A., et al. (2025). Ensembl 2025. Nucleic Acids Res. *53*, D948–D957. https://doi.org/10.1093/nar/gkae1071.

74. Johannes, R.E.D. (2025). Hsapiens.v86: Bioconductor annotation package. Bioconductor. https://doi.org/10.18129/B9.bioc.EnsDb.Hsapiens.v86. https://bioconductor.org/packages/EnsDb.Hsapiens.v86.

75. Mootha, V.K., Lindgren, C.M., Eriksson, K.F., Subramanian, A., Sihag, S., Lehar, J., Puigserver, P., Carlsson, E., Ridderstråle, M., Laurila, E., et al. (2003). PGC-1alpha-responsive genes involved in oxidative phosphorylation are coordinately downregulated in human diabetes. Nat. Genet. *34*, 267–273. https://doi.org/10.1038/ng1180.

OMTA, Volume *34*

Supplemental information

Normothermic perfusion of human livers for profiling lentiviral vector pharmacokinetics and transduction

Brannon R.M. Nicholls, David Johnson, Anurag Kulkarni, Rui André Saraiva Raposo, Kyriacos A. Mitrophanous, Constantin C. Coussios, and Robert C. Carlisle

Supplemental materials

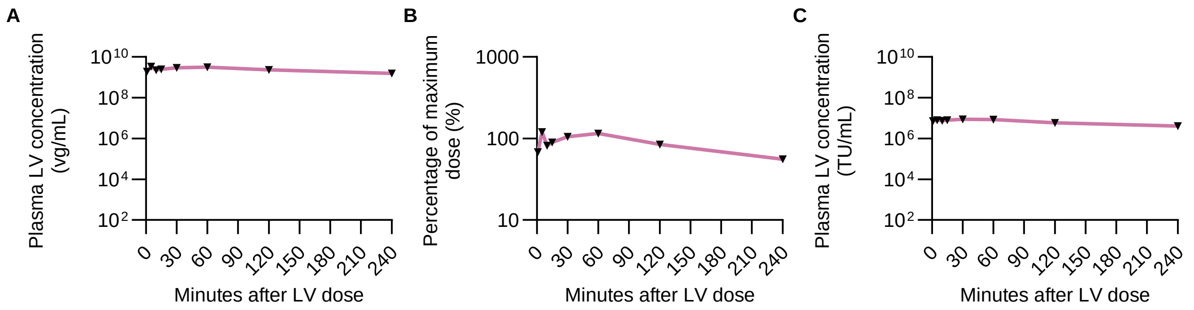

Figure S1. **Pharmacokinetics of LV administered to a liver-free perfusions (A)** LV genome (vg) concentration in plasma fraction of perfusate following administration of 5.8×10^{10} TU to liver-free perfusion system, first time point at 1 minute after dose. **(B)** LV genome concentration in perfusate as a percentage of the theoretical maximum concentration, calculated from measured LV concentration relative to input LV genomes using a perfusate volume of 1200 mL and assuming homogenous distribution of LV in the perfusate. **(C)** LV infectious particle concentration (transducing units (TU)/mL) in perfusate as quantified by *in vitro* infectivity assay.

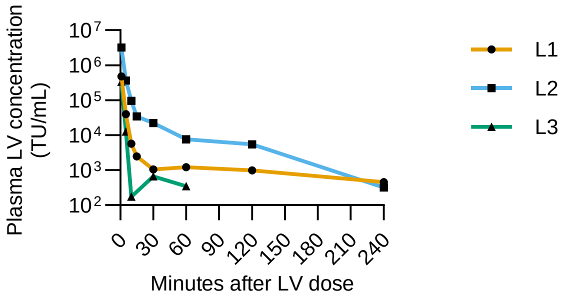

Figure S2. **Clearance of active LV in L1, L2 and L3 plasma.** LV transducing unit concentration (transducing units (TU)/mL) in plasma as quantified by *in vitro* infectivity assay, using plasma collected following administration of 5.8 × 10^{10} TU to perfused livers L1, L2 and L3.

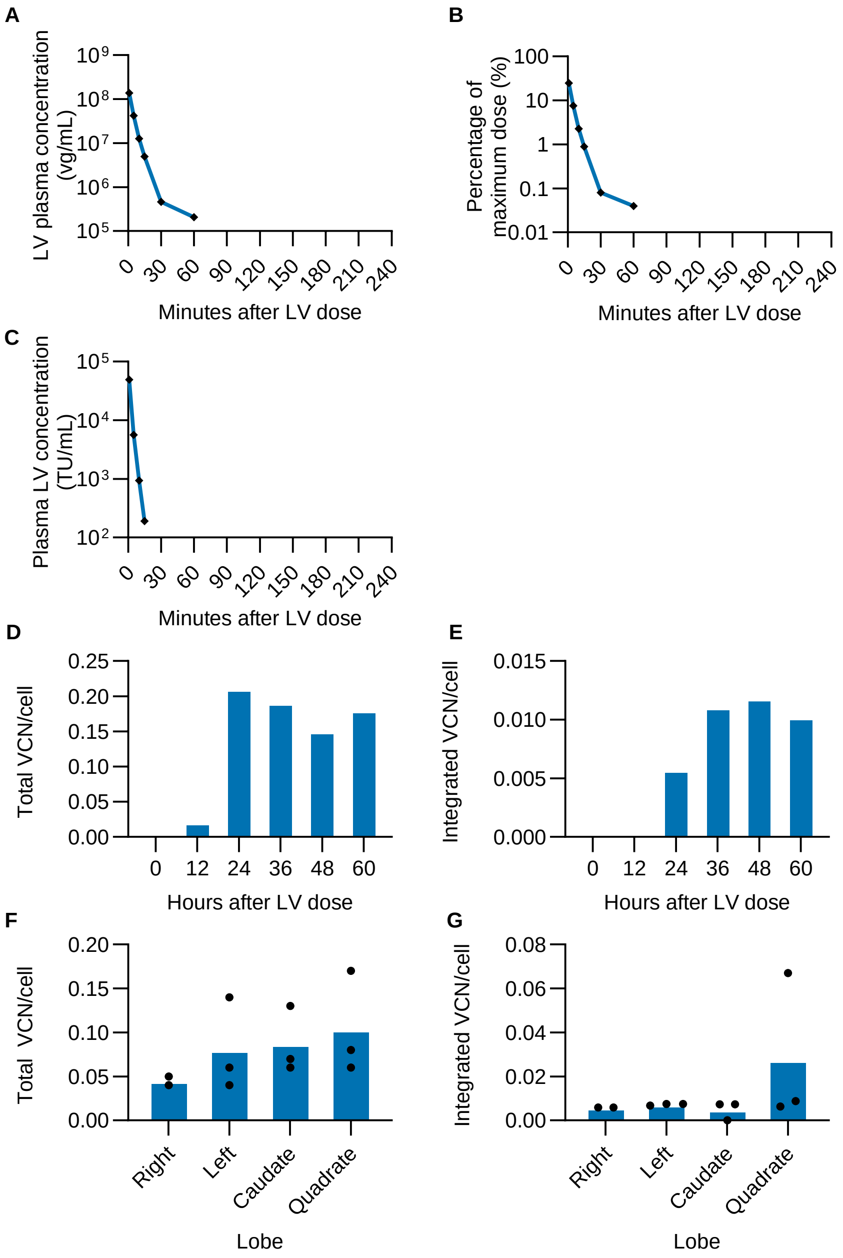

Fig. S3. Pharmacokinetics, integration and distribution of LV administered to perfused liver L4. (A) LV genome (vg) concentration in plasma fraction of perfusate following administration of 1.16 × 10^{10} TU LV to perfused liver L4, first time point at 1 minute after dose. **(B)** LV genome concentration in perfusate as a percentage of the theoretical maximum concentration, calculated from measured LV concentration relative

to input LV genomes using a perfusate volume of 1200 mL and assuming homogenous distribution of LV in the perfusate. **(C)** LV infectious particle concentration (transducing units (TU)/mL) in perfusate as quantified by *in vitro* infectivity assay. **(D)** Total VCN/cell and **(E)** integrated VCN/cell of liver tissue taken from the right lobe of L4 every 12 hours via core biopsies. **(F)** Total VCN/cell and **(G) i**ntegrated VCN/cell of tissue from L4 lobes at the end of perfusion. For **(F)** and **(G),** black circles indicate biopsy technical replicates and bars represent the mean.

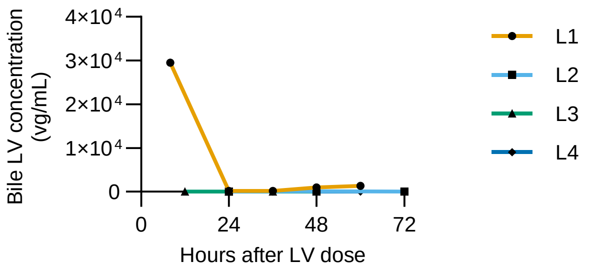

Fig. S4. Vector concentration in bile of perfused livers. LV concentration in bile produced by livers L1, L2, L3 and L4 was quantified by RT-dPCR.

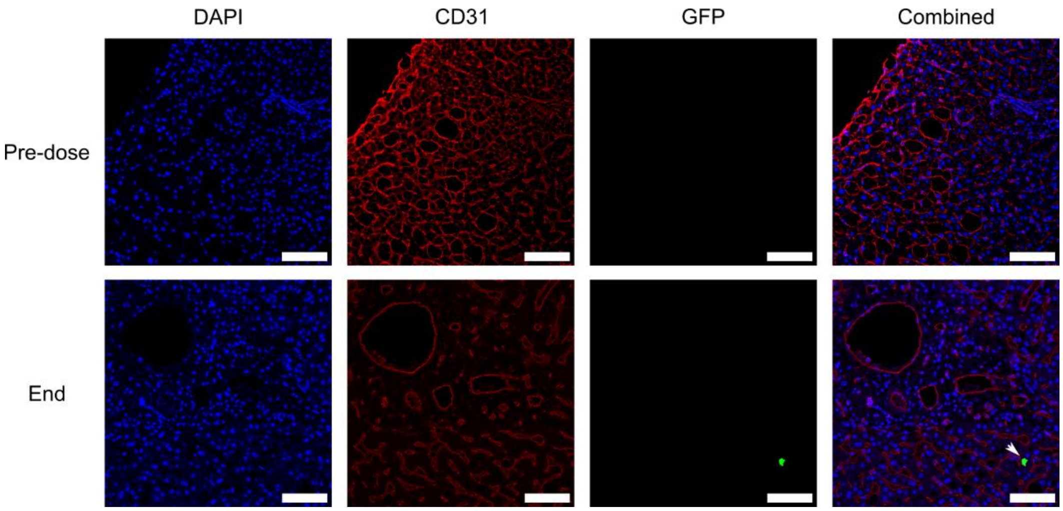

Fig S5. **Liver 4 histology.** Biopsies from L4 before LV dose (Pre-dose) and at the end of perfusion (End) were taken and preserved by FFPE. 4 µm sections were stained with DAPI (blue) and antibodies against CD31 (red) and GFP (green). Scale bar 50 µm. White arrow indicates GFP expressing cell.

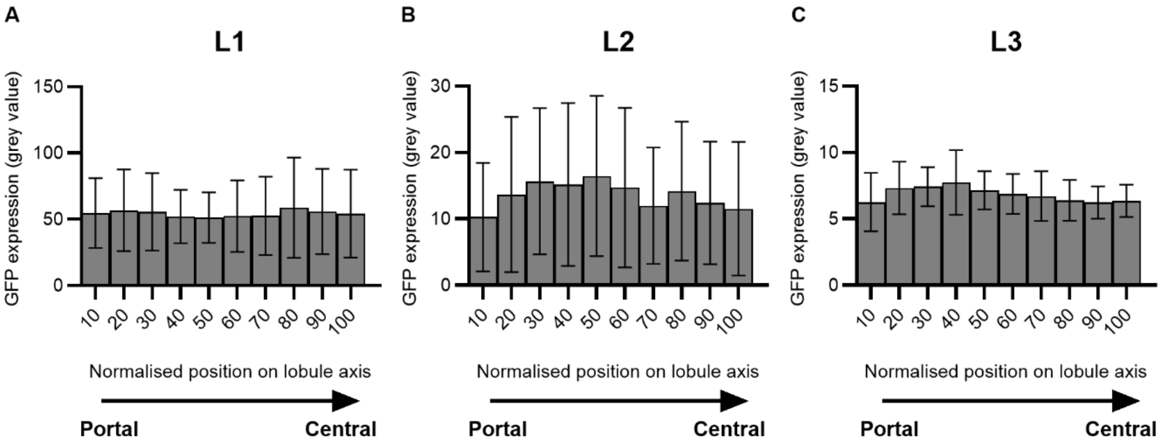

Figure S6. Spatial deposition of GFP expression. GFP expression (grey value) in immunofluorescent images was measured across the lobule axis from portal vein to central vein for livers L1-L3. Multiple axes were measured for each liver and distance normalised to a 0-100 scale, 0 being adjacent to the portal vein and 100 adjacent to the

central vein. Bars represent average grey value in the GFP channel and error bars standard deviation.

Table S1. Pharmacokinetic parameters of L1, L2, L3 and L4. Pharmacokinetic parameters of vector clearance from perfusate were calculated for L1-L4.

Liver	C_{max} (vg/mL)	T_{max} (minutes)	Slow half-life (minutes)	Fast half-life (minutes)	AUC (vg·hours/mL)
L1	9.09×10^8	1	13.20	1.42	5.05×10^7
L2	9.58×10^8	1	13.99	1.31	4.94×10^7
L3	6.44×10^7	1	1.25	1.25	2.80×10^6
L4	1.37×10^8	1	4.95	1.91	9.84×10^6

Table S2. One-way analysis of variance of liver lobe total VCN/cell at endpoint of L1, L2, L3 and L4.

ANOVA table	SS	DF	MS	F (DFn, DFd)	P value
**L1**					
Treatment (between columns)	1.305	3	0.4351	F (3, 7) = 7.874	P=0.0121
Residual (within columns)	0.3869	7	0.05526		
Total	1.692	10			
**L2**					
Treatment (between columns)	0.03420	3	0.01140	F (3, 8) = 0.3923	P=0.7620
Residual (within columns)	0.2325	8	0.02906		
Total	0.2667	11			
**L3**					
Treatment (between columns)	1.393	3	0.4644	F (3, 8) = 52.37	P<0.0001
Residual (within columns)	0.07093	8	0.008867		
Total	1.464	11			
**L4**					
Treatment (between columns)	0.004180	3	0.001393	F (3, 7) = 0.6341	P=0.6163
Residual (within columns)	0.01538	7	0.002198		
Total	0.01956	10			

Table S3. Tukey's multiple comparisons test comparing total VCN/cell of each lobe at endpoint of L1, L2, L3 and L4.

Tukey's multiple comparisons test	Mean diff.	95.00% CI of diff.	Below threshold?	Summary	Adjusted P Value
**L1**					
Right vs. Left	-0.4817	-1.192 to 0.2287	No	ns	0.2009
Right vs. Caudate	-0.07167	-0.7820 to 0.6387	No	ns	0.9861
Right vs. Quadrate	0.4483	-0.2620 to 1.159	No	ns	0.2446
Left vs. Caudate	0.4100	-0.2254 to 1.045	No	ns	0.2306
Left vs. Quadrate	0.9300	0.2946 to 1.565	Yes	**	0.0078
Caudate vs. Quadrate	0.5200	-0.1154 to 1.155	No	ns	0.1097
**L2**					
Right vs. Left	0.01667	-0.4290 to 0.4624	No	ns	0.9993
Right vs. Caudate	-0.07000	-0.5157 to 0.3757	No	ns	0.9561
Right vs. Quadrate	0.08000	-0.3657 to 0.5257	No	ns	0.9369
Left vs. Caudate	-0.08667	-0.5324 to 0.3590	No	ns	0.9219
Left vs. Quadrate	0.06333	-0.3824 to 0.5090	No	ns	0.9668
Caudate vs. Quadrate	0.1500	-0.2957 to 0.5957	No	ns	0.7117
**L3**					
Right vs. Left	0.7933	0.5471 to 1.040	Yes	****	<0.0001
Right vs. Caudate	0.4067	0.1605 to 0.6529	Yes	**	0.0033
Right vs. Quadrate	0.8467	0.6005 to 1.093	Yes	****	<0.0001
Left vs. Caudate	-0.3867	-0.6329 to -0.1405	Yes	**	0.0045
Left vs. Quadrate	0.05333	-0.1929 to 0.2995	No	ns	0.8967
Caudate vs. Quadrate	0.4400	0.1938 to 0.6862	Yes	**	0.0020

**L4**					
Right vs. Left	-0.03500	-0.1767 to 0.1067	No	ns	0.8444
Right vs. Caudate	-0.04167	-0.1833 to 0.09999	No	ns	0.7683
Right vs. Quadrate	-0.05833	-0.2000 to 0.08332	No	ns	0.5567
Left vs. Caudate	-0.006667	-0.1334 to 0.1200	No	ns	0.9979
Left vs. Quadrate	-0.02333	-0.1500 to 0.1034	No	ns	0.9258
Caudate vs. Quadrate	-0.01667	-0.1434 to 0.1100	No	ns	0.9704

Table S4. One-way analysis of variance of liver lobe integrated VCN/cell at endpoint of L1, L2, L3 and L4.

ANOVA table	SS	DF	MS	F (DFn, DFd)	P value
**L1**					
Treatment (between columns)	0.03046	3	0.01015	F (3, 7) = 4.376	P=0.0493
Residual (within columns)	0.01624	7	0.002320		
Total	0.04669	10			
**L2**					
Treatment (between columns)	0.0008978	3	0.0002993	F (3, 8) = 0.6926	P=0.5819
Residual (within columns)	0.003457	8	0.0004321		
Total	0.004354	11			
**L3**					
Treatment (between columns)	0.03727	3	0.01242	F (3, 8) = 8.146	P=0.0082
Residual (within columns)	0.01220	8	0.001525		
Total	0.04947	11			
**L4**					
Treatment (between columns)	0.0009998	3	0.0003333	F (3, 7) = 0.9737	P=0.4573
Residual (within columns)	0.002396	7	0.0003423		
Total	0.003396	10			

Table S5. Tukey's multiple comparisons test comparing integrated VCN/cell of each lobe at endpoint of L1, L2, L3 and L4.

Tukey's multiple comparisons test	Mean diff.	95.00% CI of diff.	Below threshold?	Summary	Adjusted P Value
**L1**					
Right vs. Left	-0.1333	-0.2789 to 0.01221	No	ns	0.0717
Right vs. Caudate	-0.06667	-0.2122 to 0.07887	No	ns	0.4771
Right vs. Quadrate	-0.01153	-0.1571 to 0.1340	No	ns	0.9931
Left vs. Caudate	0.06667	-0.06351 to 0.1968	No	ns	0.3923
Left vs. Quadrate	0.1218	-0.008375 to 0.2520	No	ns	0.0659
Caudate vs. Quadrate	0.05513	-0.07504 to 0.1853	No	ns	0.5360
**L2**					
Right vs. Left	0.01860	-0.03576 to 0.07295	No	ns	0.7019
Right vs. Caudate	0.009388	-0.04496 to 0.06374	No	ns	0.9431
Right vs. Quadrate	0.02231	-0.03204 to 0.07666	No	ns	0.5798
Left vs. Caudate	-0.009207	-0.06356 to 0.04514	No	ns	0.9460
Left vs. Quadrate	0.003712	-0.05064 to 0.05806	No	ns	0.9960
Caudate vs. Quadrate	0.01292	-0.04143 to 0.06727	No	ns	0.8695
**L3**					
Right vs. Left	0.1433	0.04123 to 0.2454	Yes	**	0.0087
Right vs. Caudate	0.07667	-0.02544 to 0.1788	No	ns	0.1534
Right vs. Quadrate	0.1267	0.02456 to 0.2288	Yes	*	0.0173
Left vs. Caudate	-0.06667	-0.1688 to 0.03544	No	ns	0.2345
Left vs. Quadrate	-0.01667	-0.1188 to 0.08544	No	ns	0.9512

Caudate vs. Quadrate	0.05000	-0.05211 to 0.1521	No	ns	0.4456
**L4**					
Right vs. Left	-0.001282	-0.05719 to 0.05462	No	ns	0.9998
Right vs. Caudate	0.001052	-0.05485 to 0.05696	No	ns	>0.9999
Right vs. Quadrate	-0.02141	-0.07731 to 0.03450	No	ns	0.6087
Left vs. Caudate	0.002333	-0.04767 to 0.05234	No	ns	0.9986
Left vs. Quadrate	-0.02012	-0.07013 to 0.02988	No	ns	0.5734
Caudate vs. Quadrate	-0.02246	-0.07246 to 0.02755	No	ns	0.4920

Table S6. Descriptive statistics of total VCN/cell at endpoint of L1, L2 and L3.

Liver	1	2	3
Mean	0.6213	0.5767	0.6750
Std. Deviation	0.3810	0.06164	0.3934

Table S7. One-way analysis of variance of mean total VCN/cell at endpoint of L1, L2 and L3.

ANOVA table	SS	DF	MS	F (DFn, DFd)	P value
Treatment (between columns)	0.01939	2	0.009697	F (2, 9) = 0.09579	P=0.9096
Residual (within columns)	0.9112	9	0.1012		
Total	0.9306	11			

Table S8. Descriptive statistics of integrated VCN/cell at endpoint of L1, L2 and L3.

Liver	1	2	3
Mean	0.1129	0.07792	0.1333
Std. Deviation	0.06102	0.009988	0.06435

Table S9. One-way analysis of variance of mean integrated VCN/cell at endpoint of L1, L2 and L3.

ANOVA table	SS	DF	MS	F (DFn, DFd)	P value
Treatment (between columns)	0.006281	2	0.003140	F (2, 9) = 1.183	P=0.3498
Residual (within columns)	0.02389	9	0.002654		
Total	0.03017	11			

Table S10. Estimated GFP expression in immunofluorescently stained FFPE sections. Total and GFP positive cell counts in 1.3 x 1.3 mm regions of fluorescently stained sections from L1, L2 and L3. No percentage listed for L4: very few GFP cells counted across the entire 1cm^2 section in Fig. S2D.

Liver	Total cells counted	GFP cells counted	Percent GFP expression
L1	8023	50	0.62
L2	4082	165	4.04
L3	3006	10	0.33

Table S11. Comparison of ALT and AST half-life to published values. Half-life of ALT and AST was calculated for L1-L4 and compared to values published by Kim et al.[63]

Liver	ALT T1/2 (hours)	AST T1/2 (hours)
L1	54	108
L2	50	57
L3	N/A	N/A
L4	35	46
Kim et al. 2008	47 +/- 10	17 +/-5

Table S12. Primer sequences for digital PCR quantification of viral RNA and DNA genomes.

Target	Forward primer sequence	Reverse primer sequence	Probe sequence
HIV packaging signal	5’ TGGGCAAGCAGGGAGCTA 3’	5’ TCCTGTCTGAAGGGATGGTTG T 3’	5’ FAM-AACGATTCGCAGTTAATCCTG GCCTGTT-TAMRA 3’
RPPH1	5’ CCCTAGTCTCAGACCTTCCCA AG 3’	5’ GCGGAGGGAAGCTCATCAG 3’	5’ VIC-CCACGAGCTGAGTGCGTCCT GTCA-TAMRA 3’
